# Unlocking high-sulfur-content cathodes by an all-in-one high-entropy sulfide catalyst for all-solid-state Li–S batteries

**DOI:** 10.1093/nsr/nwag501

**Published:** 2026-08-18

**Authors:** Ruqin Ma, Yong Cheng, Mintao Su, Hongxin Lin, Chuanjing Xu, Wenhua Zuo, Siyuan Pan, Gongkai Wang, Yu Luo, Chenpeng Xie, Zhifeng He, Haoyue Zhong, Yuqi Wu, Weilin Huang, Huiyang Gou, Zhengliang Gong, Ming-Sheng Wang, Yong Yang

**Affiliations:** State Key Laboratory for Physical Chemistry of Solid Surface, College of Chemistry and Chemical Engineering, College of Materials, College of Energy, Xiamen University, Xiamen 361005, China; State Key Laboratory for Physical Chemistry of Solid Surface, College of Chemistry and Chemical Engineering, College of Materials, College of Energy, Xiamen University, Xiamen 361005, China; State Key Laboratory for Physical Chemistry of Solid Surface, College of Chemistry and Chemical Engineering, College of Materials, College of Energy, Xiamen University, Xiamen 361005, China; State Key Laboratory for Physical Chemistry of Solid Surface, College of Chemistry and Chemical Engineering, College of Materials, College of Energy, Xiamen University, Xiamen 361005, China; State Key Laboratory for Physical Chemistry of Solid Surface, College of Chemistry and Chemical Engineering, College of Materials, College of Energy, Xiamen University, Xiamen 361005, China; State Key Laboratory for Physical Chemistry of Solid Surface, College of Chemistry and Chemical Engineering, College of Materials, College of Energy, Xiamen University, Xiamen 361005, China; State Key Laboratory for Physical Chemistry of Solid Surface, College of Chemistry and Chemical Engineering, College of Materials, College of Energy, Xiamen University, Xiamen 361005, China; School of Materials Science and Engineering, ‘The Belt and Road Initiative’ Advanced Materials International Joint Research Center of Hebei Province, Hebei University of Technology, Tianjin 300130, China; State Key Laboratory for Physical Chemistry of Solid Surface, College of Chemistry and Chemical Engineering, College of Materials, College of Energy, Xiamen University, Xiamen 361005, China; State Key Laboratory for Physical Chemistry of Solid Surface, College of Chemistry and Chemical Engineering, College of Materials, College of Energy, Xiamen University, Xiamen 361005, China; State Key Laboratory for Physical Chemistry of Solid Surface, College of Chemistry and Chemical Engineering, College of Materials, College of Energy, Xiamen University, Xiamen 361005, China; State Key Laboratory for Physical Chemistry of Solid Surface, College of Chemistry and Chemical Engineering, College of Materials, College of Energy, Xiamen University, Xiamen 361005, China; State Key Laboratory for Physical Chemistry of Solid Surface, College of Chemistry and Chemical Engineering, College of Materials, College of Energy, Xiamen University, Xiamen 361005, China; State Key Laboratory for Physical Chemistry of Solid Surface, College of Chemistry and Chemical Engineering, College of Materials, College of Energy, Xiamen University, Xiamen 361005, China; Center for High Pressure Science and Technology Advanced Research, Beijing 100193, China; State Key Laboratory for Physical Chemistry of Solid Surface, College of Chemistry and Chemical Engineering, College of Materials, College of Energy, Xiamen University, Xiamen 361005, China; State Key Laboratory for Physical Chemistry of Solid Surface, College of Chemistry and Chemical Engineering, College of Materials, College of Energy, Xiamen University, Xiamen 361005, China; Shenzhen Research Institute of Xiamen University, Shenzhen 518057, China; State Key Laboratory for Physical Chemistry of Solid Surface, College of Chemistry and Chemical Engineering, College of Materials, College of Energy, Xiamen University, Xiamen 361005, China; Innovation Laboratory for Sciences and Technologies of Energy Materials (IKKEM), Xiamen 361102, China

**Keywords:** all-solid-state lithium–sulfur batteries, catalytic mechanisms, high-entropy sulfide catalyst, high-sulfur-content cathodes

## Abstract

Transition-metal sulfide (TMS) catalysis is a promising strategy for accelerating sulfur redox kinetics, yet its catalytic mechanisms in all-solid-state lithium–sulfur batteries (ASSLSBs) remain poorly understood. Here, two distinct catalytic failure modes are identified in conversion-type and intercalation-type TMS catalysts, namely, phase-separation-driven catalyst self-poisoning and transition-metal-migration-induced electrolyte degradation, respectively. Guided by these insights, we develop an all-in-one high-entropy sulfide (HES) catalyst that undergoes a solid-solution reaction to form a stable Li*_x_*HES phase. This Li*_x_*HES functions as both a dual ion-electron conductor, expanding the electrochemically active reaction zone, and an interfacial redox mediator, driving spontaneous Li_2_S oxidation, thereby promoting extensive and rapid S↔Li_2_S conversion. With only 3 wt% HES, the S@HES cathode (40 wt% S) achieves 93.4% sulfur utilization and 93.0% conversion efficiency. Even at 50 wt% sulfur, the cell retains a capacity of 900 mAh g^−1^ at 5 mA cm^−2^ (2C) over 250 cycles. Furthermore, a high areal capacity of 9.68 mAh cm^−2^ is delivered under demanding conditions (8.5 mg cm^−2^, 30°C), highlighting the great promise of the HES catalytic strategy for energy-dense ASSLSBs.

## Teaser

An all-in-one HES catalyst overcomes catalyst self-poisoning and electrolyte degradation, accelerates sulfur conversion and unlocks high-sulfur-content cathodes for energy-dense ASSLSBs.

## INTRODUCTION

The urgent demand for advanced energy storage systems has accelerated the development of post-lithium-ion technologies. Among them, conversion-chemistry-based lithium–sulfur batteries (Li–S) are particularly attractive owing to their exceptionally high theoretical energy density (∼2600 Wh kg^−1^) and the natural abundance of sulfur [1,2]. However, the practical application of liquid-electrolyte-based Li–S batteries has been plagued by the notorious polysulfide shuttle and associated safety concerns [3]. Substituting liquid electrolytes with non-flammable inorganic solid-state electrolytes (SSEs) offers a viable route to fundamentally eliminate the shuttle effect and ensure safety, while additionally enabling bipolar stacking to maximize energy density. Despite these advantages, the realization of high-performance all-solid-state Li–S batteries (ASSLSBs) is still markedly constrained by the sluggish solid-state sulfur redox kinetics. Such kinetic limitations arise primarily from the insulating nature of sulfur species that confines reactions to sparse triple-phase boundaries (TPBs) [4], as well as the high energy barrier for solid-to-solid conversion. More critically, sluggish kinetics induce uncontrolled and inhomogeneous Li_2_S growth, aggravating the intrinsic volume fluctuations and generating localized stress. These effects lead to severe mechanical failure, disruption of the conductive network and ultimately inferior electrochemical performance [5].

To overcome these kinetic issues, extensive efforts have focused on enhancing ionic and electronic conduction throughout the sulfur cathode alongside maximizing active reaction interfaces, which involves strategies such as developing SSEs with superior ionic conductivity [6,7], constructing high-surface-area conductive networks [8,9] and introducing mixed ionic-electronic conductors [10,11]. Although current advances have delivered near-theoretical sulfur capacity, efficient sulfur conversion at high content (>40 wt%) and elevated rates (≥1C) remains challenging (Table S1), severely limiting the practical energy and power densities of ASSLSBs. The high sulfur content in the composite cathode inevitably reduces the proportion of conductive carbon or SSE. In such scenarios, sulfur tends to aggregate, which disrupts ionic and electronic percolation networks and diminishes accessible reaction interfaces [10]. These deficiencies, compounded by the substantial energy barrier for S/Li_2_S transformation, further exacerbate the sluggish redox kinetics [12–14]. Consequently, unlocking high-sulfur-content cathodes necessitates a synergistic strategy that ensures efficient carrier transport and interfacial availability, while simultaneously lowering the reaction energy barrier.

For liquid Li–S systems, the incorporation of catalysts has proven to be highly beneficial for simultaneously enriching reaction interfaces and lowering kinetic barriers [15,16]. Among them, transition-metal sulfides (TMSs; e.g. TiS_2_ [17], MoS_2_ [18], CoS_2_ [19], VS_2_ [20] and LiFeS_2_ [21]) are widely recognized as promising candidates due to their high electronic/ionic conductivity and strong catalytic capability, which can effectively promote sulfur redox kinetics. However, extending such success to ASSLSBs remains far from trivial, as the catalytic processes in liquid systems are predicated on intimate interfacial wetting, a feature inherently absent in solid-state environments. This disparity renders the catalytic mechanisms of TMSs in ASSLSBs largely unknown, particularly regarding solid-state catalytic pathways, catalyst–SSE interfacial compatibility and catalyst failure, which are critical to the rational design of catalysts for ASSLSBs with high-sulfur-content cathodes [22].

In this work, we systematically investigate representative TMS catalysts within the operating voltage range of sulfur conversion, identifying two different failure mechanisms: phase separation in conversion-type FeS_2_ and transition-metal (TM) migration in intercalation-type TiS_2_. These phenomena induce catalyst self-poisoning and electrolyte degradation, thereby fundamentally compromising electrochemical performance. To address these limitations, we propose an all-in-one strategy by rationally designing and synthesizing a pyrite-type high-entropy sulfide (HES) as a catalyst for high-sulfur-content cathodes. Combined experimental and theoretical analyses reveal that HES evolves into a stable Li*_x_*HES phase via a solid-solution reaction. Li*_x_*HES integrates dual ion-electron conductivity with intrinsic Li_2_S-oxidizing capability, functioning as an all-in-one redox mediator to promote sulfur conversion across extensive two-phase interfaces, accelerate redox kinetics and maximize sulfur utilization. As a result, merely 3 wt% of HES catalyst endows the S@HES cathode with excellent rate capability and cycling stability at high sulfur contents (40–50 wt%) and broad mass loadings. Moreover, the catalytic robustness of HES is further validated in a practical full cell paired with a Li_3.75_Si anode, delivering a high gravimetric energy density of 571 Wh kg^−1^ (N/P ratio = 1.6).

## RESULTS AND DISCUSSION

### Failure mechanisms of conventional TMS catalysts

Based on different lithium storage mechanisms, TMSs are generally categorized into conversion-type and intercalation-type materials [23]. Conversion-type TMSs typically deliver high specific capacities through multi-electron redox reactions, albeit at the cost of electrochemical reversibility. In contrast, intercalation-type TMSs operate via single-electron redox processes within a stable anionic framework, enabling fast kinetics but limited capacities (<250 mAh g^−1^) (Fig. S1). To evaluate the catalytic potential of TMSs in ASSLSBs, we investigated the intrinsic electrochemical behaviors of prototypical conversion-type FeS_2_ and intercalation-type TiS_2_ cathodes within the typical voltage window of 1.0–3.0 V (potentials vs. Li^+^/Li unless otherwise stated) [10,24]. As shown in Fig. 1a, and Fig. S2a and b, both cells experience severe capacity decay during the initial five discharge–charge cycles and exhibit severe polarization when returning to 0.1C after high-rate cycling at 1C. The origins of their performance degradation were subsequently probed using differential capacity (d*Q*/d*V*) analysis. For the cell with FeS_2_ cathode, the discrete and intense redox peaks confined to two narrow voltage regions (Fig. S2c) suggest a conversion-dominated mechanism involving the transformation of FeS_2_ into FeS, metallic Fe and insulating Li_2_S phases. This mechanism is also supported by X-ray diffraction (XRD) results, which reveal Li_2_S diffraction peaks in the FeS_2_ cathode upon the first discharge to 1.3 V (Fig. 1b), in agreement with previous reports [25]. Focused ion beam-scanning electron microscopy (FIB-SEM) imaging further visualizes a pronounced spatial separation of these conversion-derived phases, showing a core-shell morphology with an insulating sulfur-rich shell surrounding an Fe-rich core (Fig. 1c). It is worth noting that such phase separation occurs in both micron- and nanosized FeS_2_ particles after conversion (Fig. S3), severely undermining electrode structure and inducing internal cracking as well as loss of interfacial contact with the SSEs. Accordingly, a marked increase in resistance develops during the discharge of the FeS_2_ cell, along with irreversible features after the first charge, as revealed by *in situ* electrochemical impedance spectroscopy (EIS) (Fig. S4a and c). These results collectively explain the high initial capacity yet compromised reversibility of the FeS_2_-based cell. Given the substantial overlap in operating voltages with sulfur cathodes, FeS_2_ inevitably undergoes deep conversion, leading to structural degradation despite its intended catalytic role. We therefore classify this failure mode as catalyst self-poisoning. In this mode, TMS catalysts are irreversibly deactivated, transforming into spatially separated ionic and electronic conducting phases that generate localized reaction hot spots (see path 1 in Fig. 1e), which induce uneven Li_2_S accumulation and severe polarization, ultimately accelerating the performance decline of ASSLSBs.

**Figure 1.**
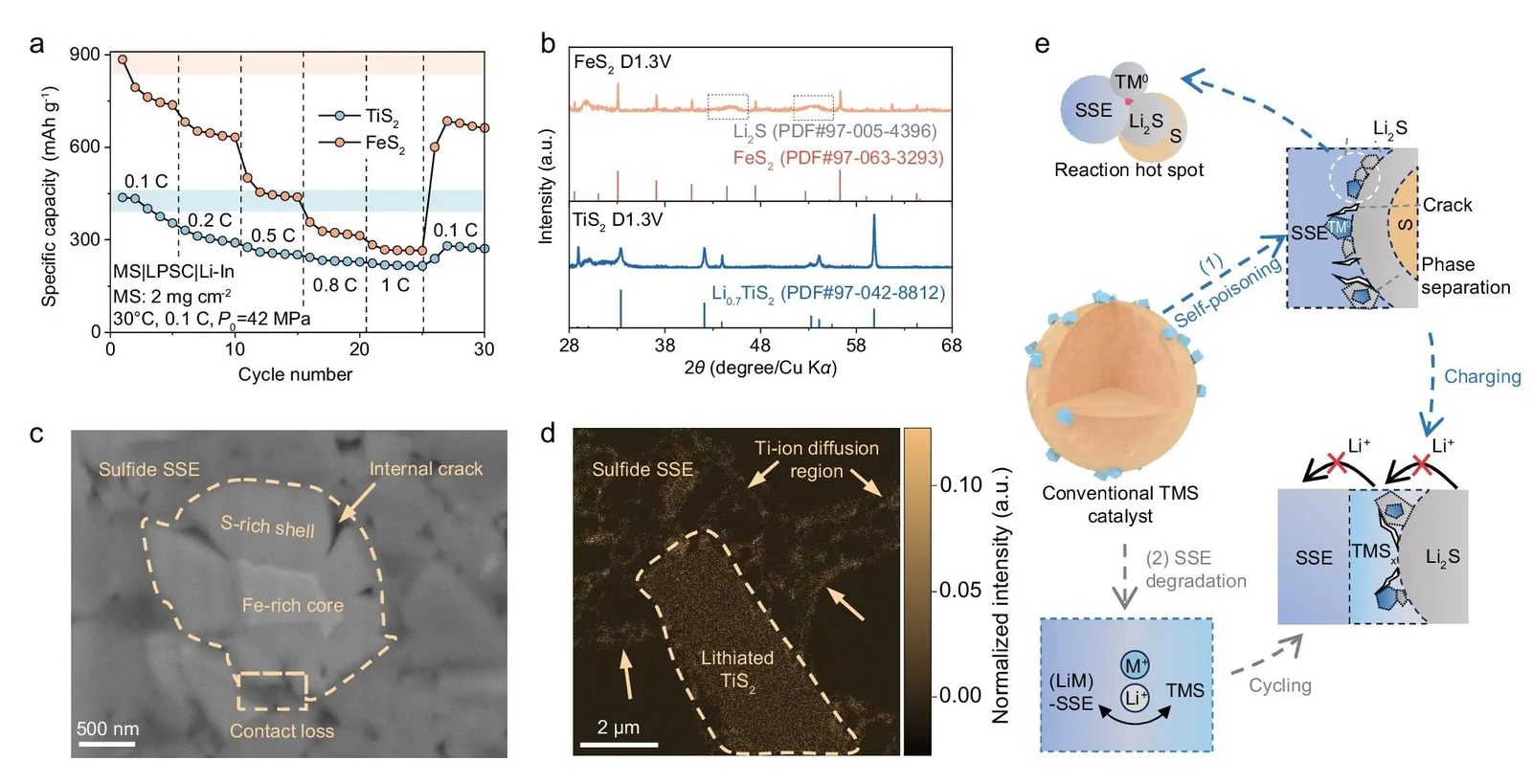
Failure mechanisms of conventional TMS catalysts. (a) Rate performance of cells with FeS_2_ and TiS_2_ cathodes. (b) XRD patterns of TiS_2_ and FeS_2_ cathodes at the discharged state (1.3 V). (c) FIB-SEM image of an FeS_2_ particle after discharging to 1.3 V. (d) Spatial distribution of Ti⁺ fragments (*m*/*z* = 48) obtained by ToF-SIMS in the discharged TiS_2_ cathode. (e) Schematic illustration of the representative failure mechanisms for conventional conversion-type and intercalation-type TMS catalysts.

Unlike the FeS_2_-based cell, reversible lithium insertion/extraction through a solid-solution mechanism is observed in the cell with the intercalation-type TiS_2_ cathode, as evidenced by the broad and stable d*Q*/d*V* features (Fig. S5) at a discharge cutoff voltage of 1.6 V. However, further potential reduction triggers a highly polarized and irreversible decomposition reaction in the TiS_2_-based cell (Fig. S2d), consistent with earlier studies [26,27]. Although only a single lithiated phase is detected in XRD patterns collected at 1.3 V (Fig. 1b), time-of-flight secondary ion mass spectrometry (ToF-SIMS) imaging reveals the migration of Ti species into the SSEs during cycling (Fig. 1d). Such TM migration may locally increase the electronic conductivity of the SSE, but typically triggers coupled electrochemical-mechanical degradation of the SSE matrix (see section entitled ‘S1’ of the Supplementary data). The progressive loss of TM ions, accompanied by electrolyte decomposition, leads to a significant increase in interfacial resistance within the TiS_2_-based cell (Fig. S4b and d). Accordingly, we attribute the failure of intercalation-type TMSs in solid-state sulfur catalysis primarily to electrolyte degradation (see path 2 in Fig. 1e).

### Rational design and mechanical–electrochemical properties of HES catalysts

The catalyst self-poisoning and electrolyte degradation discussed above shed light on the intrinsic limitations of binary TMSs in ASSLSBs, and also highlight the necessity of preventing phase separation and TM migration during sulfur conversion to preserve structural integrity for effective solid-state sulfur catalysis. Motivated by these insights, we propose an all-in-one high-entropy strategy for TMSs, as illustrated in Fig. 2a. In principle, high configurational entropy tends to improve the thermodynamic stability of the solid-solution phase against phase separation, while sluggish diffusion helps to kinetically hinder long-range TM migration, both of which are beneficial for maintaining a structurally stable framework with continuous ionic and electronic transport during cycling [28]. Moreover, the high-entropy framework is expected to impart enhanced chemomechanical robustness to accommodate the strain induced by sulfur volume fluctuations, ensuring durable interfacial contact [29]. Beyond structural stability, the chemically diverse local environment is capable of improving surface sulfiphilicity and regulating the electronic structure, thereby facilitating sulfur adsorption, activation and interfacial charge transfer [30]. Together, these effects are believed to enable faster solid-state sulfur conversion.

**Figure 2.**
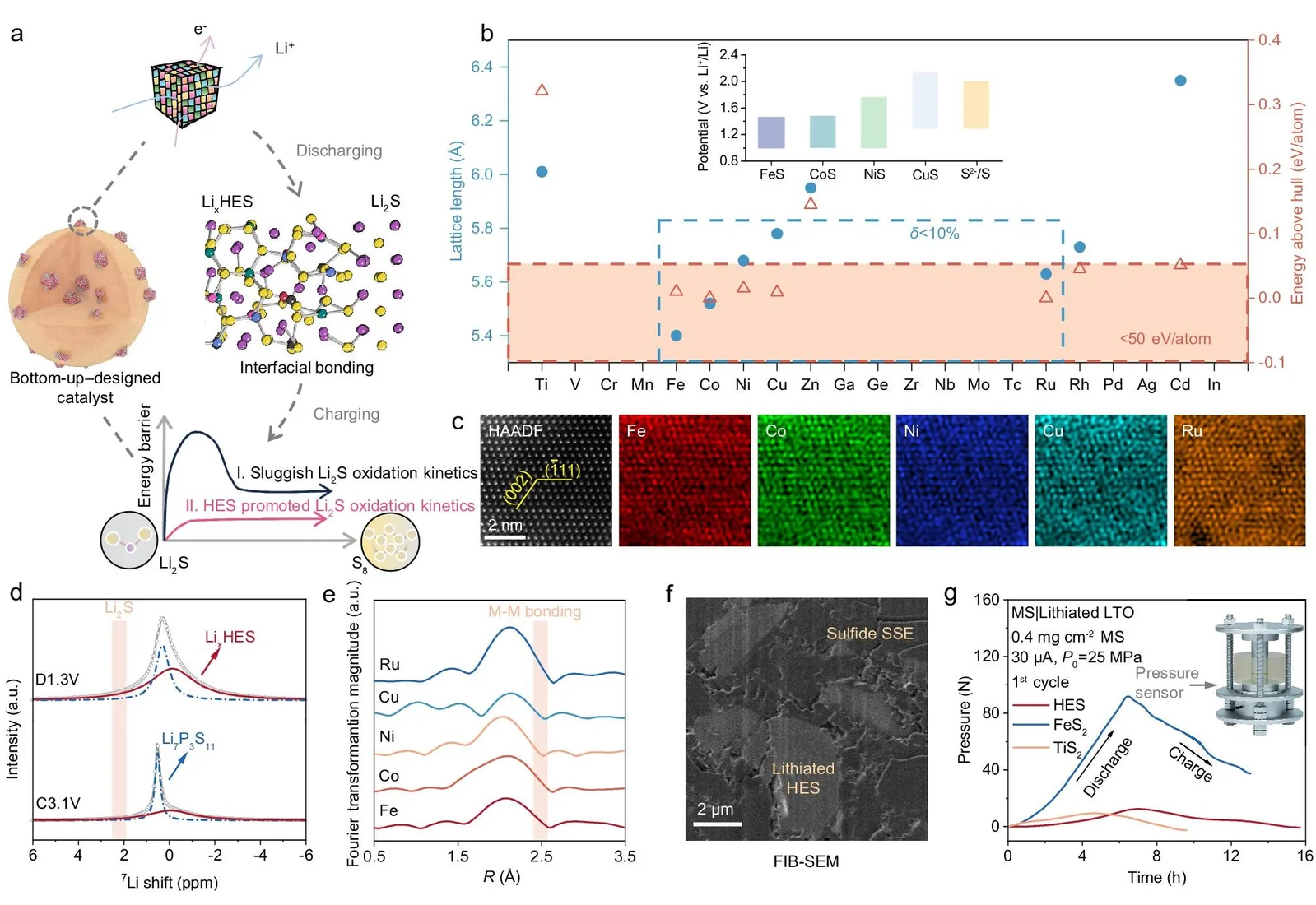
Rational design and mechanical–electrochemical properties of HES catalysts. (a) Bottom-up catalyst design concept for solid-state sulfur conversion. (b) Elemental screening criteria for high-entropy pyrite-type sulfides, including formation energies (<50 meV atom^−1^ [38]) and lattice mismatch with the FeS_2_ host, defined as $\delta \ {\mathrm{ = }}\frac{{{\mathrm{|}}{a}_{{\mathrm{dopant}}\ }\ {\mathrm{ - \ }}{a}_{{\mathrm{Fe}}{{\mathrm{S}}}_{\mathrm{2}}}{\mathrm{|}}}}{{{a}_{{\mathrm{Fe}}{{\mathrm{S}}}_{\mathrm{2}}}}}{\mathrm{\ \times \ 100\% }}$<10% [39]. The inset shows redox potential ranges of binary TMS for the selected constituent elements (Fe, Co, Ni and Cu). (c) Atomic-resolution HAADF-STEM image of HES with the corresponding EDS elemental mappings of Fe, Co, Ni, Cu and Ru. (d) ^7^Li ssNMR spectra of HES cathodes at discharged (1.3 V) and charged (3.1 V) states. (e) Fourier-transformed EXAFS spectra of Co, Cu, Ru, Fe and Ni for the HES cathode after discharge at 0.1C. (f) FIB-SEM image of the discharged HES cathode. (g) Comparison of operando pressure evolution in cells with HES, FeS_2_ and TiS_2_ cathodes.

To implement this strategy, we selected the pyrite-type lattice as the structural prototype, due to its compositional flexibility and intrinsic catalytic capability [31]. A systematic screening of binary pyrite-type TMSs was conducted using the Materials Project database to identify candidate HESs. The key selection criteria include (i) thermodynamic formability, defined by formation energies within 50 meV atom^−1^ above the convex hull to ensure synthetic feasibility [32], and (ii) lattice compatibility, quantified by a lattice mismatch parameter (*δ*) that guarantees the formation of a single-phase solid solution. By employing this screening protocol, a quinary (Fe, Co, Ni, Cu, Ru)S_2_ system was determined to be a suitable formulation (Fig. 2b). Within this multicomponent framework, the constituent elements play distinct yet complementary roles. Specifically, Fe, Co, Ni and Cu serve as redox-active centers, providing a continuous reduction voltage range (1.3–2.15 V, experimentally evidenced by the d*Q*/d*V* profiles in Fig. S6). This range overlaps well with the voltage window for sulfur reduction (1.3–2.0 V, inset in Fig. 2b), which favors efficient charge transfer. Although Ru remains electrochemically inactive within this voltage range [33], its higher valence state and stronger metal–sulfur bond render it a critical structural anchor, reinforcing the rigidity of the high-entropy framework.

Guided by the aforementioned bottom-up design rationale, the quinary Fe-Co-Ni-Cu-Ru HES was synthesized via high-energy ball milling of the constituent metal powders and elemental sulfur, followed by a high-temperature, high-pressure treatment [34]. Notably, the redox-active centers (Fe, Co, Ni and Cu) were weighed in a near-equimolar ratio, while the Ru content was intentionally reduced, as Ru is redox-inactive under the operating conditions and primarily serves as a structural stabilizer. SEM images in Fig. S7 show that the as-synthesized product has an irregular morphology, with the particle sizes readily controlled by post-ball milling. XRD analysis confirms the formation of a high-purity cubic pyrite-type HES phase (space group $Pa{\mathrm{\bar{3}}}$), as evidenced by the complete absence of impurity peaks (Fig. S8). Furthermore, compared with the constituent binary sulfides, the diffraction patterns exhibit a slight peak shift, which indicates successful multi-element integration and the resulting variation in the lattice parameter (Fig. S9). To determine the elemental stoichiometry, inductively coupled plasma optical emission spectroscopy was performed. The result reveals a metal composition of Fe_0.23_Co_0.21_Ni_0.24_Cu_0.2_Ru_0.12_, which closely matches the designed stoichiometry (Table S2). According to this composition, the configurational entropy (Δ*S*_conf_) is calculated to be 1.58R per unit cell, exceeding the empirical threshold for high-entropy materials (1.5R), which therefore substantiates the high-entropy nature of the synthesized HES. The atomic-level structure of HES was further investigated using high-angle annular dark-field (HAADF) imaging in scanning transmission electron microscopy (STEM) mode. As presented in Fig. 2c, the energy-dispersive X-ray spectroscopy (EDS) elemental mappings of Fe, Co, Ni, Cu and Ru present nearly homogeneous atomic-scale distribution without noticeable clustering or segregation. This observation supports the formation of a single-phase solid solution featuring a homogeneously mixed cationic arrangement. Complementing the imaging results, the extended X-ray absorption fine structure (EXAFS) spectra of the HES demonstrate predominant coordination of the constituent metals, with M–S bond lengths mainly distributed around ∼2.2 Å (in R-space) (Fig. S10), suggesting a highly uniform local coordination environment.

After the successful synthesis of HES, we systematically investigated the intrinsic advantages conferred by this high-entropy configuration. Rietveld refinement of the XRD patterns determined a lattice parameter of *a* = 5.609 Å for the HES. The corresponding unit-cell volume is larger than that of FeS_2_, which reflects a distorted lattice caused by the size mismatch of the multiple incorporated cations (Tables S3 and S4). Crucially, this complex cationic incorporation also induces the formation of sulfur vacancies, as confirmed via an electron paramagnetic resonance signal at *g* = 2.01 (Fig. S11). Together, the expanded lattice and sulfur vacancies are anticipated to accelerate Li^+^ ion diffusion and buffer volumetric strain. To assess the strain-buffering capability, single-particle compression tests were conducted. As shown in Fig. S12, the HES shows substantially higher fracture resistance (1018 MPa), with progressive deformation and delayed crack propagation. In comparison, FeS_2_ fractures rapidly breaking into fine debris under much lower applied stress (260 MPa). This improved mechanical robustness allows the HES to accommodate the severe volume expansion of sulfur during conversion, preventing structural collapse.

Along with these physical structural benefits, density functional theory (DFT) calculations were carried out to elucidate the electronic structure evolution induced by the high-entropy configuration. As plotted in Fig. S13a, FeS_2_ has a semiconducting band gap of 0.96 eV, whereas HES presents a non-zero density of states (DOS) at the Fermi level, indicative of a metallic nature. Further projected DOS analysis clarifies that this semiconductor-to-metal transition stems from electronic reconstruction, where the incorporation of diverse TM species introduces additional *d*-orbital states near the Fermi level (Fig. S13b and c). Such a metallic framework promises a markedly accelerated kinetics for charge transfer, thereby driving electrochemical reactions. To experimentally verify these theoretical predictions, *in situ* EIS measurements were performed, which revealed negligible resistance growth during the first cycle in the HES-based cell (Fig. S14a). These findings highlight the multifunctionality of the HES design: its metallic electronic structure provides high electronic conductivity, while its expanded, defect-rich lattice enables rapid ionic diffusion.

Driven by these structural and electronic modifications, the overall lithium-storage mechanism of the HES is fundamentally altered. As evidenced in Fig. S14b, the HES cell displays a continuous and sloping voltage profile, in sharp contrast to the distinct plateaus of the conversion reaction, consistent with a solid-solution mechanism rather than a discrete two-phase process. This mechanism is also corroborated by *in situ* XRD, showing that the pristine HES diffraction peaks gradually attenuate during discharge without the appearance of new peaks (Fig. S15a and b). Notably, even upon deep discharge to 1.3 V, crystalline Li_2_S, the typical conversion product of TMS, remains undetectable in XRD patterns of the discharged HES (Fig. S15c). The absence of detectable Li_2_S reflections suggests that the discharge products are predominantly amorphous or nanocrystalline, falling below the detection limit of XRD. To further determine whether Li_2_S was formed, solid-state nuclear magnetic resonance (ssNMR) was employed. Specifically, a T_2_-filtering sequence was implemented to separate the overlapping signals. By selectively suppressing the short-T_2_  ^7^Li signal while retaining the longer-T_2_ component, the filter enables reliable spectral discrimination. As presented in Fig. 2d and Fig. S16, ^7^Li ssNMR with T_2_ filtering clearly distinguishes the Li^+^ ion signals at −0.12 ppm in the HES lattice and 0.45 ppm in the solid Li_7_P_3_S_11_ (LPS) [35]. Crucially, the absence of Li_2_S resonance (∼2.5 ppm [36]) after discharging HES to 1.3 V rules out a conversion reaction and instead supports a solid-solution process to form the Li*_x_*HES phase. Upon recharging to 3.1 V, the intensity of the Li*_x_*HES signal decreases significantly, indicating substantial and reversible Li^+^ ion extraction. Moreover, EXAFS analysis reveals that the local metal–sulfur coordination environment in HES remains largely preserved during cycling, with no signal of extensive bond rearrangement or metallic clustering (Fig. 2e and Fig. S17). Collectively, these measurements substantiate that HES operates via a robust solid-solution pathway, effectively circumventing the detrimental phase separation and large-scale atomic reconstruction typically seen in conversion reactions.

At the macroscopic electrode level, a direct consequence of this non-destructive solid-solution mechanism is its capability to significantly mitigate volume expansion during cycling. As expected, the operando electrochemical pressure measurements demonstrate minimal pressure fluctuations and excellent mechanical reversibility in the HES cell (Fig. 2g and Fig. S18). Remarkably, the HES cell exhibits a pressure increase rate of only 1.8 N h^−1^ during the first discharge, comparable to that of the intercalation-type TiS_2_ cell (2.0 N h^−1^) but an order of magnitude lower than that of the conversion-type FeS_2_ cell (14.3 N h^−1^). Such alleviated chemomechanical strain ultimately helps to maintain the morphological and compositional integrity of the electrodes. This is visually demonstrated by cross-sectional FIB-SEM imaging, which shows intact particles without phase separation after full discharge (Fig. 2f). Furthermore, ToF-SIMS mapping (Fig. S19) verifies the chemical uniformity at a microscopic level without TM migration.

Benefiting from the synergy of fast kinetics, a favorable solid-solution reaction mechanism and robust mechanical toughness, the cell with a micron-sized HES cathode maintains stable cycling across all tested rates, and most notably, it preserves the excellent cyclability upon returning to 0.1C after prolonged operation at 1C (Fig. S20). According to the above results, we believe that HES holds considerable potential as a catalyst for extending the operation of ASSLSBs.

### Electrochemical performance of ASSLSBs with S@HES cathodes

To validate the catalytic role of HES in improving the electrochemical performance of ASSLSBs, composite cathodes were prepared by high-energy ball milling of sulfur, HES, carbon and LPS, with all cathodes containing 40 wt% sulfur unless otherwise stated. XRD patterns and electron microscopy images indicate that HES retains its crystallinity (Fig. S21) and is uniformly dispersed throughout the sulfur matrix (Fig. S22), showing intimate contact with sulfur, carbon and SSE. Note that, rather than introducing HES merely as an additional additive, we isolated its electrochemical contribution by partially substituting the conductive carbon (acetylene black, AB) for HES within the composite cathode. This substitution establishes a rigorous test to determine whether HES can simultaneously function as an efficient electronic conductor and a potent catalyst for sulfur conversion. Through optimization of the HES ratio, the composite cathode with a 3 wt% catalyst (hereafter denoted as S@HES) yields the highest initial Coulombic efficiency (ICE = 92%) and superior rate performance (Fig. S23). Moreover, the S@HES cell demonstrates markedly improved cycling stability compared to the carbon-only baseline (S@AB) cell under high-rate conditions. Specifically, the S@HES cell achieves a high discharge capacity of 892 mAh g^−1^ with 73% capacity retention over 1000 cycles at 1C, whereas the control S@AB counterpart delivers an initial discharge capacity of only ∼518 mAh g^−1^ and suffers rapid capacity decay after ∼125 cycles (Fig. 3a). In addition to maintaining high specific capacity, the S@HES cell also exhibits substantially reduced voltage hysteresis, with a narrower charge–discharge gap at 400 mAh g^−1^ (0.96 V vs. 1.36 V; Fig. S24), providing evidence of accelerated sulfur redox kinetics.

**Figure 3.**
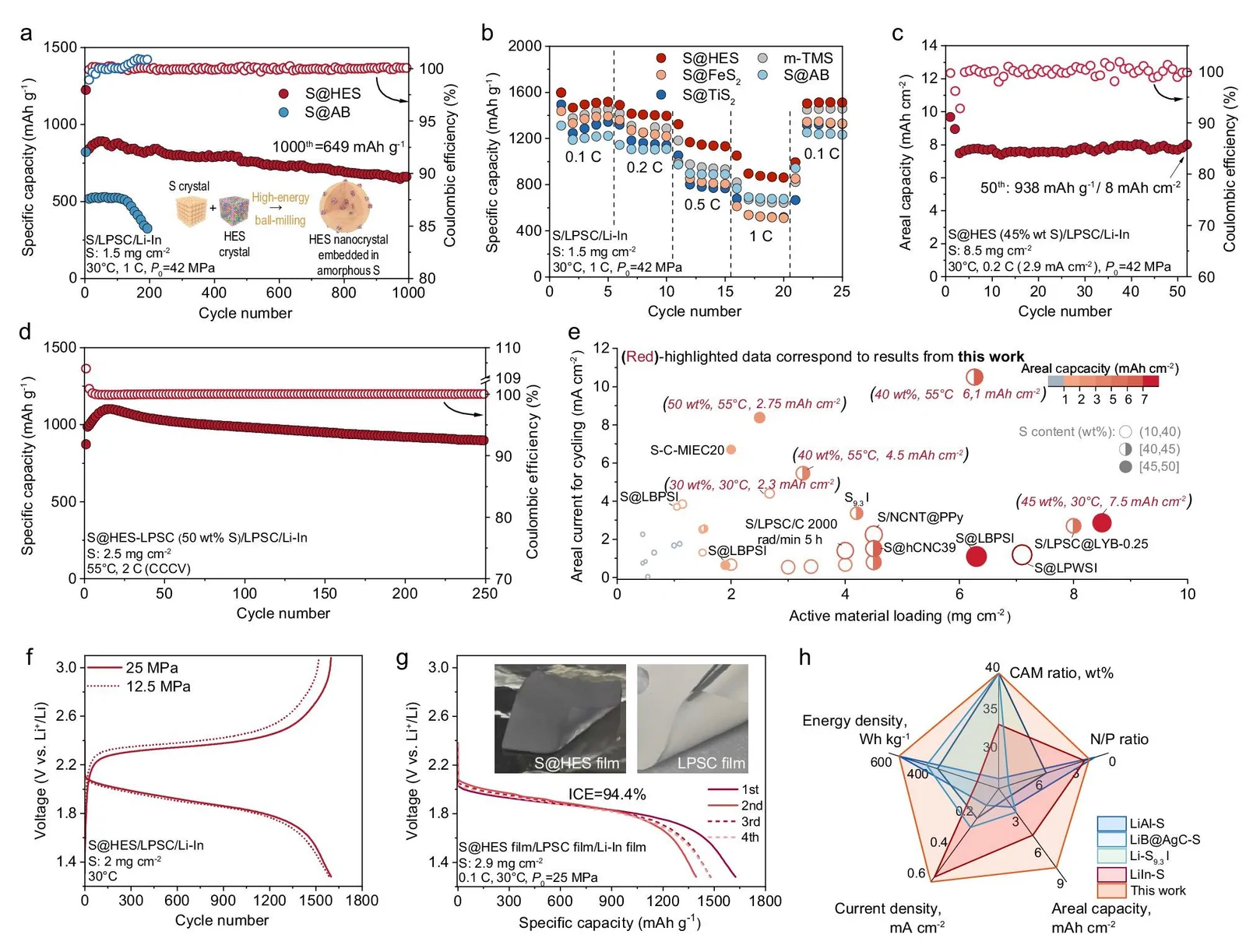
Electrochemical performance of ASSLSBs with S@HES cathodes. (a) Cycling performance of cells with S@HES and S@AB cathodes at 1C. (b) Rate performance comparison of cells employing S@HES, S@FeS_2_, S@TiS_2_, S@m-TMS and S@AB cathodes. (c) Cycling performance of S@HES cells with a high sulfur loading of 8.5 mg cm^−2^ at 0.2C. (d) Cycling performance of S@HES cell using LPSC as the electrolyte within the composite cathode at 2C (constant-current-constant-voltage (CC-CV) mode, cutoff current of 0.1C at 3.1 V). (e) Performance comparison between half-cells in this work and other studies. Detailed data are provided in Table S1. (f) Initial discharge–charge profiles of S@HES cells at 0.1C under stack pressures of 12.5 and 25 MPa. (g) Discharge voltage profiles of S@HES cells assembled using dry-processed electrodes. (h) Radar plot comparison of all-solid-state Li–S full cells reported previously (LiAl–S [41], LiB@AgC–S [42], Li–S_9.3_I [43] and LiIn–S [37]) and the S@HES|LPSC|Li_3.75_Si full cell demonstrated in this work.

To accurately assess sulfur utilization, we performed control experiments using sulfur-free electrodes. As shown in Fig. S25a, HES contributes a negligible capacity (∼12 mAh g^−1^, normalized to sulfur mass, at 0.1C). Moreover, its incorporation does not measurably increase the capacity contribution arising from LPS decomposition, as evidenced by the nearly identical reversible capacities of LPS-AB-HES and LPS-AB electrodes. Consistently, X-ray photoelectron spectroscopy reveals that the characteristic LPS-related P=S, P–S–Li and P–S–P species remain largely preserved after cycling (Fig. S26). After subtracting the HES capacity contribution, the sulfur component in the S@HES cell provides an initial discharge capacity of 1564 mAh g^−1^ and a charge capacity of 1455 mAh g^−1^ (Fig. S25b). This corresponds to a sulfur utilization of 93.4% (relative to theoretical 1675 mAh g^−1^) and a sulfur conversion efficiency of 93.0%, which are superior to the 89% utilization and 83% efficiency observed in the S@AB cell. The performance gap becomes even more pronounced at 1C, where the initial discharge capacity still reaches 890 mAh g^−1^ in the S@HES cell, which is 1.7 times higher than that of the S@AB cell. Notably, this superior performance is unique to the HES. When HES was replaced with FeS_2_, TiS_2_ or a mechanically mixed TMS composite (FeS_2_, CoS_2_, NiS_2_ and CuS; denoted as m-TMS at identical loadings), all three control cathodes exhibited inferior sulfur utilization and rate capability (Fig. 3b). In particular, the m-TMS cathode failed to match the capacity of S@HES at 1C, indicating that the catalytic advantage of HES arises from its homogeneous single-phase architecture rather than a simple physical mixture of its constituent binary sulfides.

The robustness of HES-assisted sulfur conversion was further confirmed across a broad parameter space, covering sulfur loading, sulfur content and C-rate. In terms of sulfur loading, the S@HES cell retains stable cycling at 1C with a sulfur loading of 3.3 mg cm^−2^, affording an areal capacity of 3.23 mAh cm^−2^ (∼979 mAh g^−1^) after 400 cycles (Fig. S27a). When the sulfur loading was increased to 6.3 mg cm^−2^, the cell sustained a high areal capacity of 6.1 mAh cm^−2^ at 1C (Fig. S27b). Considering the critical dependence of performance on the active material fraction, the S@HES cell with different sulfur contents was also evaluated. With 30 wt% sulfur, the cells maintain stable cycling at 2C over 300 cycles under ambient conditions, delivering discharge capacities of 1416 and 677 mAh g^−1^ at sulfur loadings of 1.2 and 2.7 mg cm^−2^, respectively (Fig. S28). Moreover, when the sulfur content is increased to 50 wt%, a stable discharge capacity of 600 mAh g^−1^ is still retained at 2C after 300 cycles (Fig. S29). More impressively, even under demanding electrode design conditions with a high loading of 8.5 mg cm^−2^ and a high content of 45 wt%, the S@HES cell yields remarkable areal capacities of 9.68 mAh cm^−2^ at 0.05C and 7.49 mAh cm^−2^ at 0.2C (Fig. 3c), preserving ∼8 mAh cm^−2^ after 50 cycles. To further maximize the potential of the S@HES cathode, we optimized the solid-state environment by substituting the LPS electrolyte with higher-conductivity Li_6_PS_5_Cl (LPSC). The resulting S@HES-LPSC cell retains a capacity of 900 mAh g^−1^ after 250 cycles at 2C (Fig. 3d). Compared with state-of-the-art half cells, the S@HES cell ranks among the leading half-cells reported to date (Fig. 3e and Fig. S30). Overall, the exceptional electrochemical performance under demanding practical parameters highlights the great promise of the HES catalyst for next-generation ASSLSBs.

Despite promising half-cell metrics, practical implementation of ASSLSBs faces stringent engineering challenges, especially the need for high stacking pressure and complex manufacturing. To this end, we evaluated the electrochemical performance of the S@HES cell under a lower stacking pressure. As shown in Fig. 3f, the S@HES cell delivers a remarkable initial discharge capacity of 1517 mAh g^−1^ at a reduced pressure of 12.5 MPa, close to 1598 mAh g^−1^ at 25 MPa, and exhibits a capacity retention of 95.6% over 30 cycles (Fig. S31). This pressure-tolerant behavior indicates that the HES catalyst facilitates robust interfacial contact even under lower mechanical constraints, alleviating a major bottleneck for ASSLSBs. To address manufacturing scalability, dry-processed S@HES cathodes were fabricated. When paired with a Li–In film anode, a high initial discharge capacity of 1626 mAh g^−1^ and an outstanding ICE of 94.4% are achieved in the resulting cell (Fig. 3g). These values are highly comparable to those of conventionally processed counterparts, highlighting the excellent compatibility of S@HES with scalable manufacturing processes.

Next, we assembled full cells using the dry-processed S@HES cathode and a more practical low-potential Li_3.75_Si anode. Since the low operating potential of Li_3.75_Si is expected to induce the reductive decomposition of polytetrafluoroethylene (PTFE), a bilayer LPSC film was employed. The cathode-side layer contained 1 wt% PTFE to ensure film robustness, whereas the anode-side layer contained 0.2 wt% PTFE to improve reductive stability toward Li_3.75_Si. The resulting dry-film S@HES||Li_3.75_Si cell exhibits a second-cycle discharge capacity of 1148 mAh g^−1^, and gradually stabilizes at 1582 mAh g^−1^ after 10 cycles (Fig. S32).

Having established both dry-process compatibility and low-potential anode compatibility, the energy densities of S@HES||Li_3.75_Si full cells with a controlled negative-to-positive (N/P) ratio were further evaluated. Remarkably, at an N/P ratio of 1.6, the cell exhibits a gravimetric energy density of 571 Wh kg^−1^ (based on total electrode mass) at 0.25 mA cm^−2^ with a 3.3 mg cm^−2^ sulfur loading. It still reaches 545 Wh kg^−1^ even at a higher loading of 5.0 mg cm^−2^ and current density of 0.6 mA cm^−2^ (Fig. 3h and Fig. S33). These outstanding performance metrics position the S@HES full cell among the most competitive ASSLSBs reported to date (Table S5), compellingly demonstrating that the HES-assisted sulfur conversion strategy successfully bridges the gap between fundamental kinetic enhancement and application-relevant solid-state battery operation.

### Origin of superior electrochemical performance

To elucidate the origin of the superior electrochemical performance, d*Q*/d*V* analysis was conducted to probe the redox behavior of the S@HES and S@AB cathodes. As shown in Fig. 4a, two well-defined cathodic peaks at ∼1.92 and ∼1.84 V (labeled as peaks i and ii) are detected in the S@AB cathode, corresponding to the stepwise reduction of sulfur to Li_2_S_2_ intermediates and finally to Li_2_S, consistent with previous studies. In contrast, the cathodic peaks in the S@HES cathode present an ∼36 mV positive shift and increased intensity, reflecting reduced polarization and enhanced sulfur utilization. These combined advantages are also observed in the subsequent charging process. Specifically, compared to the broad anodic peak at ∼2.40 V (peak iv) in the S@AB cathode, the anodic peak of the S@HES cathode becomes sharper and more intense at a slightly lower potential, indicating faster and more thorough oxidation of Li_2_S back to sulfur. We attribute this notable difference in the S@HES cathode to the redox-activated catalytic mechanism of HES. Crucially, the catalytic activity of HES is unlocked by a redox reaction that precedes sulfur reduction and Li_2_S oxidation, as evidenced by the cathodic peak at ∼2.06 V (peak iii). During this process, HES transitions via a solid-solution pathway, forming a dual-carrier conducting (DCC) Li*_x_*HES phase that provides robust active sites for subsequent sulfur conversion. Consequently, the sulfur redox reaction zone expands from the limited TPBs to abundant S/Li_2_S|Li*_x_*HES interfaces, thereby facilitating rapid redox kinetics and more complete sulfur utilization. The unique catalytic advantage of HES is further supported by the d*Q*/d*V* analysis of S@FeS_2_ and S@TiS_2_ (Fig. S34). Although S@FeS_2_ also exhibits an initial lithiation feature, it provides little relief of the subsequent cathodic polarization, indicating limited catalytic promotion for sulfur reduction. This limitation can be explained by the intrinsic conversion-type degradation of FeS_2_ discussed above, including irreversible phase separation, particle cracking and loss of interfacial contact, all of which progressively diminish the effective catalytic interface. For S@TiS_2_, the sulfur reduction peaks become increasingly polarized relative to S@AB, accompanied by continuously weakened anodic peaks upon cycling, indicating sluggish sulfur reduction and deteriorating Li_2_S oxidation. These effects are likely driven by Ti migration and SSE degradation, which disrupt the catalytic interface and increase transport resistance.

**Figure 4.**
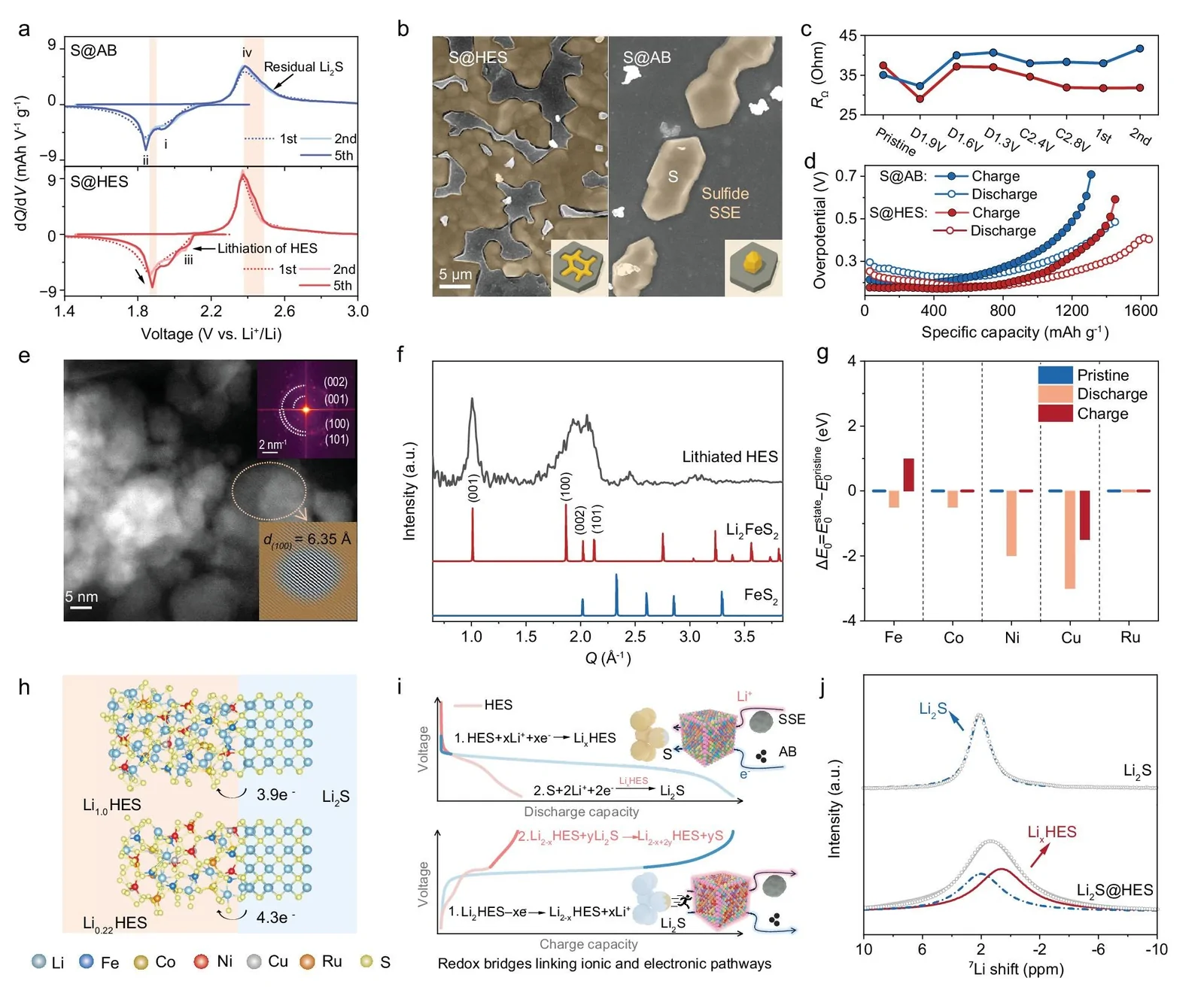
Mechanistic origin of enhanced sulfur conversion kinetics enabled by HES. (a) d*Q*/d*V* curves of the S@AB and S@HES cathodes at certain cycles. (b) Morphological comparison of S@HES and S@AB cathodes after the CC-CV. (c and d) Evolution of *R*_Ω_ (c) and quasi-equilibrium overpotential (d) of ASSLSBs with S@HES and S@AB at different discharged and charged states during the first cycle. (e) HR-STEM image of HES after discharge to 1.3 V (vs. Li^+^/Li) at 0.1 C. (f) Radial intensity profiles extracted from the corresponding fast FFT patterns derived in panel (e). *Q* is the scattering vector calculated by 2π/*d*, where *d* is the interplanar spacing. (g) K-edge energy shifts (Δ*E*_0_) of Fe, Co, Ni, Cu and Ru in HES at discharged and charged states relative to the pristine state. (h) Relaxed heterojunction structures with corresponding Bader charge distributions for Li_2_S|Li_1.0_HES (upper) and Li_2_S|Li_0.22_HES (lower); pristine heterojunctions are shown in Fig. S45. (i) Schematic illustration of the proposed kinetic enhancement mechanism, highlighting two synergistic effects: (1) stable ionic-electronic dual-carrier transport and (2) strong sulfur affinity coupled with reversible redox mediation. (j) ^7^Li ssNMR spectra of Li_2_S physically mixed with HES.

Sulfur K-edge X-ray absorption near-edge structure (XANES) spectra further provide direct chemical evidence for the efficient sulfur conversion enabled by the redox-activated catalysis in the S@HES cathode. As plotted in Fig. S35, while the fully discharged S@HES cathode displays a slightly stronger Li_2_S-related peak intensity than the S@AB cathode, the sulfur-related signal in the S@HES cathode upon full charging intensifies significantly, with its peak center closely matching that of pristine sulfur. This spectral recovery explicitly indicates the more complete Li_2_S oxidation in the S@HES cathode, which is essential for achieving high-rate capability and stable cycling in ASSLSBs, as the oxidative decomposition of insulating Li_2_S is widely regarded as the rate-limiting step [6]. The enhanced degree of Li_2_S oxidation was further visually confirmed by top-view SEM imaging (Fig. 4b), which exhibits a more uniformly dispersed crystalline sulfur in the S@HES cathode after full charge. Consistent with these observations, the corresponding discharge–charge profiles reveal that the S@HES cell achieves over twice the sulfur utilization of the S@AB cell under a high areal loading of 4.5 mg cm^−2^ at 1C (Fig. S36).

To complement the chemical and electrochemical analyses, we quantified the catalytic acceleration effect for sulfur redox kinetics via *in situ* EIS and galvanostatic intermittent titration technique (GITT) measurements. Before cycling, the Nyquist plots for both cells show comparable ohmic resistance (*R*_Ω_), meaning similar carrier transport properties (Fig. S37). However, the evolution of *R*_Ω_ differs significantly upon cycling (Fig. 4c). The S@AB cell suffers from an upward trend in *R*_Ω_, which is caused by the progressive blockage of electronic and ionic transport pathways by the accumulation of insulating Li_2_S. Conversely, *R*_Ω_ remains consistently low in the S@HES cell, particularly at the end of charging, corresponding to the formation of a DCC Li*_x_*HES matrix and the more complete Li_2_S oxidation. The suppression of *R*_Ω_ growth ensures continuous carrier transport throughout cycling, thereby promoting efficient sulfur redox reactions and improving both rate capability and long-term cycling stability of the cell. GITT results further confirm the kinetic advantage of the S@HES cell, with the Li^+^ ion diffusion coefficient gradually increasing to 2.5 × 10^−12^ cm^2^ s^−1^ at the end of charge, approximately one order of magnitude higher than in the S@AB cell (6.1 × 10^−13^ cm^2^ s^−1^) (Fig. S38). Moreover, the quasi-equilibrium overpotentials of the S@HES cell are markedly reduced by 10 mV at the end of discharge and charge, relative to the S@AB cell (Fig. 4d), indicating substantially lower electrochemical polarization, which agrees well with the d*Q*/d*V* analysis.

Having established the redox-activated catalytic mechanism as the key to the enhanced sulfur cathode performance, we next employed high-resolution transmission electron microscopy and high-resolution STEM (HR-STEM) to directly visualize the structural evolution underlying the redox activation of HES. As depicted in Fig. 4e and Fig. S39, the fully lithiated HES particles retain a uniform size distribution (<10 nm), comparable to their pristine state (Fig. S22), highlighting their exceptional structural robustness. Notably, clear lattice fringes are observed throughout the domains, suggesting that fully lithiated HES maintains a well-ordered state rather than becoming amorphous. To precisely identify this highly crystalline lithiated phase, radial intensity profiles were extracted from the corresponding fast Fourier transform (FFT) patterns. The diffraction data of fully lithiated HES align well with the simulated pattern of the lithiated pyrite FeS_2_ (i.e. the Li_2_FeS_2_ phase) (Fig. 4f). This strong crystallographic correspondence implies that the lithiation mechanism of HES closely parallels the commonly accepted conversion of pyrite FeS_2_ to Li_2_FeS_2_, a metastable phase formed electrochemically and accompanied by a symmetry transition from cubic to trigonal (space group: $P{\mathrm{\bar{3}}}m{\mathrm{1}}$) [40]. Ultimately, it is this robust, highly crystalline Li_2_HES phase that provides the durable DCC network and active sites for sulfur conversion. Based on DFT calculations, the potential structural evolution from HES to Li_2_HES, followed by irreversible delithiation to Li_0.22_HES, is schematically shown in Fig. S40, which includes the energetically stable Li_1.0_HES intermediate phase. The structural robustness of Li_2_HES is further supported by thermodynamic calculations, and the Gibbs free energy change for the Li_2_HES decomposition reaction is calculated to be 53.39 kJ mol^−1^ (Table S6), rendering such a pathway thermodynamically unfavorable. In addition to structural stability, the DFT calculations reveal that the DOS of Li*_x_*HES (*x* = 2, 1, 0.22) exhibits a high carrier density at the Fermi level (Fig. S41). This suggests that these lithiated phases retain high electrical conductivity, which underpins the enhanced kinetics observed in *in situ* EIS and GITT.

To gain a deeper insight into the redox activation within the HES lattice, XANES measurements were performed at the K-edges of all five constituent TMs (Fe, Co, Ni, Cu and Ru) to monitor their individual redox contributions during cycling. As illustrated in Fig. 4g and Fig. S42, the K-edges of Fe, Co, Ni and Cu shift simultaneously to lower energies during discharge and largely recover upon charging, confirming their reversible redox activities. Among them, the Cu K-edge exhibits the most pronounced energy shifts (∼3 eV), consistent with partial reduction toward a Cu^+^ state, reflecting its role as the primary redox-active center. Notably, the Cu K-edge also shows incomplete recovery upon charging, whereas the Fe K-edge shifts to a higher energy than in the pristine state, indicating an asymmetric valence redistribution among the TMs. In contrast, the Ru K-edge remains essentially unchanged throughout the cycle, indicating a highly stable oxidation state. To verify the stabilizing role of Ru, a Ru-free HES was synthesized as a control. Unlike the Ru-containing system, this counterpart exhibits pronounced electrochemical instability (Fig. S43a), including rapid voltage decay and a two-step oxidation profile resembling that of FeS_2_ (Fig. S43b). *Ex situ* XRD reveals the formation of Li_2_S after lithiation (Fig. S43c), indicating that the absence of Ru drives the HES toward a conversion-like reaction pathway similar to that of the FeS_2_ cathode. To further understand the structural stabilization provided by Ru, bond strengths across various metal–sulfur pairs were quantified via minus Integrated Crystal Orbital Hamilton Populations. The results identify the Ru–S bond as the most robust bonding interaction within the lattice across all lithiation states (Fig. S44), which contributes to maintaining the structural integrity of the HES during cycling. Consequently, these element-specific spectroscopic results validate our compositional design of the HES, in which functionality is distributed across the multication lattice: Cu serves as the main electron reservoir, Co and Ni participate cooperatively in reversible redox reactions, Fe modulates the local electronic structure through coordination and covalency evolution, and Ru functions as a redox-inactive structural stabilizer.

To further elucidate the catalytic action of Li*_x_*HES in facilitating Li_2_S oxidation, interfacial electron transfer between Li*_x_*HES and Li_2_S was investigated using DFT calculations. Bader charge analysis in Fig. 4h reveals that Li_2_S donates more electrons to the highly delithiated Li_0.22_HES (4.3e–) compared to the intermediate Li_1.0_HES (3.9e–), implying that Li_2_S undergoes spontaneous chemical oxidation driven by the Li*_x_*HES matrix. This process can be understood as Li*_x_*HES acting as a solid–solid interfacial redox mediator for Li_2_S oxidation. Specifically, as illustrated in Fig. 4i, the electronically insulating Li_2_S cannot respond immediately to the applied potential during charging. Instead, the highly conductive Li_2_HES network is activated first, experiencing electrochemical delithiation to generate ‘Li-deficient state’ intermediate states (Li_2_HES-*x*e^−^→Li_2-_*_x_*HES + *x*Li^+^). Driven by their strong chemical oxidizing capability, these newly formed intermediate Li_2-_*_x_*HES phases spontaneously extract electrons from the adjacent Li_2_S (Li_2-_*_x_*HES + *y*Li_2_S→Li_2-_*_x+_*_2_*_y_*HES + *y*S) [20]. Consequently, the strong Li–S bonds in Li_2_S are substantially weakened, triggering Li_2_S decomposition and releasing Li^+^ ions into the DCC Li*_x_*HES network. Importantly, this process is self-accelerating: as the charging proceeds, continuous lithium removal yields progressively more Li-deficient states (approaching Li_0.22_HES). As corroborated by the DFT results, these deeply delithiated phases possess a stronger thermodynamic driving force for electron extraction from Li_2_S, thereby markedly accelerating Li_2_S oxidation kinetics. To experimentally corroborate this spontaneous charge transfer, a physical mixture of Li_2_S and pristine HES (representing the extreme Li-deficient state, *x* = 0) was examined using ^7^Li ssNMR. The spectrum exhibits a distinct chemical shift and broadening relative to pure Li_2_S (Fig. 4j), reflecting highly heterogeneous local Li environments. This observation provides direct evidence of the Li_2_S oxidation chemically driven by HES. Collectively, both theoretical and experimental insights establish that the Li*_x_*HES matrix is not merely as a passive DCC, but actively functions as a highly efficient solid-state redox mediator. This dynamic chemical–electrochemical coupling fundamentally overcomes the kinetic barriers of insulating Li_2_S oxidation, ensuring highly reversible and rapid sulfur redox reactions.

## CONCLUSION

In summary, this work reveals two failure mechanisms of conventional TMS catalysts in ASSLSBs, including phase separation and TM migration, and introduces a high-entropy strategy to overcome these intrinsic limitations. Compared with the conventional counterparts, the rationally designed pyrite-type HES utilizes entropy stabilization to form a highly stable Li*_x_*HES solid-solution phase during cycling, thereby avoiding irreversible catalyst self-poisoning and electrolyte degradation. This robust, all-in-one catalytic platform overcomes the spatial limitations of traditional TPBs by providing expansive dual ion-electron pathways, while thermodynamically weakening Li–S bonds to drive spontaneous Li_2_S oxidation. By translating these favorable microscale interfacial kinetics into macroscopic electrode performance, we effectively mitigate the persistent challenge of low sulfur utilization in ASSLSBs with high-sulfur-content cathodes. Specifically, with an ultra-low HES addition of only 3 wt%, the obtained S@HES cathode (40 wt% S) achieves exceptional sulfur utilization of 93.4% and a conversion efficiency of 93.0%. It also sustains superior high-rate cycling performance (900 mAh g^−1^ at 2C over 250 cycles) even with an elevated sulfur fraction (50 wt%). Notably, the outstanding catalytic efficacy is preserved under demanding constraints (8.5 mg cm^−2^, 30°C), yielding a remarkable areal capacity of 9.68 mAh cm^−2^. The practical viability of HES is further demonstrated in a S@HES|LPSC|Li_3.75_Si full cell, which delivers a highly competitive gravimetric energy density of 571 Wh kg^−1^. This study offers profound mechanistic insights into solid-state catalytic failures and underscores the significant potential of the high-entropy strategy for advanced solid-state Li–S catalysis.

## METHODS

### Synthesis of HES

HES was synthesized using the high-pressure and high-temperature Cubic Press (GY420, Guilin, China) at HPSTAR. All chemicals, including iron, cobalt, nickel, copper, ruthenium nanopowder and sulfur powder, were sourced from Alfa Aesar and used without additional purification. First, the raw materials were weighed in an argon glovebox in the desired stoichiometric ratio, and then ball-milled using a high-energy mechanical mill (SPEX 8000D). Subsequently, the homogenized powders were prepressed at 20 MPa into a cylinder, followed by encapsulation within a hexagonal boron nitride (h-BN) capsule. Then, the assembled block was sintered at 1400°C under 5 GPa for 40 min.

### Preparation of composite cathodes

HES nanopowders were obtained by further refining the sintered samples using high-energy mechanical milling. The milling was performed at a rotation speed of 1725 rad min^−1^ for 10 h under an argon atmosphere.

Sulfur-based composite cathodes were prepared via a two-step ball-milling process using a planetary micro mill (Fritsch Pulverisette 7). Sulfur and AB (TIMCAL Graphite & Carbon) were dried prior to use and transferred into an argon-filled glove box (MIKROUNA, O_2_ and H_2_O <0.01 ppm). In the first step, sulfur and AB were mixed with mass ratios of S:AB = 3:2, 2:1 and 5:2, which were designed to yield final sulfur contents of 30 wt%, 40 wt% and 50 wt%, respectively, in the composite cathodes. The mixtures were loaded into a ZrO_2_ milling jar with ZrO_2_ balls and sealed under an inert argon atmosphere. Ball milling was carried out at 500 rad min^−1^ for 10 min, followed by a 5-min rest period for cooling; this sequence was repeated for a total of 48 cycles. In the second step, the as-prepared AB/S mixtures were combined with LPS (Hefei Kejing Materials Technology Co., Ltd.) solid electrolyte at mass ratios of 1:1, 3:2 and 7:3 to obtain composite cathodes with sulfur contents of 30 wt%, 40 wt% and 50 wt%, respectively. The mixtures were further milled at 400 rad min^−1^ for 36 cycles to ensure homogeneous dispersion. For the preparation of S@HES composite cathodes, a modification was applied only in the initial ball-milling step, where 15 wt% of AB was replaced by HES, while all other processing parameters remained identical. This substitution resulted in an HES content of ∼3 wt% in the final composite cathodes.

For composite cathodes based on HES, TMS_2_ and TMS, a similar preparation procedure was adopted. Here, TMS_2_ denotes metal disulfides (TM = Ti or Fe), and TMS represents metal monosulfides (TM = Fe, Co, Ni or Cu). The metal sulfide powder (active material), LPS and vapor-grown carbon fiber (Shenzhen Kejing Star Technology Co., Ltd.) were mixed at a weight ratio of 31:62:7, respectively, and loaded into a ZrO_2_ milling jar. Mechanical milling was conducted at 150 rad min^−1^ for 1 h.

## Supplementary Material

nwag501_Supplemental_File
