# Supplementary material for: Unlocking high-sulfur-content cathodes by an all-in-one high-entropy sulfide catalyst for all-solid-state Li–S batteries
Source: Natl Sci Rev. 2026 Aug 18;13(18):nwag501. doi: 10.1093/nsr/nwag501 (PMC13637574; doi:10.1093/nsr/nwag501)
Supplement: Supplementary file 1 [file nwag501_supplemental_file.pdf]

**Supplementary Information**

# Unlocking high-sulfur-content cathodes by an all-in-one high-entropy sulfide catalyst for all-solid-state Li-S batteries

Ruqin Ma[1,†], Yong Cheng[1,†], Mintao Su[1,†], Hongxin Lin[1], Chuanjing Xu[1], Wenhua Zuo[1], Siyuan Pan[1], Gongkai Wang[3], Yu Luo[1], Chenpeng Xie[1], Zhifeng He[1], Haoyue Zhong[1], Yuqi Wu[1], Weilin Huang[1], Huiyang Gou[4], Zhengliang Gong[1], Ming-Sheng Wang[1,5,*] and Yong Yang[1,2,*]

[1]State Key Laboratory for Physical Chemistry of Solid Surface, College of Chemistry and Chemical Engineering, College of Materials, College of Energy, Xiamen University, Xiamen 361005, China;

[2]Innovation Laboratory for Sciences and Technologies of Energy Materials (IKKEM), Xiamen 361102, China;

[3]School of Material Science and Engineering, 'The Belt and Road Initiative' Advanced Materials International Joint Research Center of Hebei Province, Hebei University of Technology, Tianjin 300130, China;

[4]Center for High Pressure Science and Technology Advanced Research, Beijing 100193, China;

[5]Shenzhen Research Institute of Xiamen University, Shenzhen 518057, China

***Corresponding authors.** E-mails: mswang@xmu.edu.cn; yyang@xmu.edu.cn

[†]Equally contributed to this work.

**Table of Contents:**

Methods

Figs. S1-45

Section S1

Tables S1-6

References

## METHODS

### Preparation of all-solid-state batteries

For the Li-Si anode, lithium metal and silicon powder were mixed at a molar ratio of 3.75:1 and subsequently transferred into a $ZrO_2$ jar. High-energy ball milling was performed at 400 rpm for 10 h.

For all-solid-state sheet-type cells, the preparation of dry-processed membranes and the cell assembly procedures were carried out following our previously reported methods [1]. For cells using a Li–In film anode, a single-layer electrolyte film containing 1 wt% PTFE was used. For cells using a $Li_{3.75}Si$ alloy anode, a bilayer electrolyte film was adopted, in which the cathode-side electrolyte film contained 1 wt% PTFE and the anode-side electrolyte film contained 0.2 wt% PTFE.

### Cell assembly

For cell assembly, an in-house cell was employed. Initially, a piece of In (100 mg) and Li (5 mg) were first pressed together at 20 MPa to obtain a Li-In alloy anode. Then 90 mg of $Li_6PS_5Cl$ powder (LPSC, China Automotive Battery Research Institute) was cold-pressed using a hydraulic machine at a pressure of 42 MPa for 1 minute in a polyether ether ketone cylinder (PEEK) with an inner diameter of 10 mm. Subsequently, the composite cathode was uniformly sprinkled onto one side of the SSE pellet, followed by uniaxial pressing at 420 MPa for 3 minutes. The sulfur mass loading of ASSLSBs ranged from 1.5 to 8.5 mg $cm^{-2}$. On the opposite side of the pellet, the prepared Li-In alloy or Li-Si powder were positioned and cold-pressed at 250 MPa for 1 minute. The entire battery assembly process was conducted within an argon-filled glove box.

### Operando electrochemical-pressure measurements

Lithiated $Li_4Ti_5O_{12}$(LTO) was prepared according to previous literature[2]. The anode composite was prepared by mixing lithiated LTO powder, LPSC, and VGCF at an optimized weight ratio of 50:47:3. The mixture was sealed in a ball-milling jar and milled for 1 hour at 200 rpm using a planetary ball mill within an argon-filled glovebox. Lithiated LTO|$TMS_2$ cells, with an areal $TMS_2$ loading of 0.4 mg $cm^{-2}$ and an anode

loading of 9.5 mg $cm^{-2}$, were cycled at a current density of 38 μA $cm^{-2}$ and a temperature of 30°C, with a voltage range of -0.3-1.4 V. Before cycling, the assembled battery was applied an initial stack pressure of 2000 N for 20 hours to minimize pressure baseline drift. All pressure variations during cycling were meticulously tracked using the system obtained from Beijing Zhongke Wanyuan Technology Co., Ltd.

**Electrochemical Characterization**

Electrochemical evaluations of the ASSLSBs were conducted using the Neware testing system. Galvanostatic measurements were conducted at various current densities (1C = 1675 mA $g^{-1}$) within a voltage range of 0.68-2.48 V for ASSLSBs with LiIn anode and 0.8-3 V for LiSi anode, at temperatures ranging from 30 to 55 °C. For long-term cycling tests at elevated temperature (55 °C), the voltage window was restricted to 0.78-2.48 V to prevent irreversible electrolyte decomposition in the low-voltage region caused by reduced polarization at high temperature. CV and EIS measurements were carried out using a multichannel electrochemical workstation (PARSTAT, Princeton Applied Research). EIS tests employed a voltage amplitude of 10 mV within a frequency range of 1 MHz to 1 Hz. EIS results were analyzed using the Zview software with equivalent circuits models. All electrochemical experiments were conducted within a climatic chamber at a temperature of 30 ± 2 °C.

**Materials Characterization**

ICP-OES measurements were performed on SPECTRO SPECTROBLUE FMX36. Prior to analysis, the samples were fully dissolved in aqua regia. The morphology of samples was examined by FESEM (Hitachi S-4800) operated at an accelerating voltage of 15 kV.

Powder XRD analysis was conducted on a Rigaku Ultima IV diffractometer using Cu Kα radiation (40 kV, 30 mA), over a 2θ range of 30-80° with a scanning rate of 2° $min^{-1}$. Rietveld refinement of the XRD patterns was performed using a General Structure Analysis System (GSAS) software.

The single-particle compression tests were conducted using a single-particle mechanical property testing system (Initial Energy Science & Technology Co., Ltd, China).

The ssNMR experiments were conducted on a Bruker Avance III 400 MHz spectrometer equipped with a 1.3 mm magic-angle spinning (MAS) probe at a spinning rate of 50 kHz. EPR was recorded on a Bruker EMXplus-9.5/12. The measurements were conducted in X-Band (9.81792GHz), with amplitude modulation of 1 G, microwave power of 20 mW, an amplitude modulation frequency of 100 kHz, and conversion time of 4 ms.

Transition-metal *K*-edge XAS measurements were performed at the BL13SSW hard X-ray beamline station of the Shanghai Synchrotron Radiation Facility (SSRF) in fluorescence mode, with the concentration of each transition-metal element controlled at approximately 3 wt%. To avoid air and moisture exposure, both electrodes and reference materials were sealed with Mylar film prior to measurements. XANES spectra were extracted by subtracting the pre-edge background and normalizing to the post-edge region using a spline fit with the ATHENA software package. The $k^2$-weighted EXAFS spectra were Fourier transformed over a k-range of 3-10 $Å^{-1}$ using a Hanning window ($\Delta k = 1$ $Å^{-1}$). EXAFS fitting was performed with the FEFF database using the Artemis software package.

Cross-sectional SEM images and TOF-SIMS analyses were performed using a FIB system (Crossbeam 350) at the Innovation Laboratory for Sciences and Technologies of Energy Materials (IKKEM). Depth profile analyses were conducted using a $Ga^+$ primary ion beam with an energy of 30 keV and a sputtering current of 50 pA. The analysis area was 3×3 $um^2$, with a sputtering raster area of 120×120 $um^2$. The TEM, HADDF-STEM, and HR-S/TEM measurements were performed on a double Cs-corrected TEM (Titan Themis Z, FEI, USA) with a Super-X EDS detector operated at 300 kV.

*In-situ* XRD measurements were conducted using homemade cells following procedures reported in our previous work[3]. A 2032 coin cell with a hole on the cathode side was externally sealed with Mylar film, while a thin aluminum foil was applied internally to seal the hole and serve as the current collector. After cold pressing with a Li-In alloy anode, the pellet was carefully removed from the mold and assembled into the *in-situ* XRD cell. The measurements were carried out on a Bruker D8 Discover

diffractometer using Cu Kα radiation (λ = 1.5406 Å).

XPS measurements were carried out using Thermo Scientific Escalab 250Xi$^+$ system. All spectra were calibrated with respect to the C 1s peak at 284.8 eV arising from surface hydrocarbon contamination.

**Theoretical calculations**

First-Principles calculations based on density functional theory (DFT) were carried out using the Vienna Ab-initio Simulation Package (VASP). The generalized gradient approximation (GGA) with the Perdew-Burke-Ernzerhof (PBE) functional[4] was selected as the exchange-correlation potential, and the projector-augmented wave (PAW) method[5, 6] were adopted for structural calculations under spin-polarization. The Hubbard U correction factors were employed to account for electron-electron interactions in the localized 3d orbitals, the U values based on reported works[7–9], were set as 2 eV, 3 eV, 5 eV, 2 eV and 5.2 eV for Fe, Co, Ni, Cu and Ru, respectively. The special quasi random structure (SQS)[10] $Li_2(Fe_2Co_2Ni_2Cu_2Ru)_{1/9}S_2$ supercell with 36 Li sites, 18 transition metal sites and 36 S sites was obtained from Alloy Theoretic Automated Toolkit (ATAT)[11] by creating 3×3×2 supercell of $Li_2FeS_2$, which used Monte Carlo algorithm to find the SQS. Atomic positions, supercell volume and shape were fully relaxed. To enhance computational accuracy, following official VASP recommendations, we used the PBE functional including inner p orbitals to treat the electronic states of Li (1s, 2s) and Ru (4p, 5s, 4d). Consequently, the energy cutoff for the DFT calculations was set to 600 eV. The integration in the Brillouin zone was operated on a set of K-points determined by the Monkhorst and Pack theme: 3×3×3 for $Li_2(Fe_2Co_2Ni_2Cu_2Ru)_{1/9}S_2$ supercell, the criterion for the energy convergence to $10^{-5}$ eV and the maximum force in cell were less than 0.02 eV/Å while running structure relaxation. Crystal surfaces of the (1 1 1) plane for the pyrite-like HES and the (1 0 0) plane for $Li_2S$ were selected to construct the heterojunctions. The interfacial separation was set to 2 Å, and a vacuum layer thickness of 15 Å was applied along the direction perpendicular to the interface. The mismatching of surfaces was controlled no more than 7%. To eliminate the mismatching caused by distorted HES structures, atomic

positions cell shape and volume were relaxed, the standards of convergence are same as former calculations.

To figure out the stability of high entropy sulfide $Li_2(Fe_2Co_2Ni_2Cu_2Ru)_{1/9}S_2$ in different temperature, we used density-functional-perturbation theory (DFPT) method to calculate the supercell of high entropy sulfide and possible decomposition products for getting the phonon modes and frequencies of the systems. After making sure that there is no imaginary frequency in the result , Phonopy[12] was applied to calculate the zero-point energy and entropy in different temperature. According to formula $\Delta G=\Delta(E+E_{ZPE})-T\cdot\Delta S$ , we calculated the free energy of high entropy sulfide decomposition reactions at 300 K.

The properties of the chemical bonds within the systems were further explored through the -ICOHP analysis, utilizing the LOBSTER[13] program. This approach allowed for a more detailed examination of changes in the strength of chemical bonds, To avoid potential misinterpretation of bonding states due to Ru inner orbitals in the -ICOHP analysis, we chose the standard PBE functional describing only the Ru (5s, 4d) valence states, sacrificing some accuracy in these specific calculations.

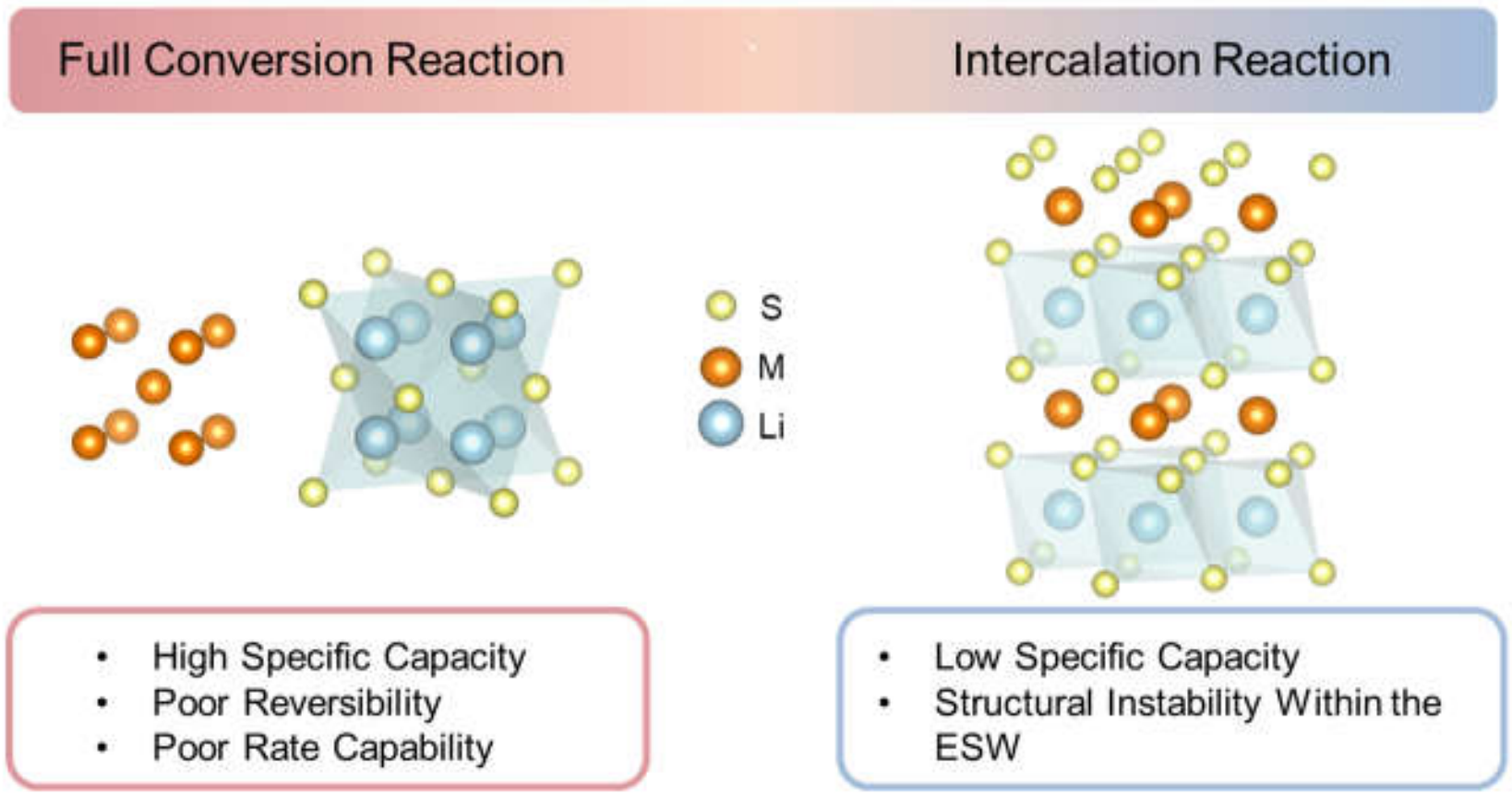


**Fig. S1** Schematic comparison of the characteristic features and discharge-product structures of conversion-type TMSs and intercalation-type TMSs.

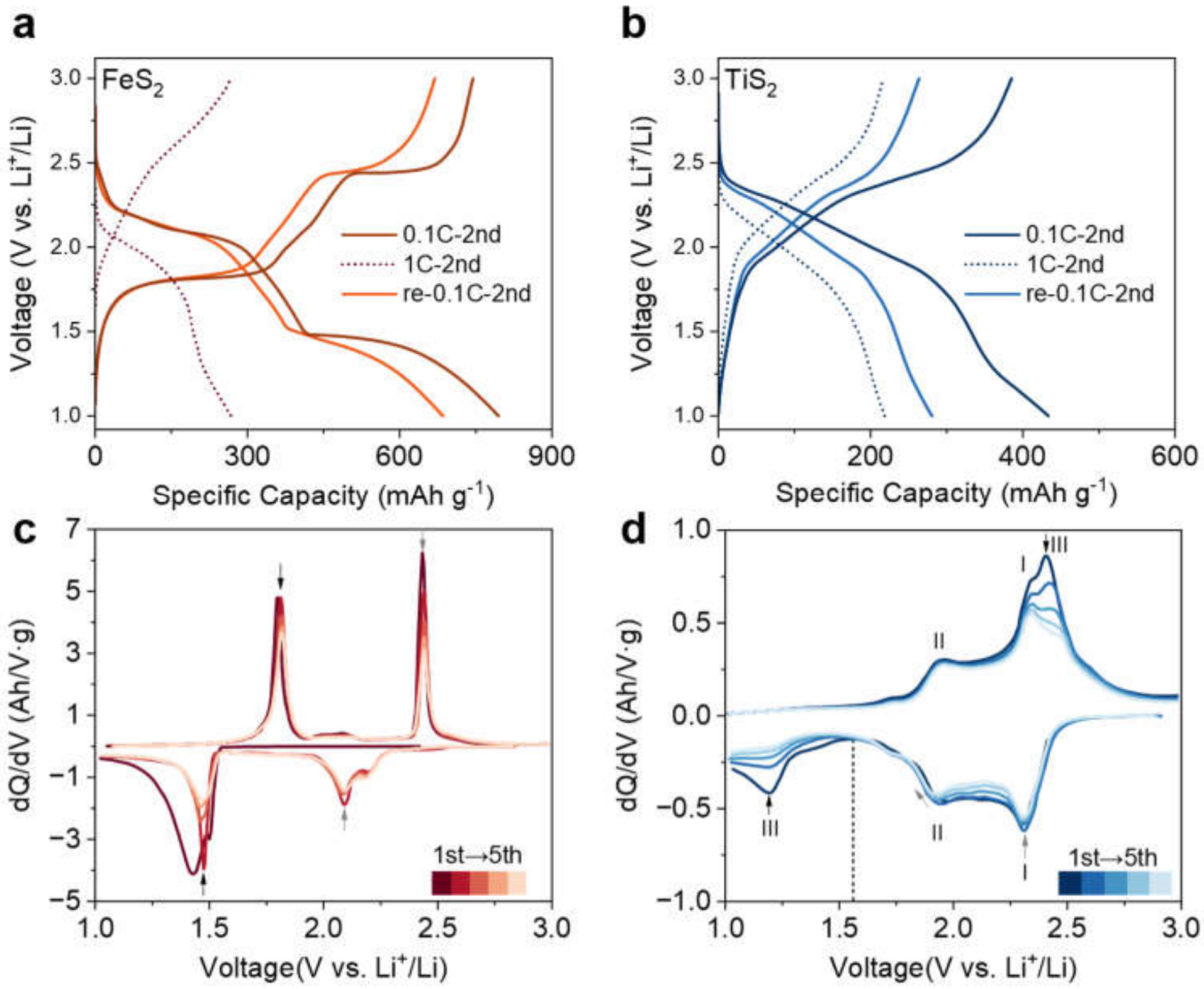


**Fig. S2** Electrochemical behavior of $FeS_2$ and $TiS_2$ cathodes within the 1–3 V vs. $Li^+/Li$ electrochemical window. (a-b) Detailed discharge-charge curves at selected cycles in ASSLSBs with $FeS_2$ (a) and $TiS_2$ (b) cathodes. (c-d) dQ/dV curves of the first five discharge–charge cycles measured at 0.1 C for ASSLSBs with $FeS_2$ (c) and $TiS_2$ (d) cathodes. Notably, $FeS_2$ exhibits abrupt and highly concentrated phase-transition peaks

within a narrow voltage range, whereas $TiS_2$ shows a broader solid-solution–type transition region with relatively symmetric redox peaks (Peaks I and II).

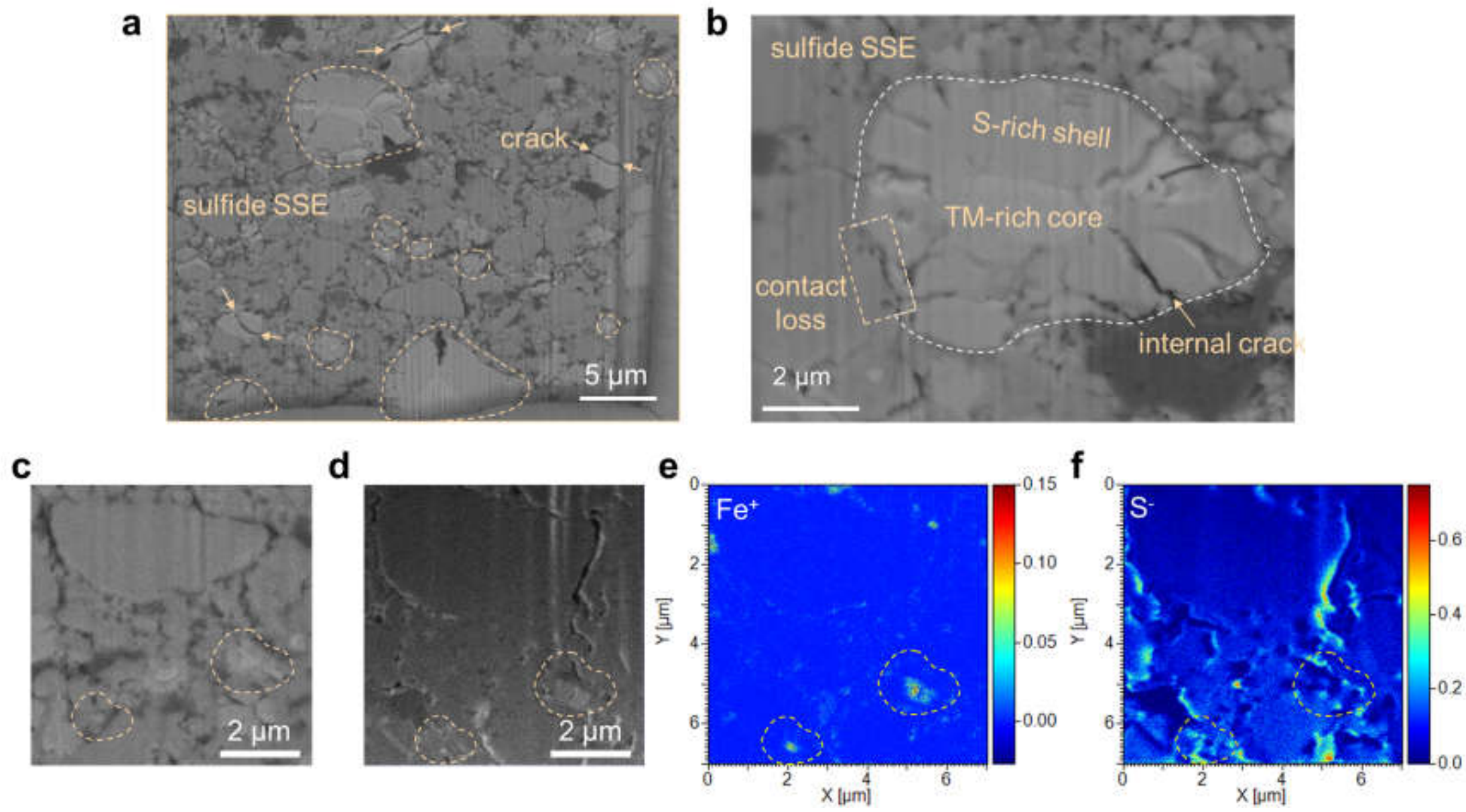


**Fig. S3** Structural degradation and phase separation in $FeS_2$ composite cathodes after discharge. (a-b) FIB–SEM images of a $FeS_2$ composite cathode after discharge to 1.3 V vs. $Li^+$/Li. The dashed outlines mark $FeS_2$ particles. Both micrometer-sized and nanometer-sized $FeS_2$ particles exhibit pronounced phase separation and internal cracking after discharge. (c-f) ToF-SIMS analysis. (c) Secondary electron (SE2) image showing contrast differences among distinct phases, acquired with the field of view parallel to the sample surface. (d) $Ga^+$ image collected using the SE detector at a tilt angle of 36° relative to the sample surface. (e-f) Corresponding planar distributions of $Fe^+$ (m/z=56) and $S^-$ (m/z=32) fragments acquired from the field of view shown in (d), respectively.

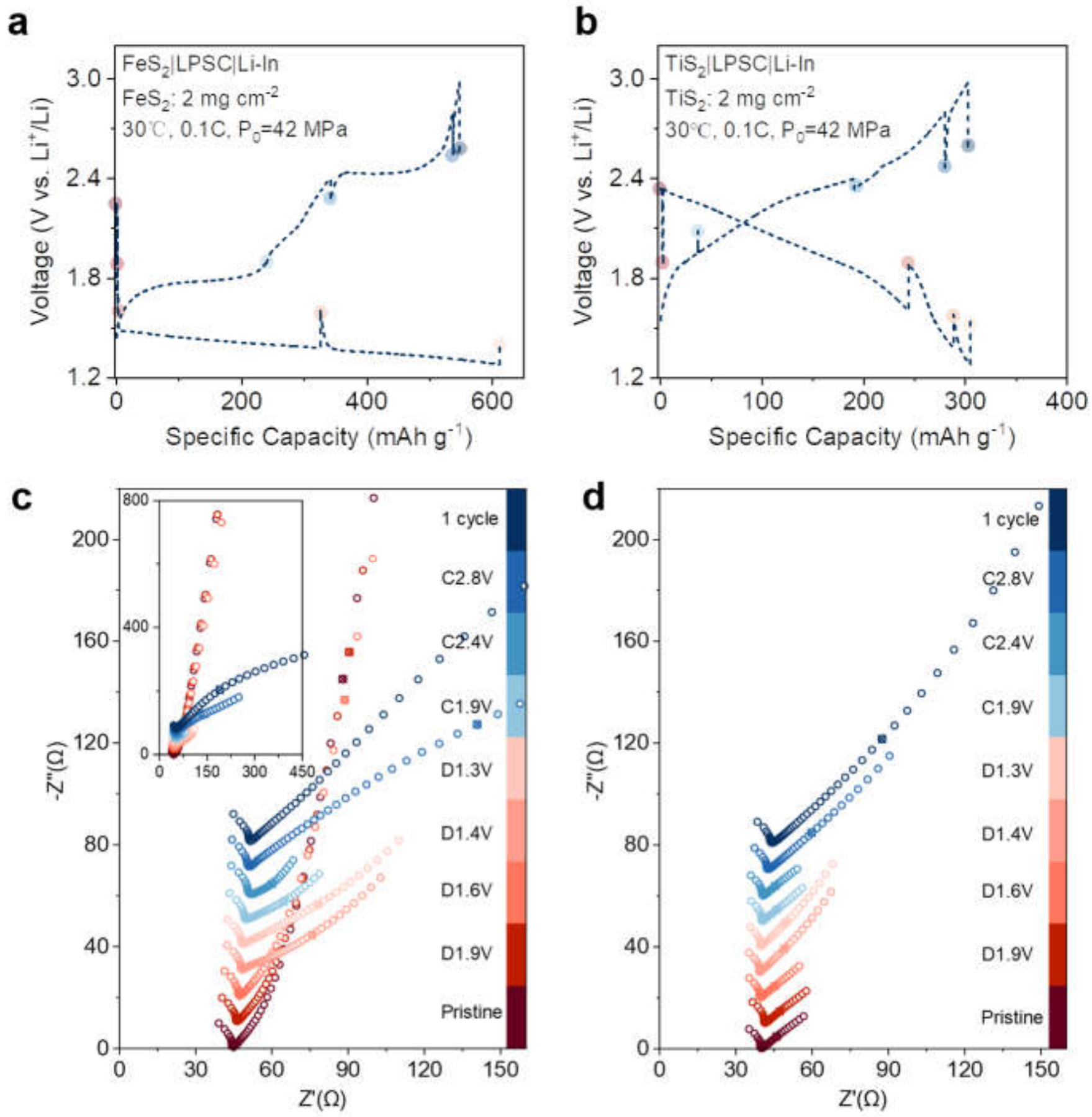


**Fig. S4** In situ EIS analysis of $FeS_2$ and $TiS_2$ within 1.3-3 V vs. $Li^+$/Li. (a-b) Selected states for in situ EIS measurements of (a) $FeS_2$ and (b) $TiS_2$, together with the corresponding electrochemical profiles. (c-d) Nyquist plots derived from the in situ EIS measurements of $FeS_2$ (c) and $TiS_2$ (d). In the high-frequency region, the incomplete semicircle is attributed to the grain-boundary resistance of the SEs, followed by contributions from interfacial resistance ($R_{int}$) and charge-transfer resistance ($R_{ct}$). Owing to the poor resolution of overlapping semicircles, the impedance magnitude at 1 Hz (marked by "⊗") is used as a representative metric to track the evolution of a comparable time constant with state of charge.

For $FeS_2$, the pristine electrode exhibits pronounced low-frequency blocking behavior, characterized by a diffusion tail with a slope substantially greater than 45°, consistent with limited ionic transport through the bulk phase. Upon discharge, the blocking behavior is partially alleviated, whereas the overall ohmic resistance increases, indicative of the formation of less conductive or electrochemically inert species. After

completion of the first charge, the impedance at 1 Hz increases sharply and the low-frequency response deviates from the pristine profile, consistent with irreversible structural or interfacial changes.

In contrast, $TiS_2$ initially displays a near-ideal Warburg-type diffusion tail (~45° slope), reflecting facile $Li^+$ transport within the bulk. After discharging to 1.6 V vs. $Li^+/Li$, incomplete voltage recovery upon relaxation is observed. Further discharge to 1.3 V results in a continued increase in overall impedance, coinciding with the emergence of an additional reduction feature (Peak III, Fig. S2d). Moreover, the impedance rise at the end of charge suggests cumulative and irreversible degradation within the composite cathode.

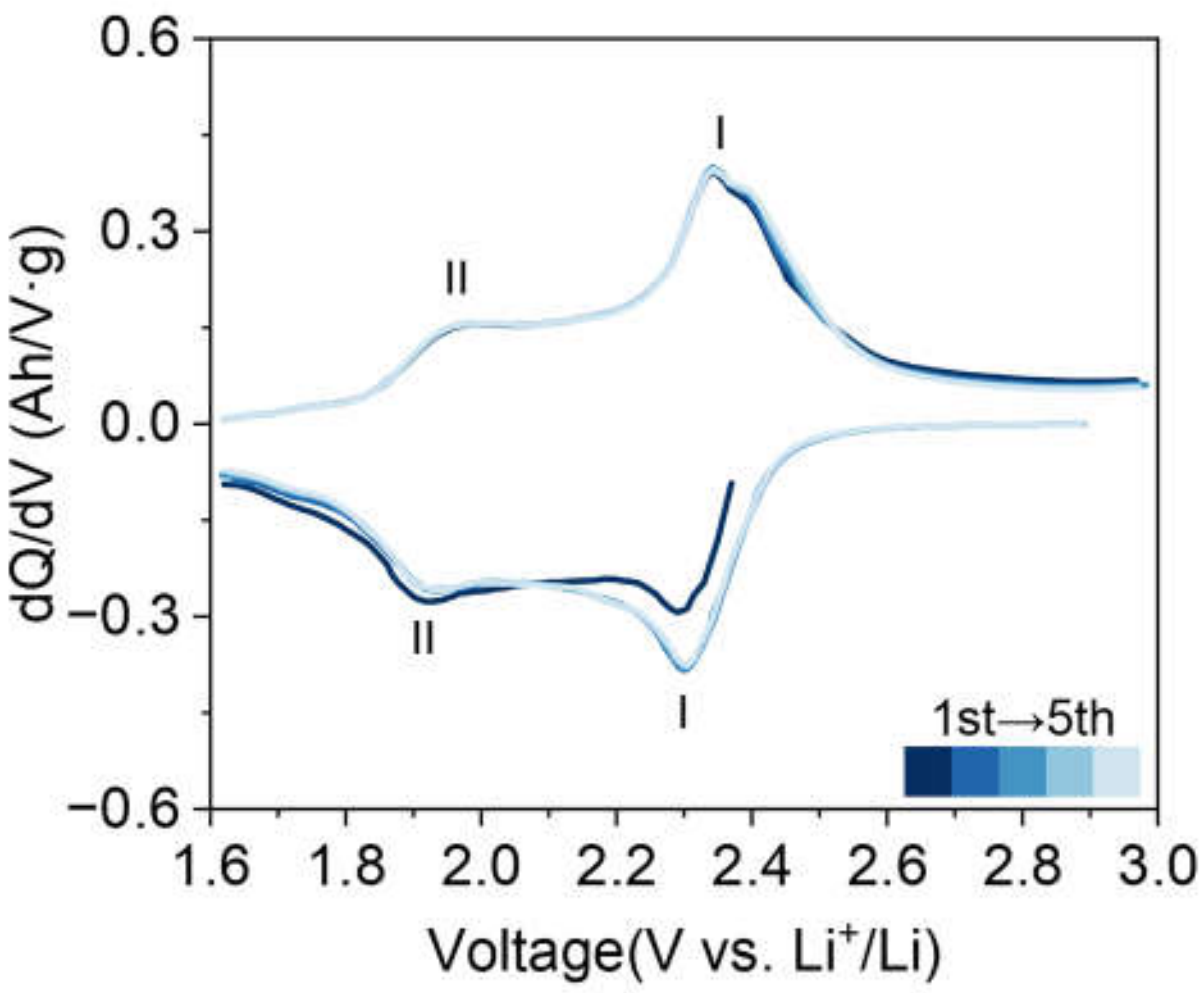


**Fig. S5** dQ/dV curves of the first five discharge–charge cycles measured at 0.1 C for ASSLSBs with $TiS_2$ cathodes in the voltage range of 1.6-3 V vs. $Li^+/Li$.

**Section S1** Effect of transition-metal diffusion on the sulfide electrolytes

Transition-metal diffusion into sulfide-based SSEs has been widely reported for various cations, including $Cu^+$[14, 15], $Ti^{4+}$[16], $Ni^{2+}$[17], $Co^{2+}$[17, 18], and $Mn^{2+}$[17]. Depending on the specific electrolyte composition and interfacial chemistry, such diffusion can exert divergent effects. In certain systems, the formation of mixed ionic–electronic conducting interphases has been shown to facilitate charge transport by establishing dual-conduction pathways[14]. In contrast, excessive incorporation of transition metals

may destabilize the sulfide framework, leading to bulk electrolyte decomposition and degradation of electrochemical stability[15]. Consequently, the impact of metal diffusion is highly system-dependent and closely linked to the intended functional role of the metal-containing phase.

In this work, TMSs were utilized as catalytic components for sulfur conversion. From a catalyst-design perspective, preserving a structurally and electrochemically stable catalyst–electrolyte interface is essential to maintain catalytic functionality without perturbing the intrinsic properties of the SSE. Once transition-metal ions migrate into the electrolyte, they effectively become sacrificial species that react with the SSE matrix, resulting in the loss of catalytic activity and progressive interfacial destabilization.

A representative example is $TiS_2$. Previous studies have shown that blending $TiS_2$ with sulfide SSE leads to Ti migration and the formation of Li–Ti–P–S phases, which increase electronic conductivity and enable mixed conduction across the interface[19]. However, such diffusion simultaneously reduces electrochemical stability, leading to irreversible reduction peaks below 1.4 V vs. $Li^+/Li$ and continuous capacity fading within 1–3 V during cycling[16, 20]. In our experiments, similar behavior was observed: in Fig. S2d, $TiS_2$–$Li_7P_3S_{11}$ composites exhibited capacities exceeding the theoretical value of 239 mAh $g^{-1}$ and displayed an additional irreversible redox feature (Peak III). The intensity of Peak III decreased as the discharge cutoff voltage increased to 1.6 V Fig. S5), suggesting that the extra capacity arises from the conversion of the Li–Ti–P–S phase, whose reversibility depends on the applied voltage window.

Furthermore, diffusion of $Ni^{2+}$ and $Cu^+$ ions was also found to adversely impact the SSE stability. In NiS- and CuS-containing composites (Figs. S6c-e), pronounced activation behavior was observed during the first charging process, characterized by additional oxidative decomposition features at high voltages. Such processes likely involve the formation of electrochemically inactive sulfur species[14] and the disruption of ionic transport networks within the SSEs[15].

Overall, these observations underscore the critical role of transition-metal diffusion at catalyst–SSE interfaces in governing the long-term electrochemical performance of

sulfide-based solid-state batteries. Suppressing uncontrolled metal migration, while preserving catalytic functionality, emerges as a key requirement for constructing durable interfacial architectures in high-performance solid-state sulfur systems.

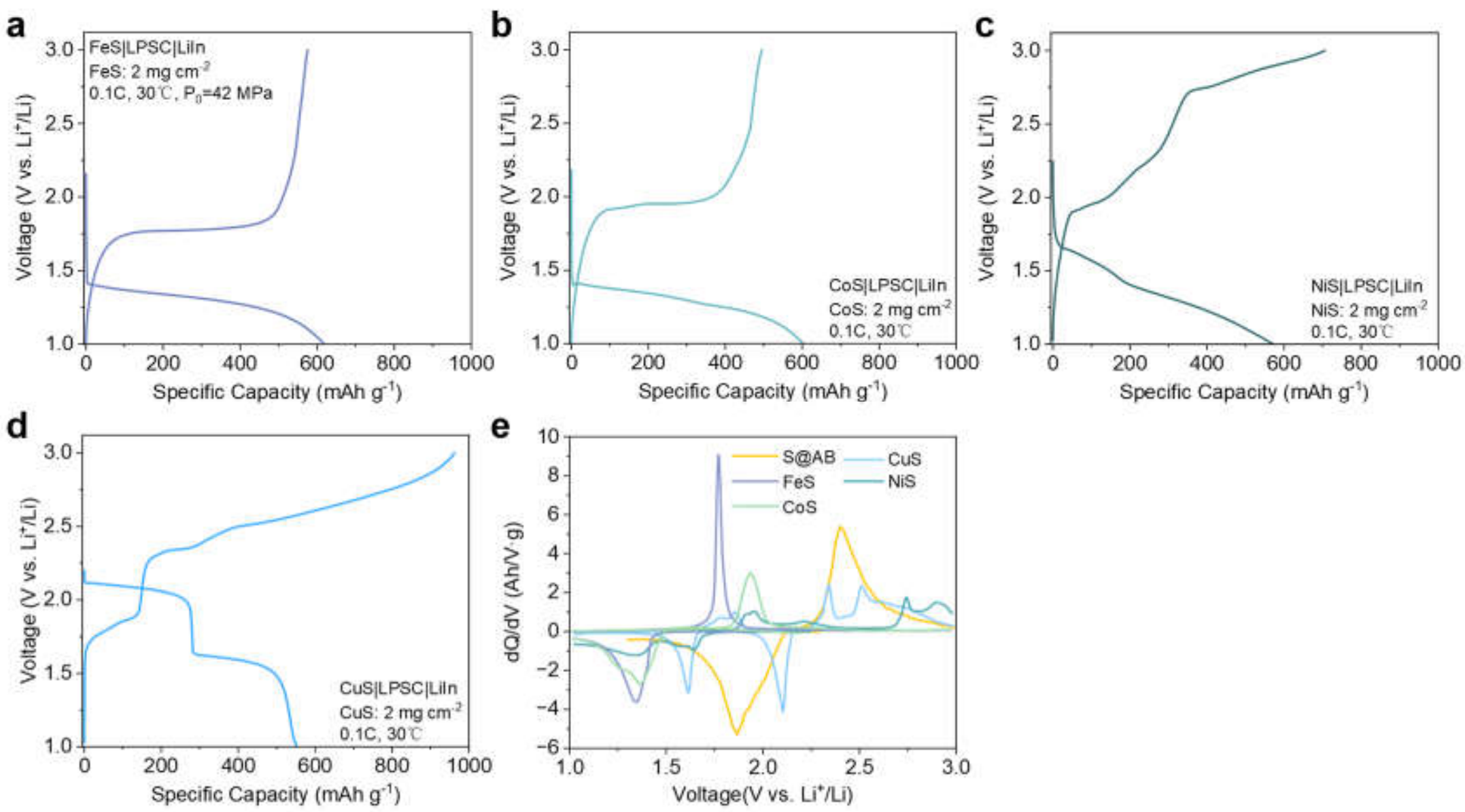


**Fig. S6** Electrochemical redox windows of selected transition-metal sulfides. (a–d) Discharge-charge curves of TMS (M = Fe (a), Co (b), Ni (c), and Cu (d)) measured to determine the redox potential ranges of the selected transition-metal cations. Note that $TMS_2$ phases were intentionally not used in these measurements to exclude contributions from anionic redox processes. (e) Comparison of dQ/dV profiles between TMS and ASSLSBs employing S@AB composite cathodes, showing that the redox potential ranges of the transition-metal cations in TMS largely and continuously overlap with the $S^{2-}$/S redox window.

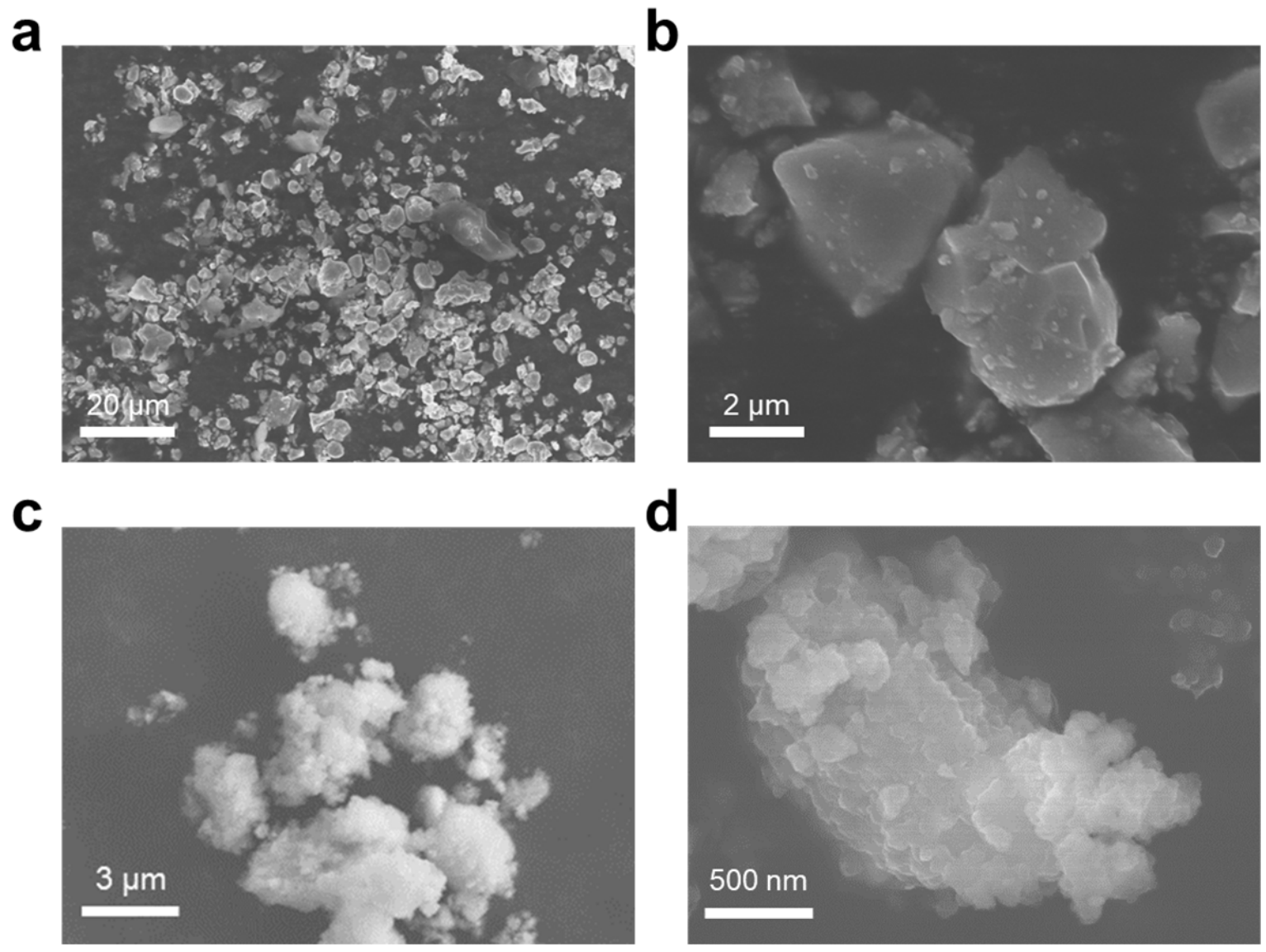


**Fig. S7** SEM images of the HES morphology. (a-b) Micrometer-sized particles before high-energy ball milling; (c-d) nanosized particles after high-energy ball milling.

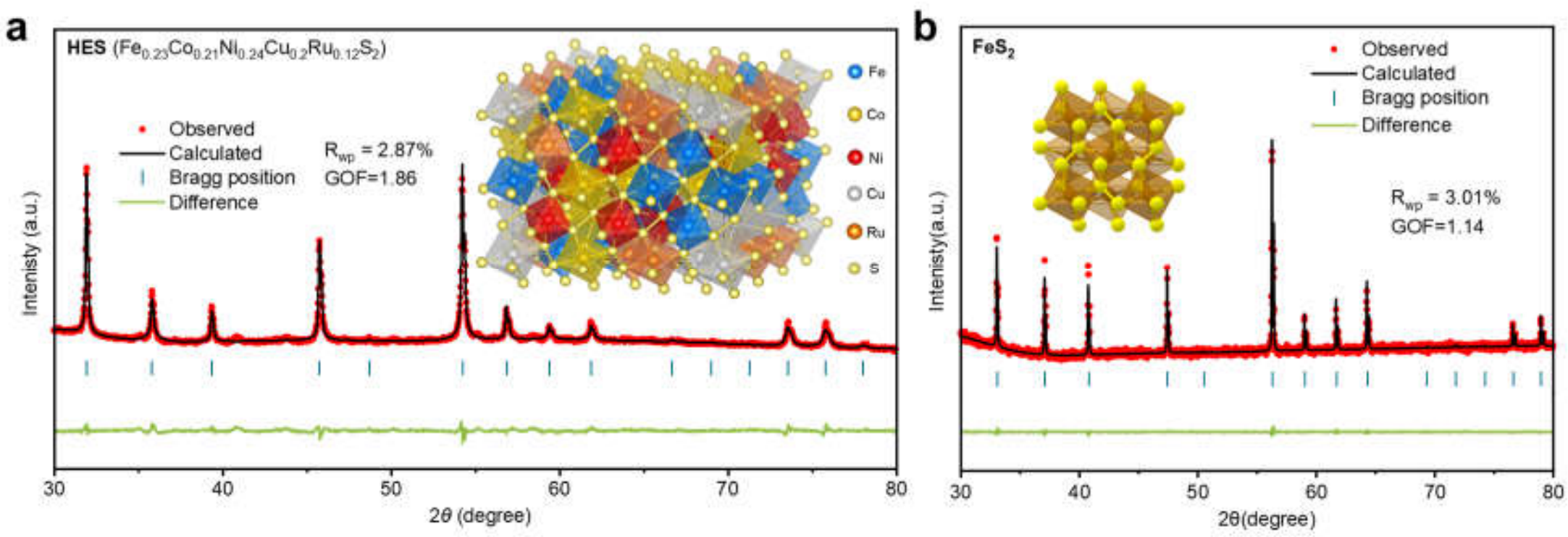


**Fig. S8** Structural refinement of HES and $FeS_2$. (a-b) Rietveld refinement results for HES (a) and $FeS_2$ (b). The weighted profile R-factor ($R_{wp}$) and the goodness of fit (GOF) are provided as indicators of the refinement quality, reflecting the agreement between the calculated and experimental diffraction patterns.

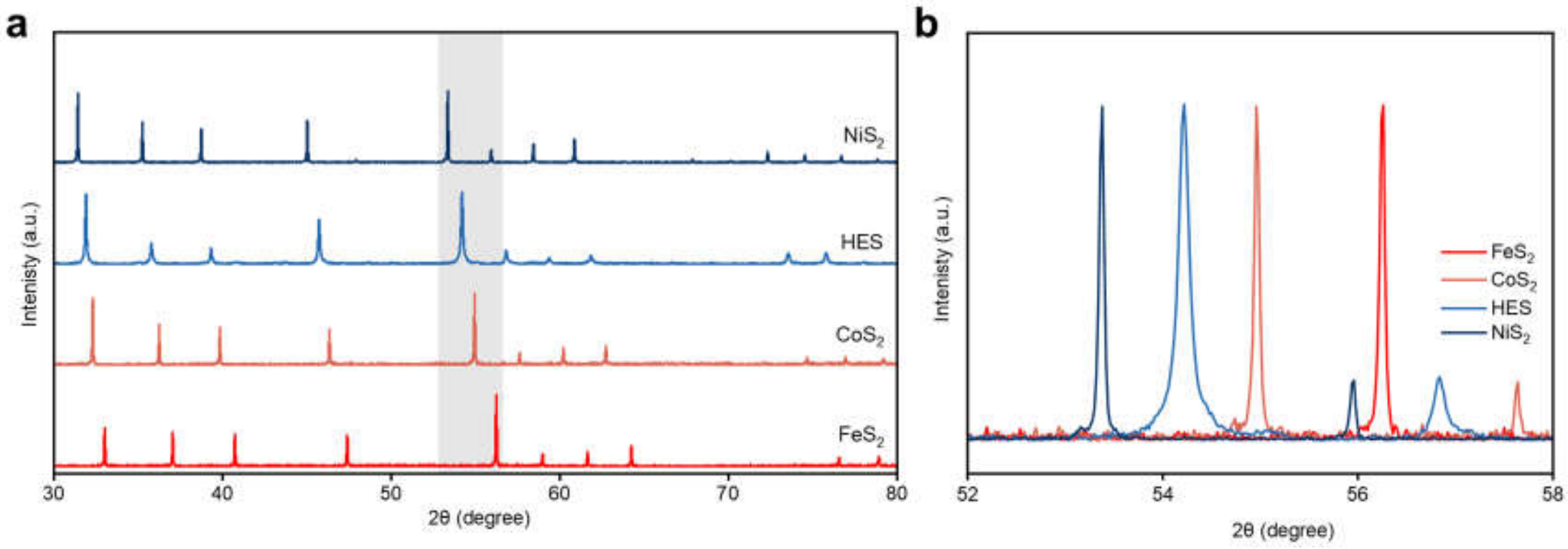


**Fig. S9** Comparison of XRD peak positions of pyrite-type sulfides (HES, $NiS_2$, $CoS_2$ and $FeS_2$).

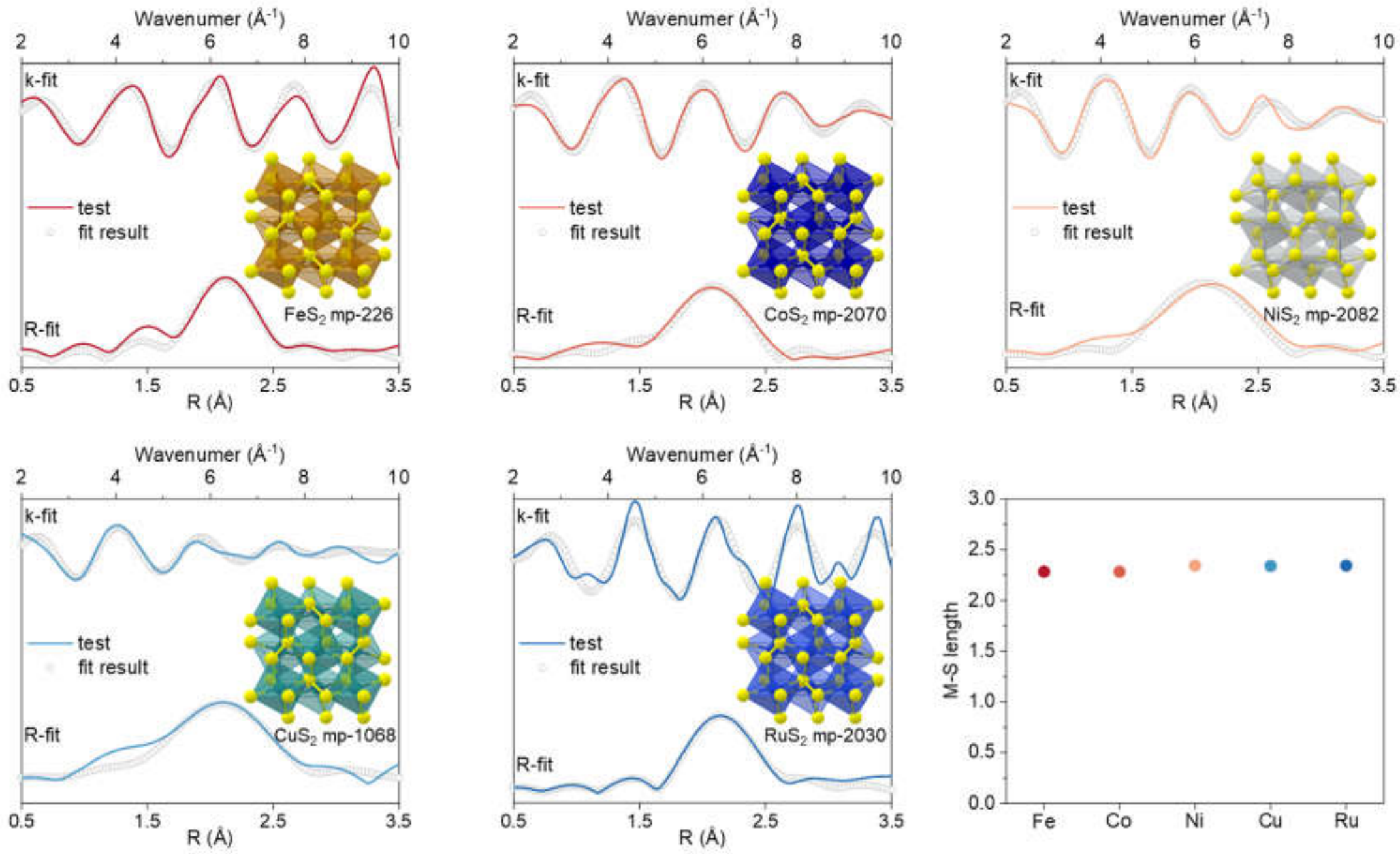


**Fig. S10** EXAFS spectra collected at the Fe, Co, Ru, Cu, and Ni K-edges are shown. The Fourier-transformed EXAFS signals, displayed in the lower panels, whereas the corresponding $k^2$-weighted EXAFS spectra are shown in the upper panels.

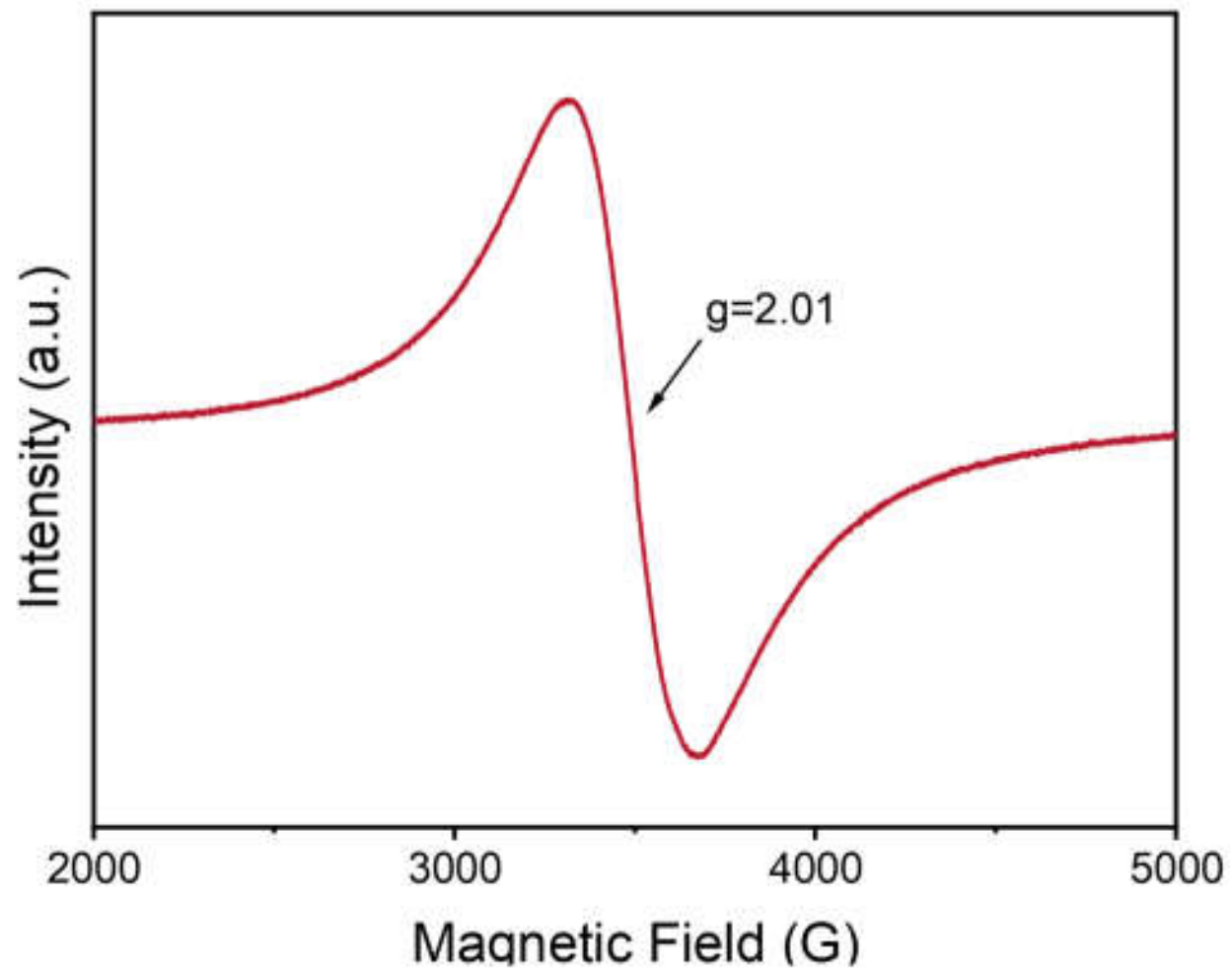


**Fig. S11** EPR spectra of HES showing a sharp resonance at g=2.01, indicative of electrons localized at anion vacancies[21].

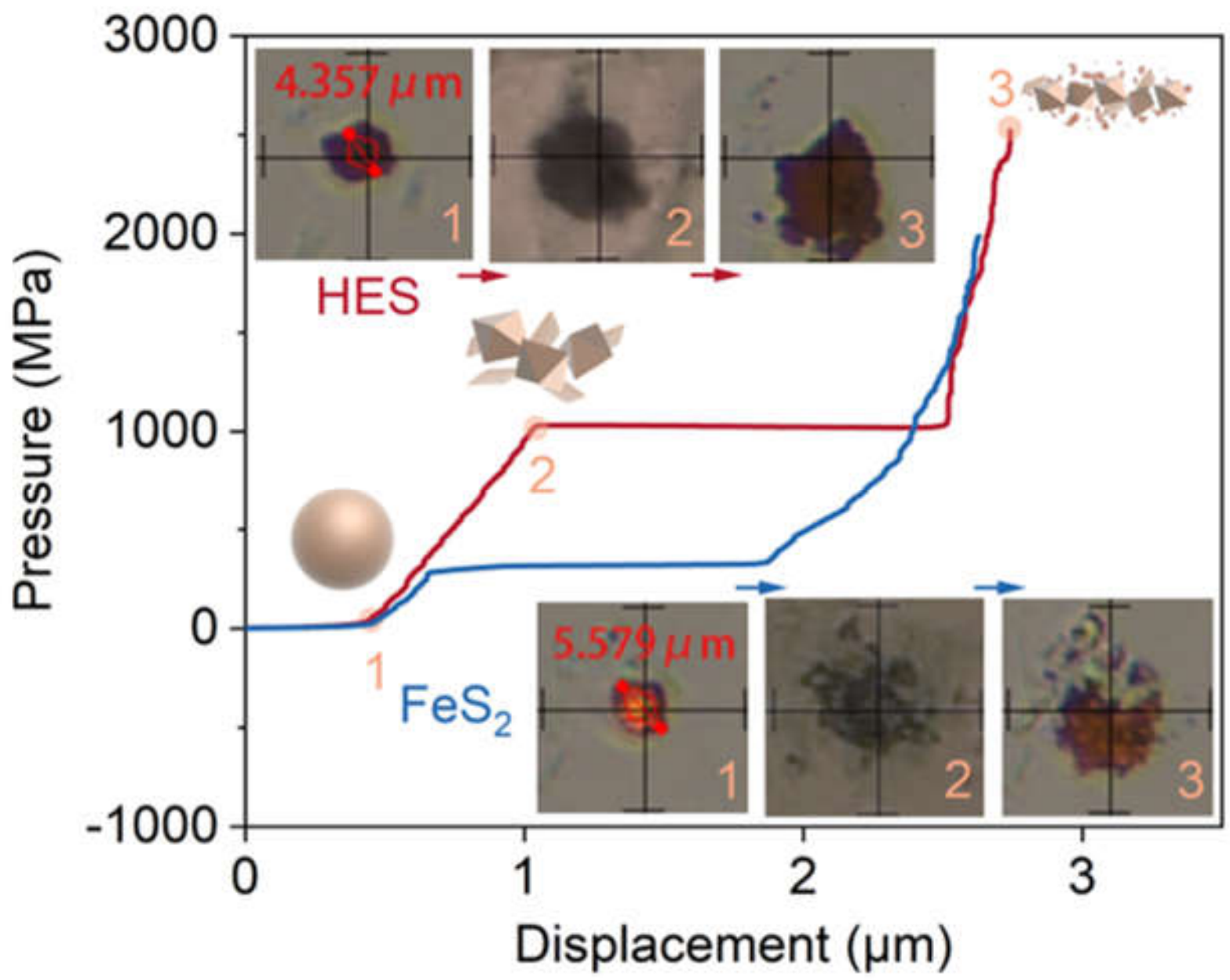


**Fig. S12** Single-particle compression test comparison between pristine HES and $FeS_2$. $FeS_2$ exhibits pronounced brittle behavior, releasing mechanical stress primarily through fracture during compression. It should be noted that the difference between point 2 and points 1 and 3 arises from the imaging conditions: point 2 was recorded under compression, whereas points 1 and 3 were obtained after unloading, following removal of the indenter.

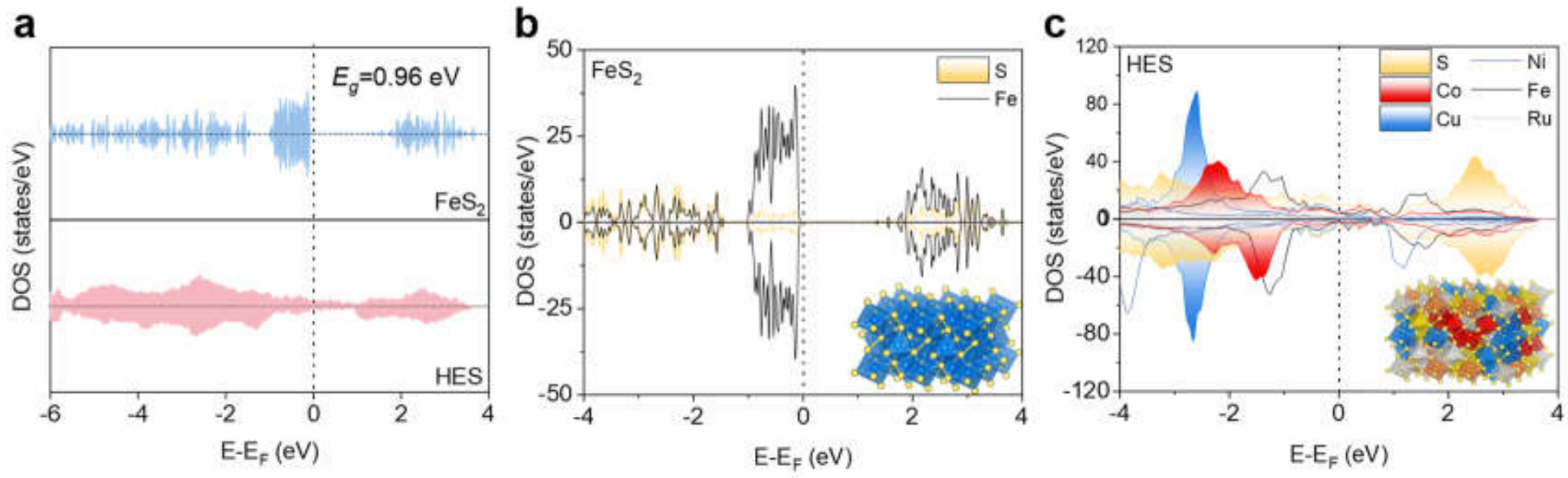


**Fig. S13** (a) Density of states (DOS) of pristine HES and $FeS_2$. (b-c) Structural and pDOS comparison between $FeS_2$ (b) and HES (c).

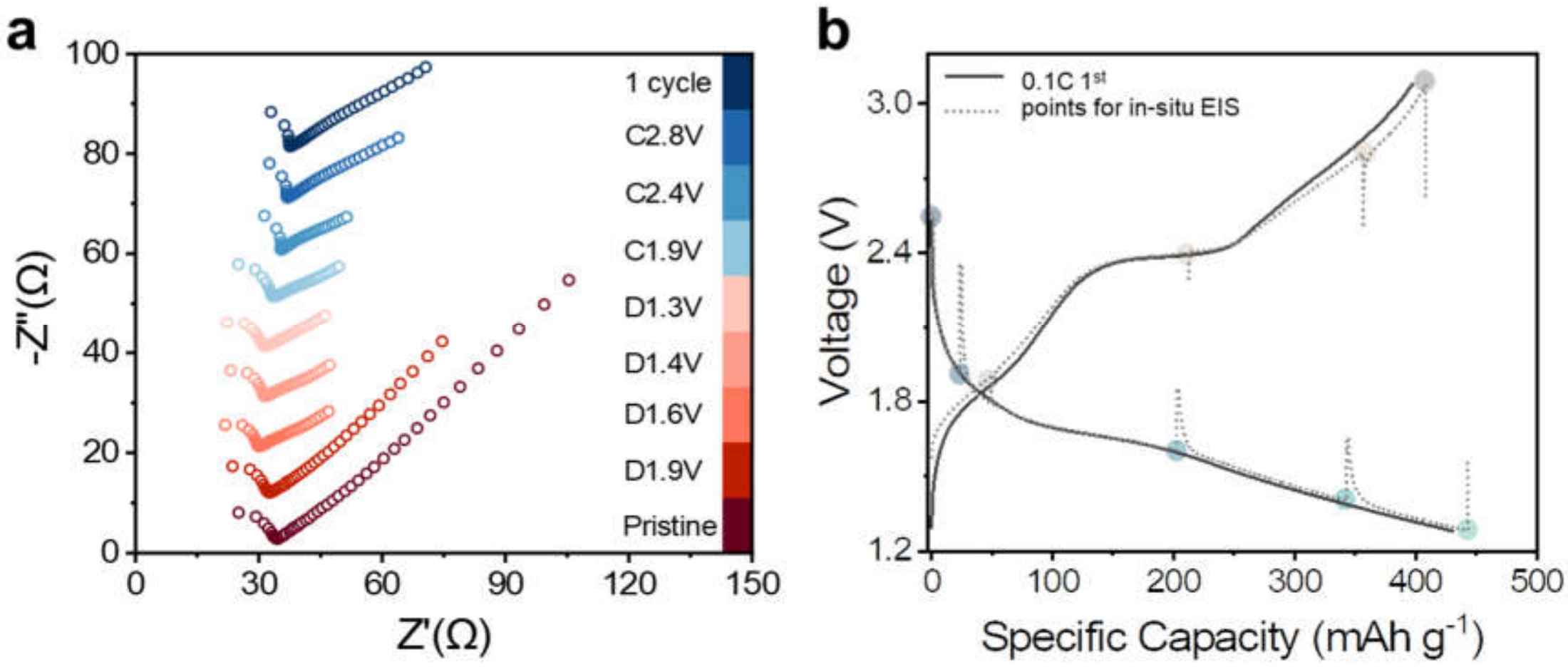


**Fig. S14** In situ EIS analysis of HES. (a) Nyquist plots derived from the in situ EIS measurements. (b) Selected states for in situ EIS measurements of HES, together with the corresponding electrochemical profiles.

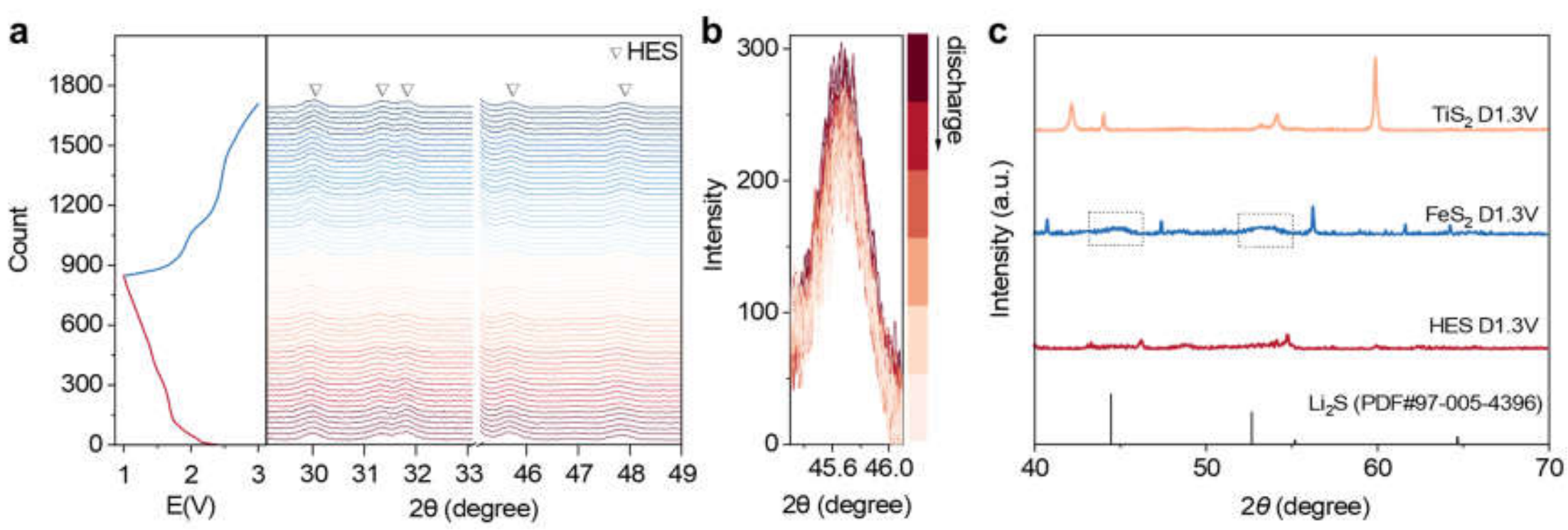


**Fig. S15** In-situ and ex-situ XRD analysis of HES and reference sulfides. (a) In-situ XRD patterns of HES recorded during the first discharge–charge cycle at 0.1 C using a homemade coin-cell–based in situ cell. The probed region experiences negligible

external stack pressure. As a result, incomplete phase conversion of HES may occur due to insufficient pressure. (b) Enlarged view of the main diffraction peak of HES in (a). (c) Ex-situ XRD comparison of HES, $TiS_2$, and $FeS_2$ cathodes at the discharged state (1.3 V vs. $Li^+/Li$), collected from mold cells assembled under an initial stack pressure of 60 MPa applied by bolt fastenin.

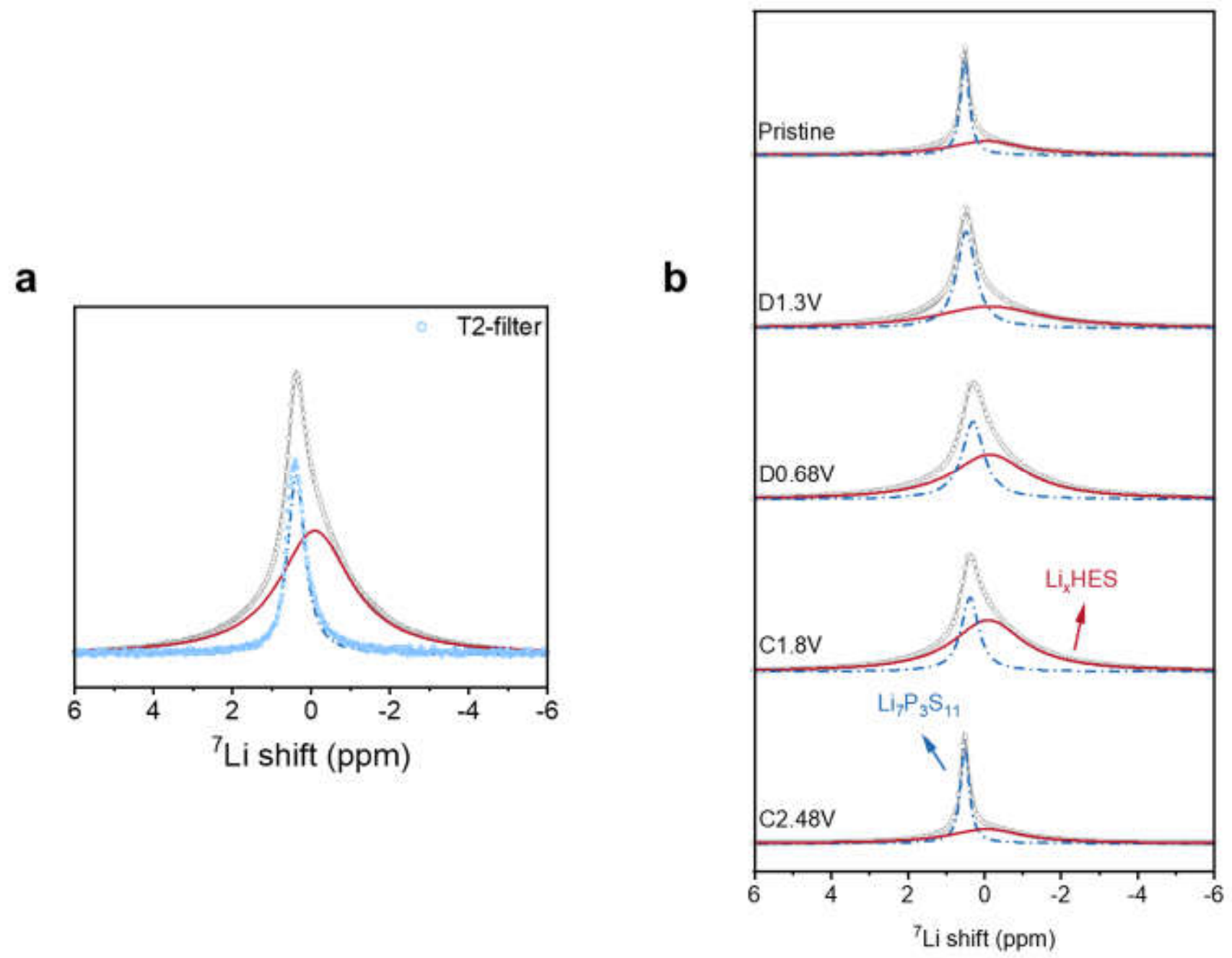


**Fig. S16** $^7$Li ssNMR analysis of lithiated HES cathodes. (a) Schematic illustration of the $T_2$-filter approach, which selectively suppresses the short-$T_2$ $^7$Li signals from the LPS while retaining the longer-$T_2$ components associated with the lithiated HES phase, thereby enabling spectral discrimination between the two contributions. (b) Ex-situ $^7$Li ssNMR spectra of HES cathodes at different discharged and charged states.

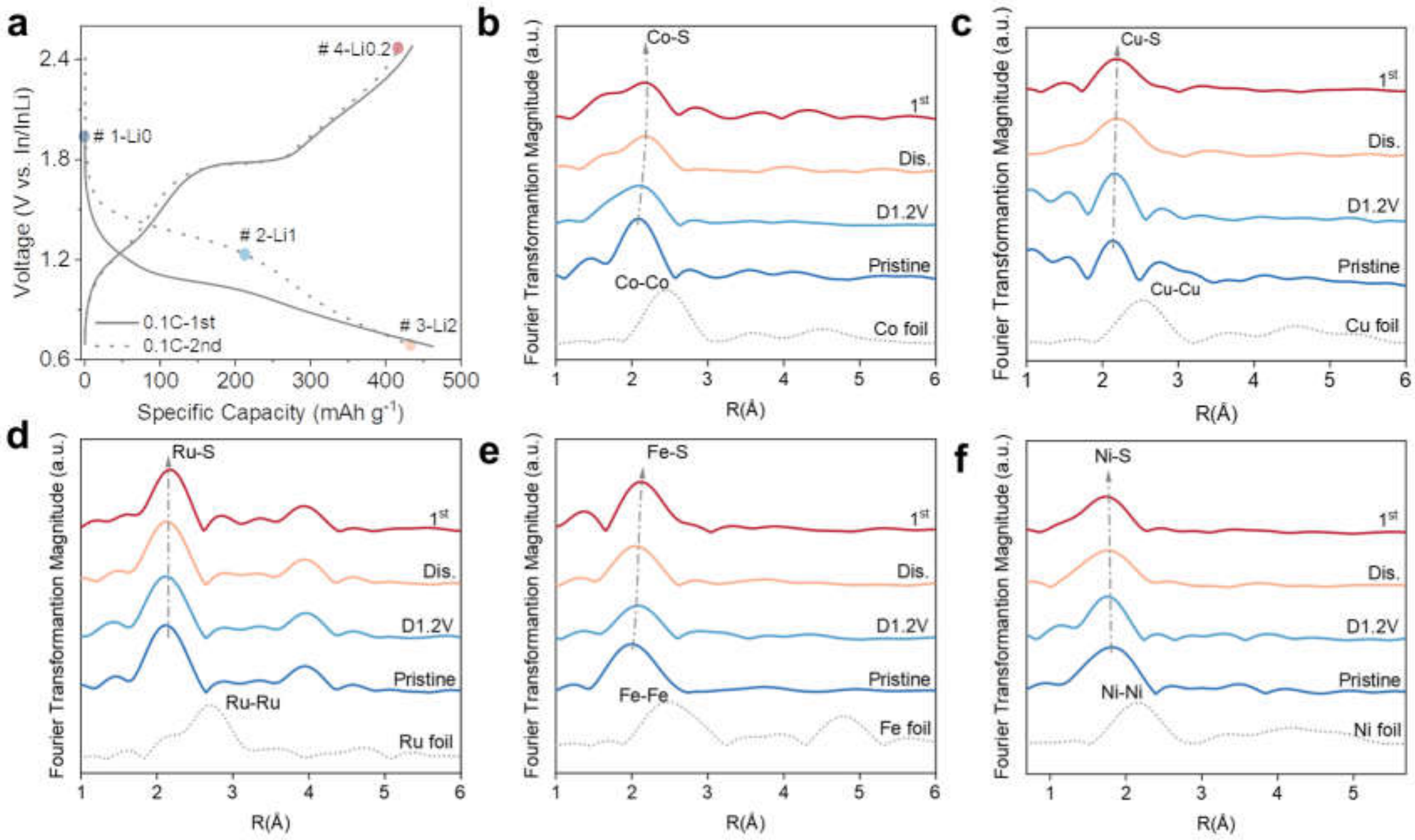


**Fig. S17** Voltage profiles and ex-situ EXAFS analysis of HES during electrochemical cycling. (a) Detailed voltage profiles of ASSLSBs employing HES cathodes cycled at 0.1 C. The lithium content in HES ($Li_x$) is calculated based on the molecular weight of HES ($M_r$=128.27). (b–f) Ex-situ Fourier-transformed EXAFS spectra of Co (b), Cu (c), Ru (d), Fe (e), and Ni (f) collected during the second cycle of a HES|LPSC|Li–In ASSLSB operated at a rate of 0.1 C.

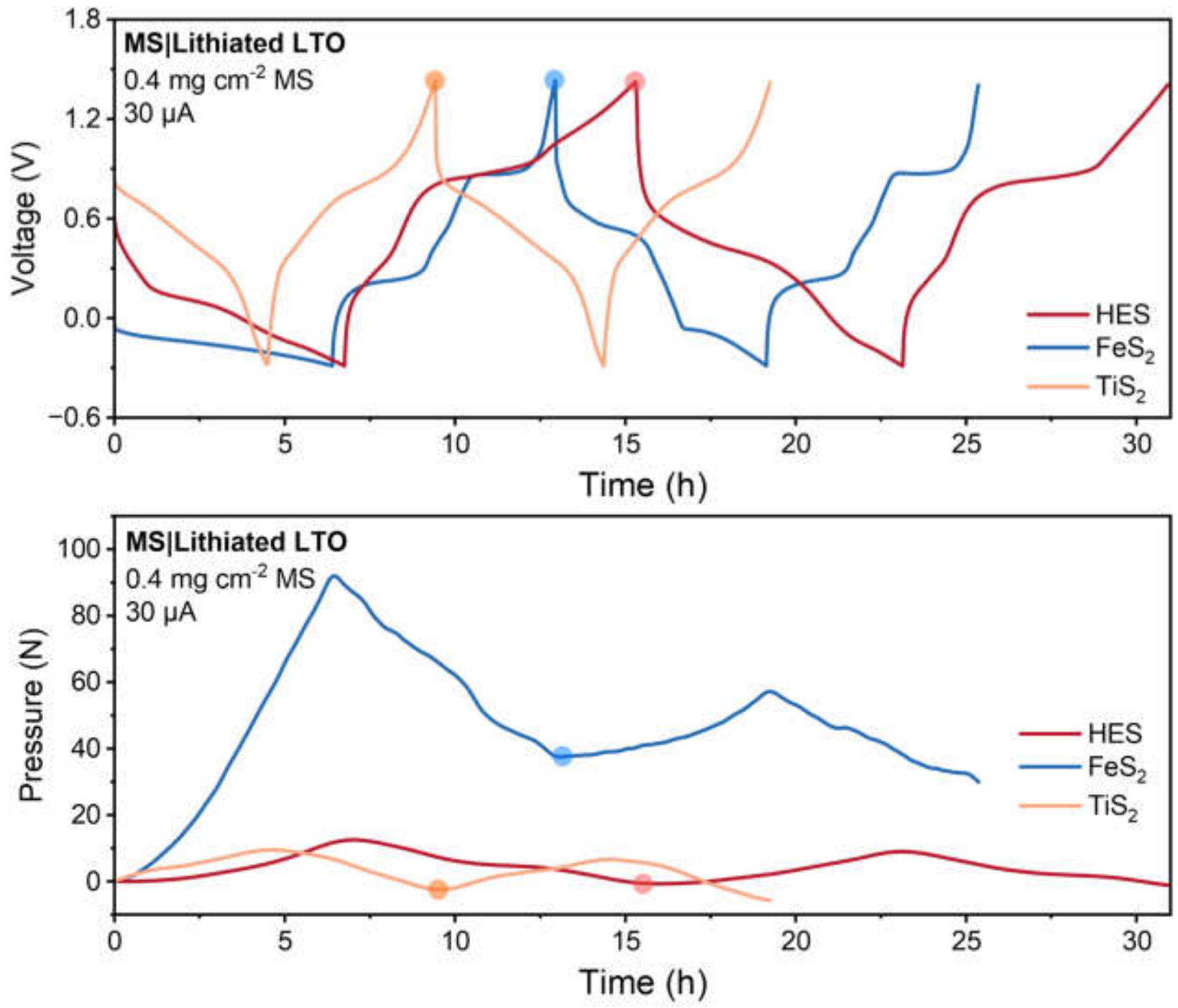


**Fig. S18** Electrochemical cycling behavior and operando pressure response of lithiated LTO|LPSC|MS cells. (a) Galvanostatic cycling performance. (b) Corresponding operando pressure evolution during electrochemical cycling.

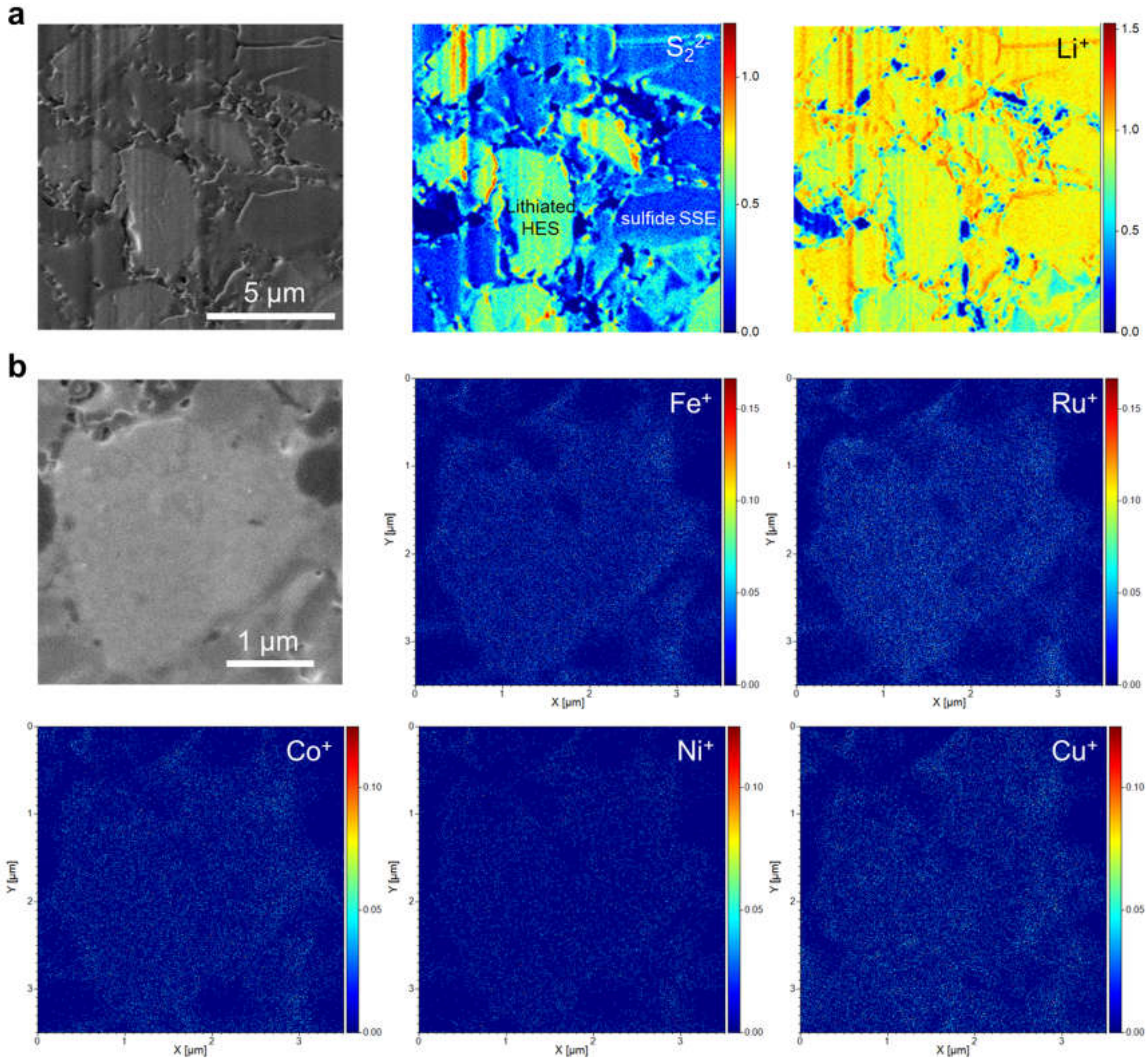


**Fig. S19** ToF-SIMS analysis of discharged HES composite cathode. (a-b) Two representative regions of the discharged HES particles were selected for ToF-SIMS analysis. (a) Planar distributions of $S_2^{2-}$ (m/z = 32) and $Li^+$ (m/z = 7) fragments. The homogeneous distributions indicate uniform lithiation throughout the HES bulk while preserving the integrity of the anionic framework, with no evident structural disruption upon discharge. Owing to the intrinsically destructive nature of ToF-SIMS measurements, a separate region (b) was selected for imaging of low-abundance transition-metal fragments, including $Fe^+$ (m/z = 56), $Ru^+$ (m/z = 102), $Co^+$ (m/z = 59), $Ni^+$ (m/z = 58), and $Cu^+$ (m/z = 63). The resulting distributions reveal no discernible transitional elemental segregation within the probed region.

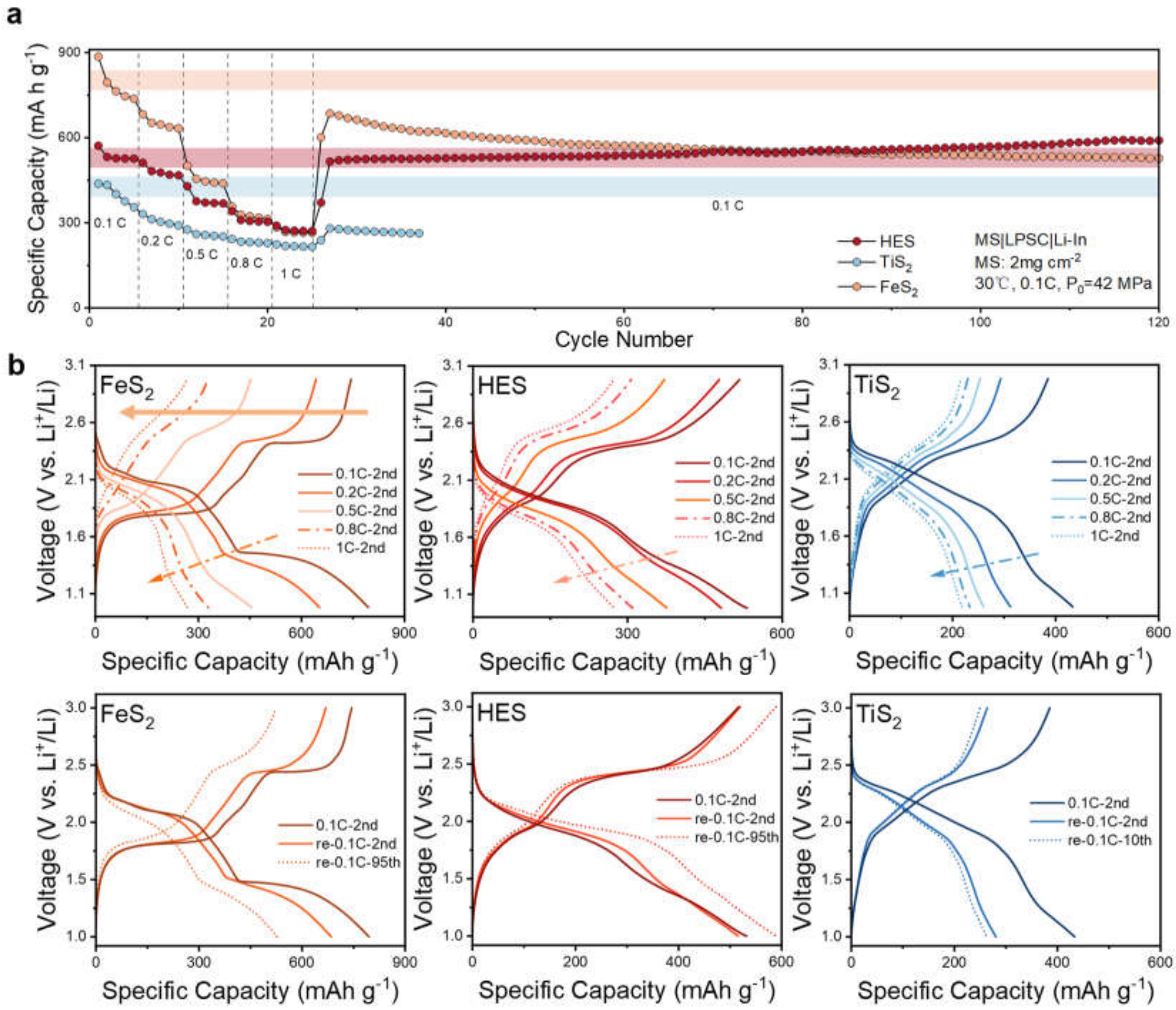


**Fig. S20** Electrochemical performance comparison between ASSLSBs employing HES, $FeS_2$ and $TiS_2$ cathodes. (a) Rate performance. (b) Representative discharge–charge voltage profiles at different cycle numbers. The cycling voltage window is intentionally extended beyond the typical 1-3 V vs. $Li^+/Li$ range commonly adopted for transitional metal sulfide cathodes.

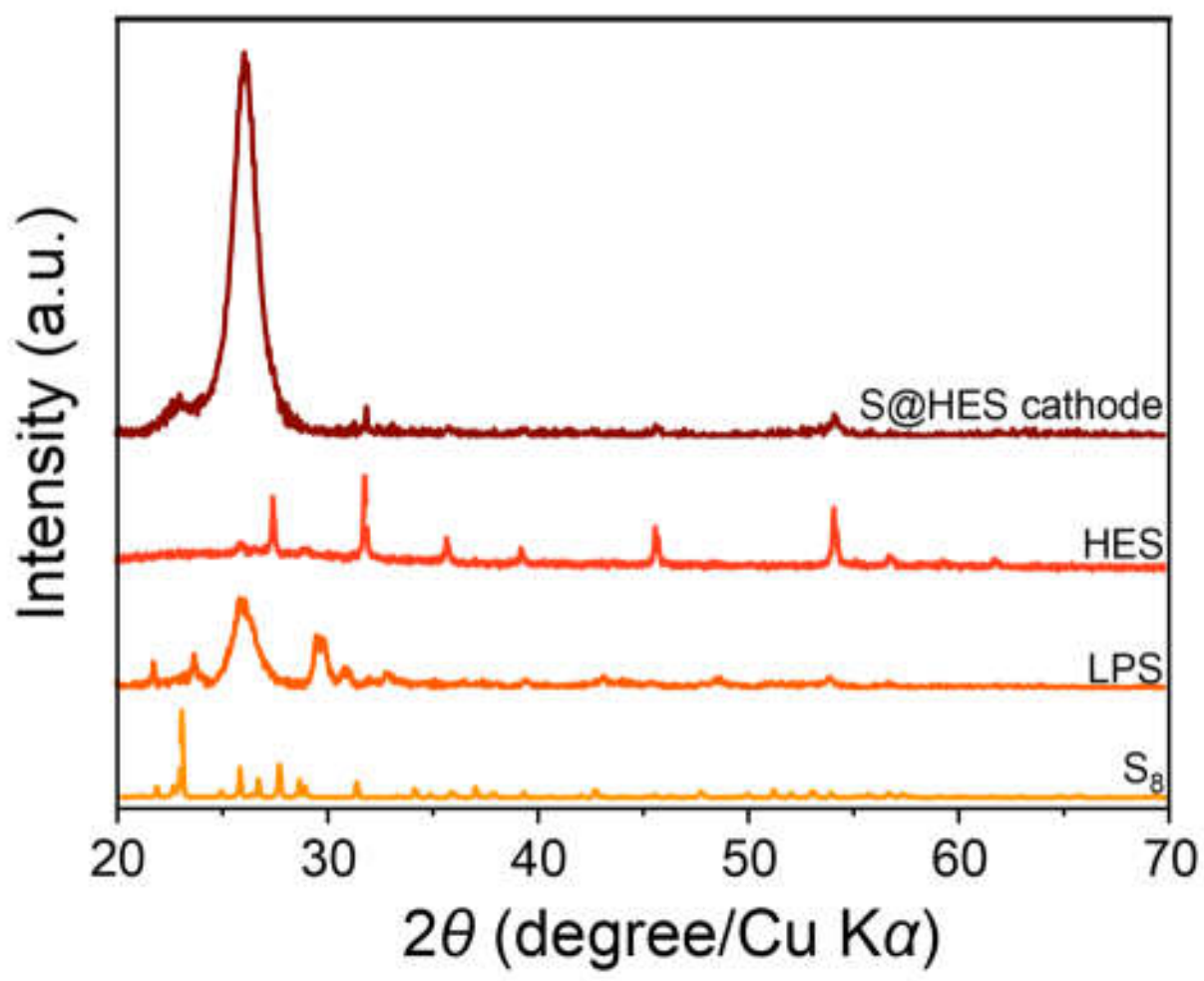


**Fig. S21** XRD pattern of S@HES, showing that HES retains distinct diffraction signals even after high-energy ball milling.

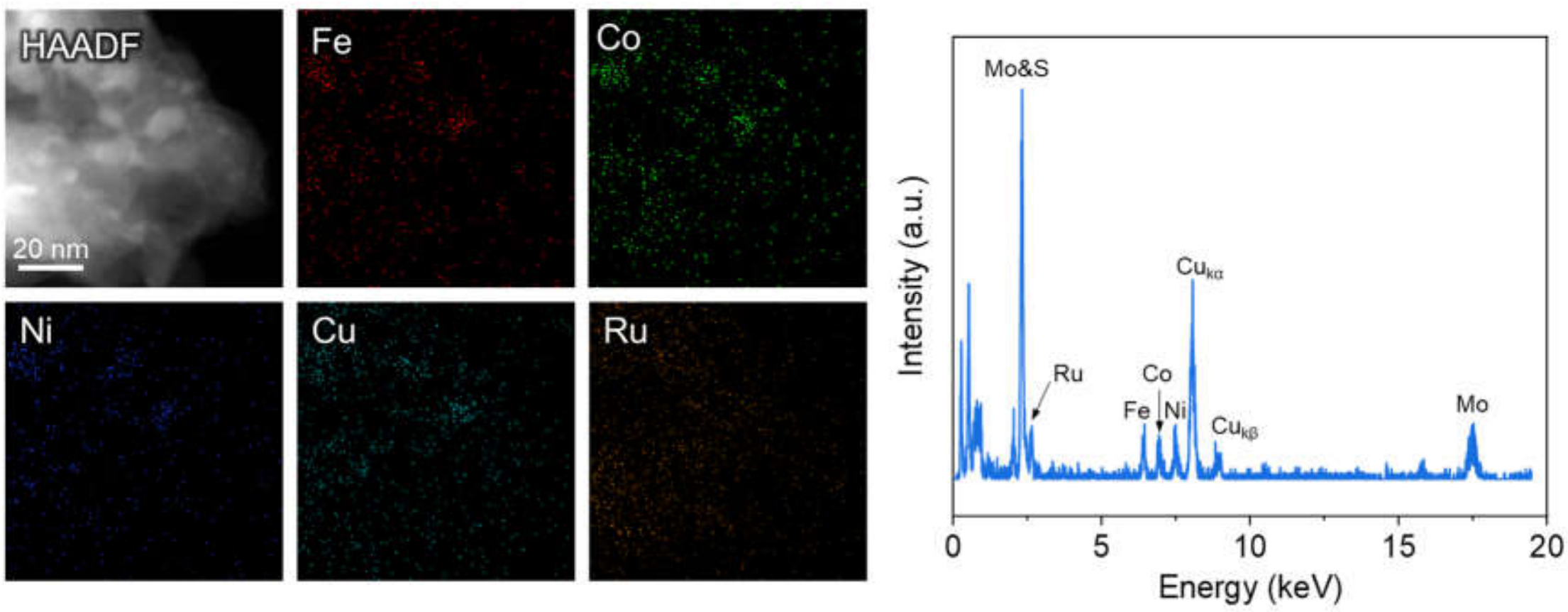


**Fig. S22** HAADF-STEM image of S@HES and the corresponding EDS elemental maps of Fe, Co, Ni, Cu, and Ru, together with the EDS spectrum.

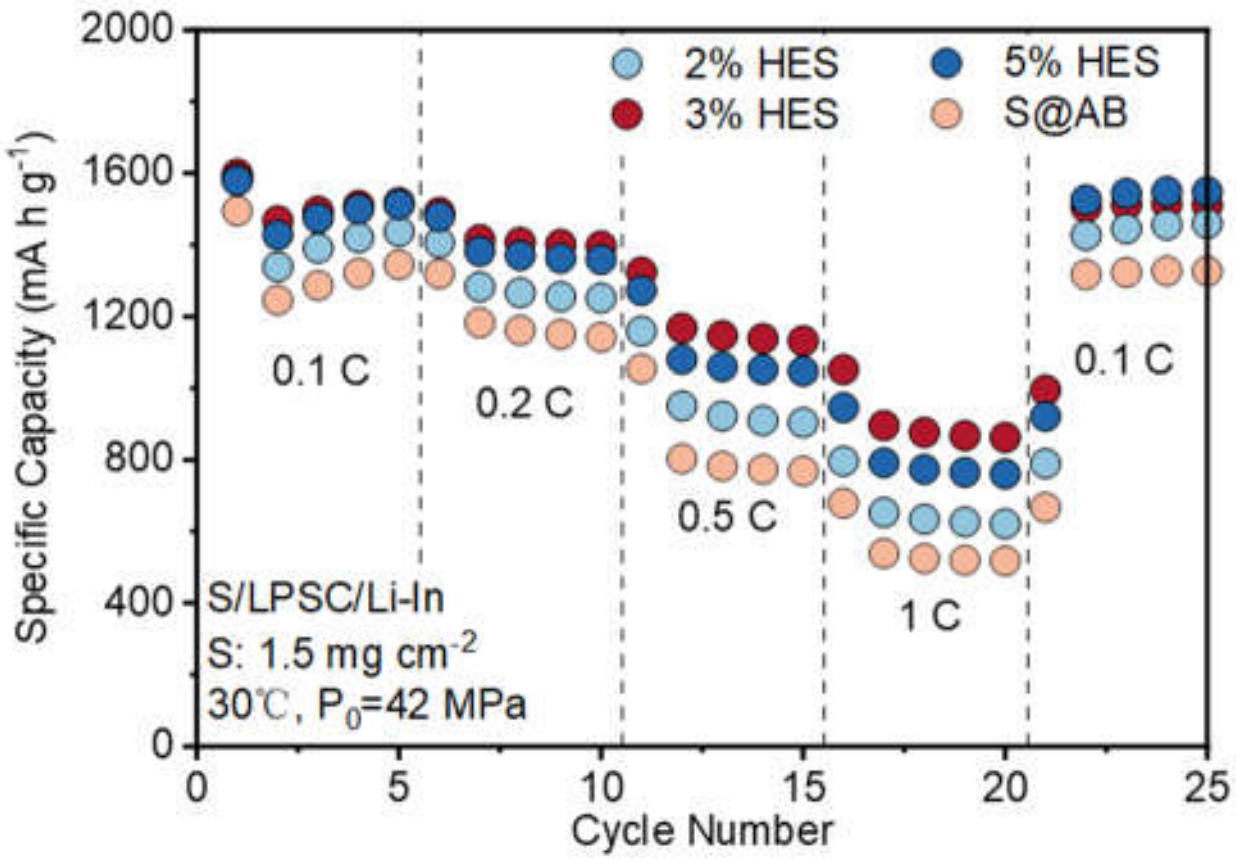


**Fig. S23** Electrochemical performance of S@HES cathodes with different amounts of AB partially replaced by HES.

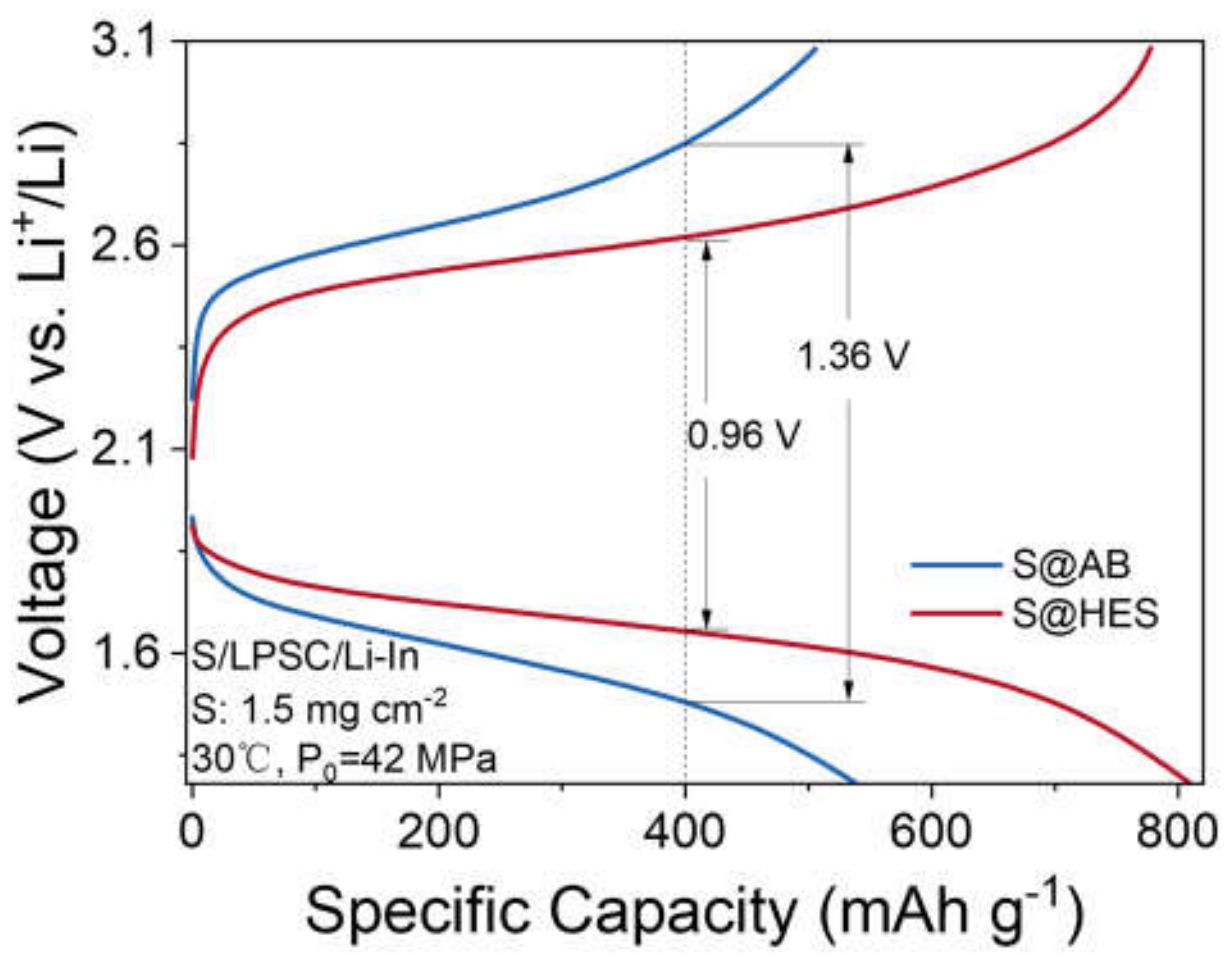


**Fig. S24** Detailed discharge-charge curves of the ASSLSBs with S@AB and S@HES at 1C.

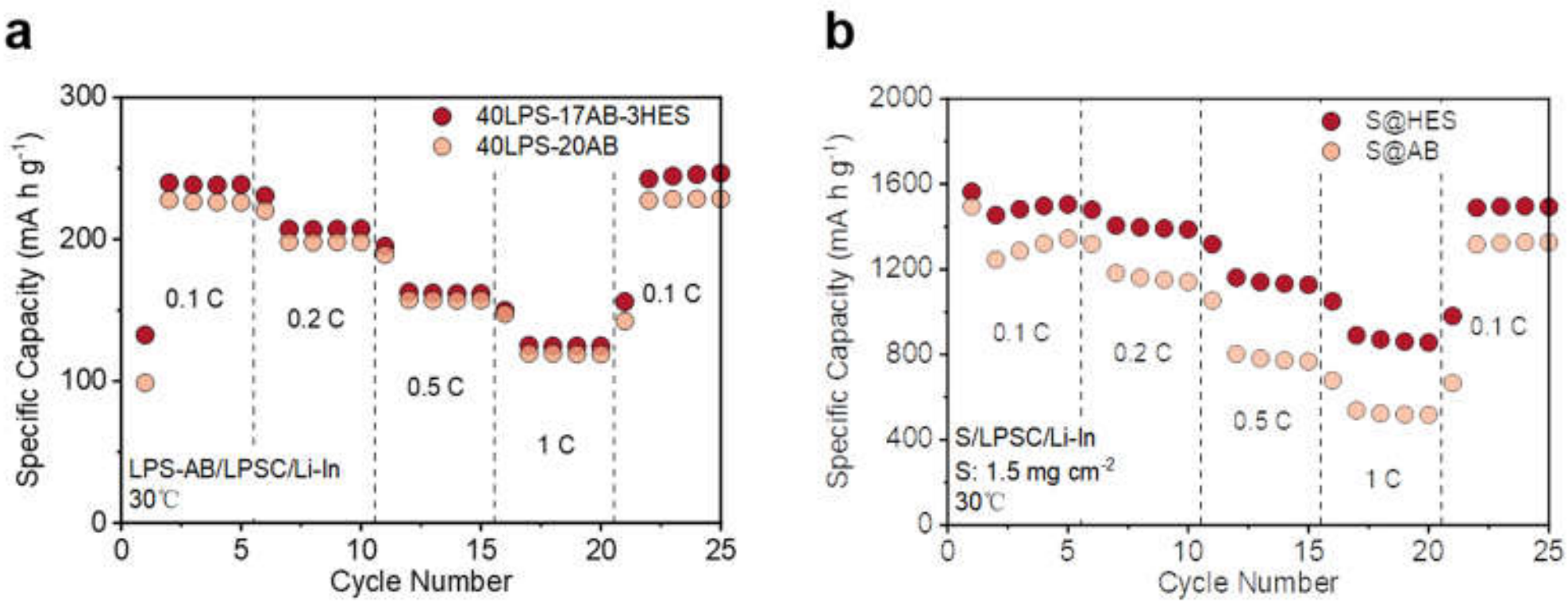


**Fig. S25** Influence of LPS on capacity. (a) Capacity contribution from the LPS and HES (with current density calculated based on the sulfur corresponding to LPS-AB). (b) Rate performance after subtracting the HES contribution.

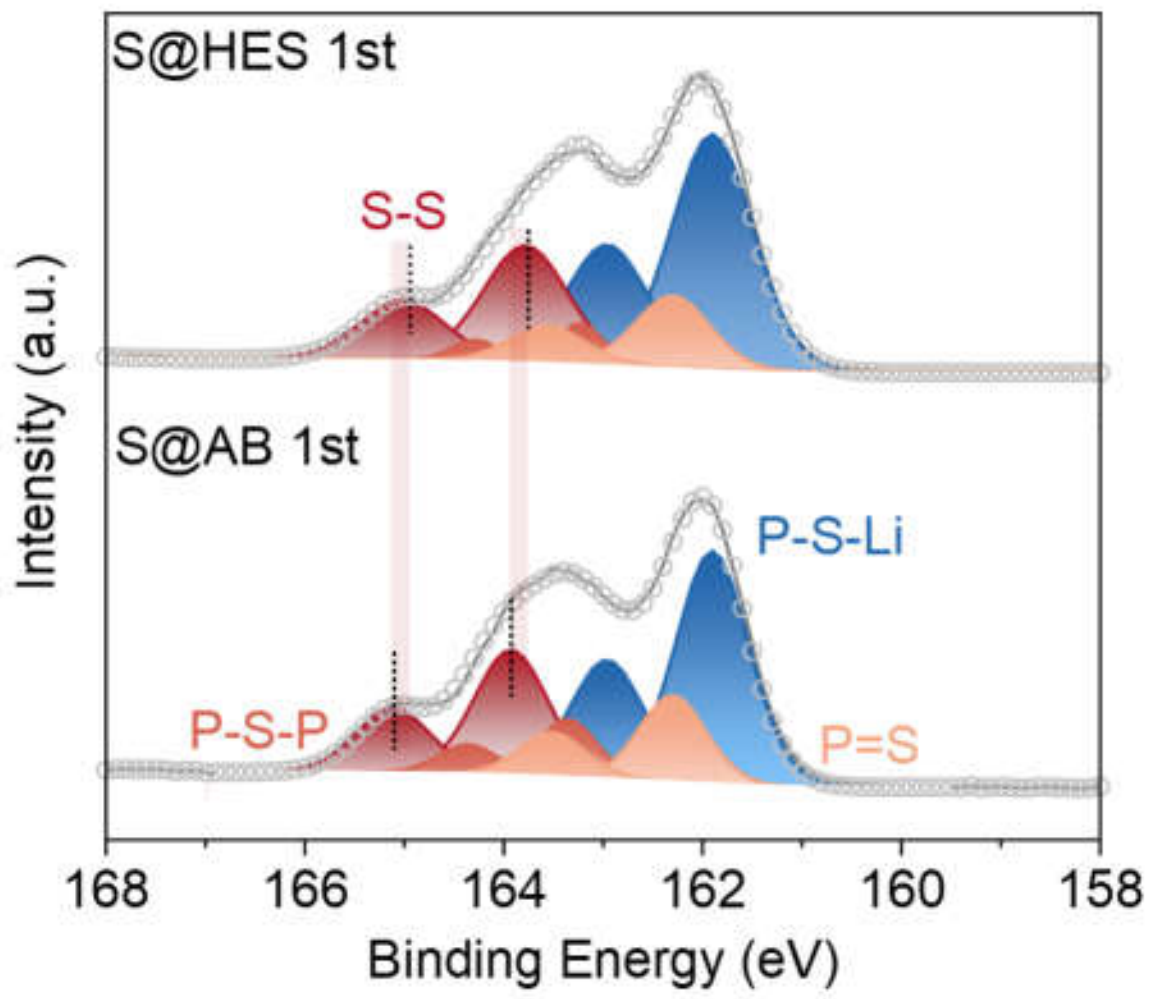


**Fig. S26** S 2p XPS spectra of S@AB and S@HES cathodes after one cycle at 0.1C.

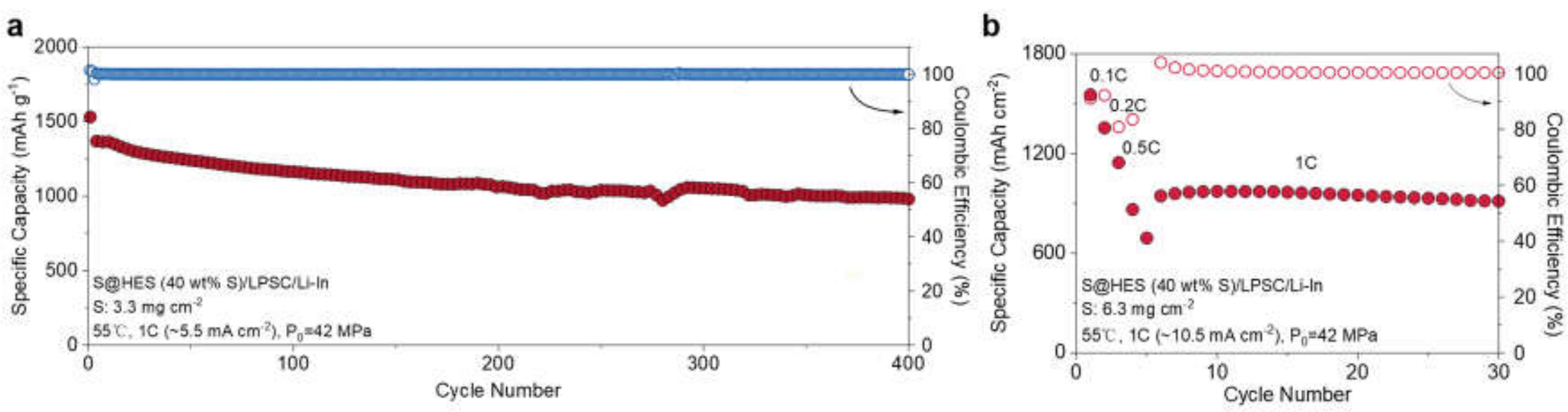


**Fig. S27** Cycling performance of S@HES cathodes with 40 wt% sulfur content under high-rate conditions at 55°C. (a) S loading of 3.3 mg cm$^{-2}$. (b) S loading of 6.3 mg cm$^{-2}$.

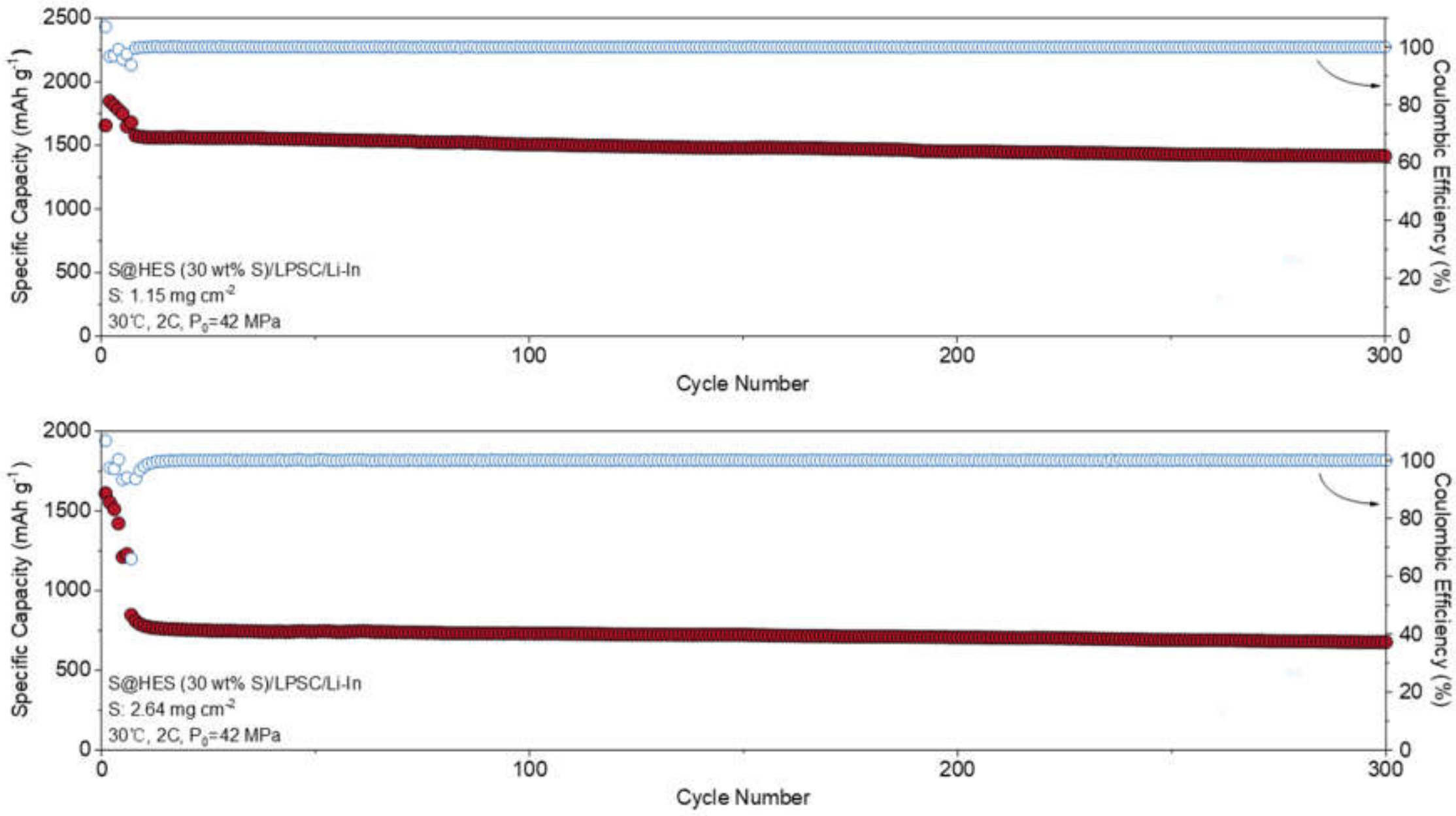


**Fig. S28** High-rate (2C) long-term cycling performance of S@HES cathodes with low sulfur content (30 wt%) at room temperature.

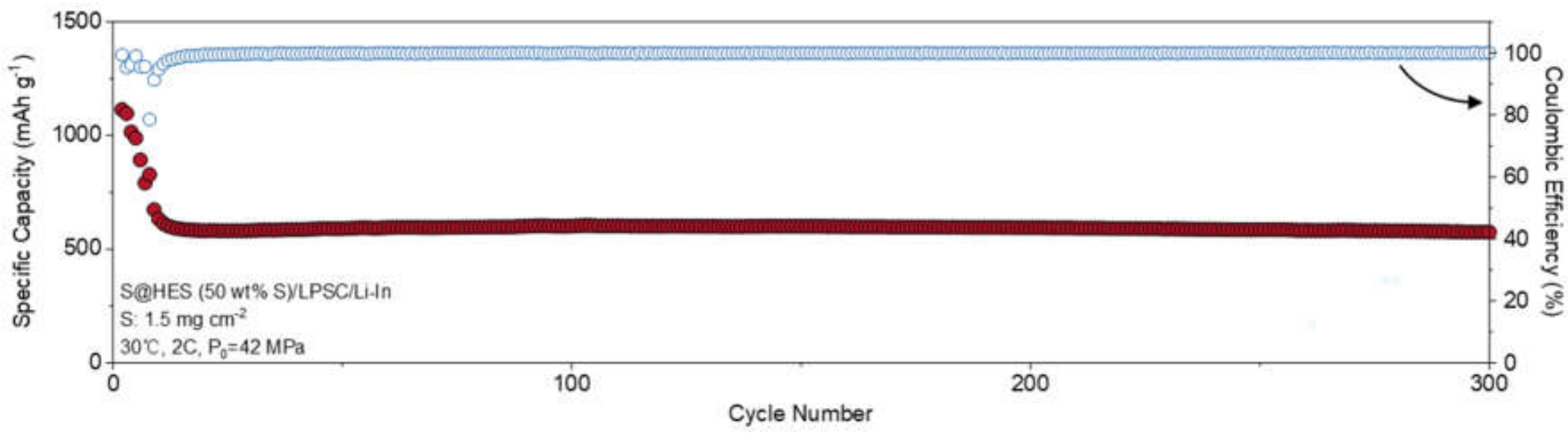


**Fig. S29** High-rate (2C) long-term cycling performance of S@HES cathodes with low sulfur content (50 wt%).

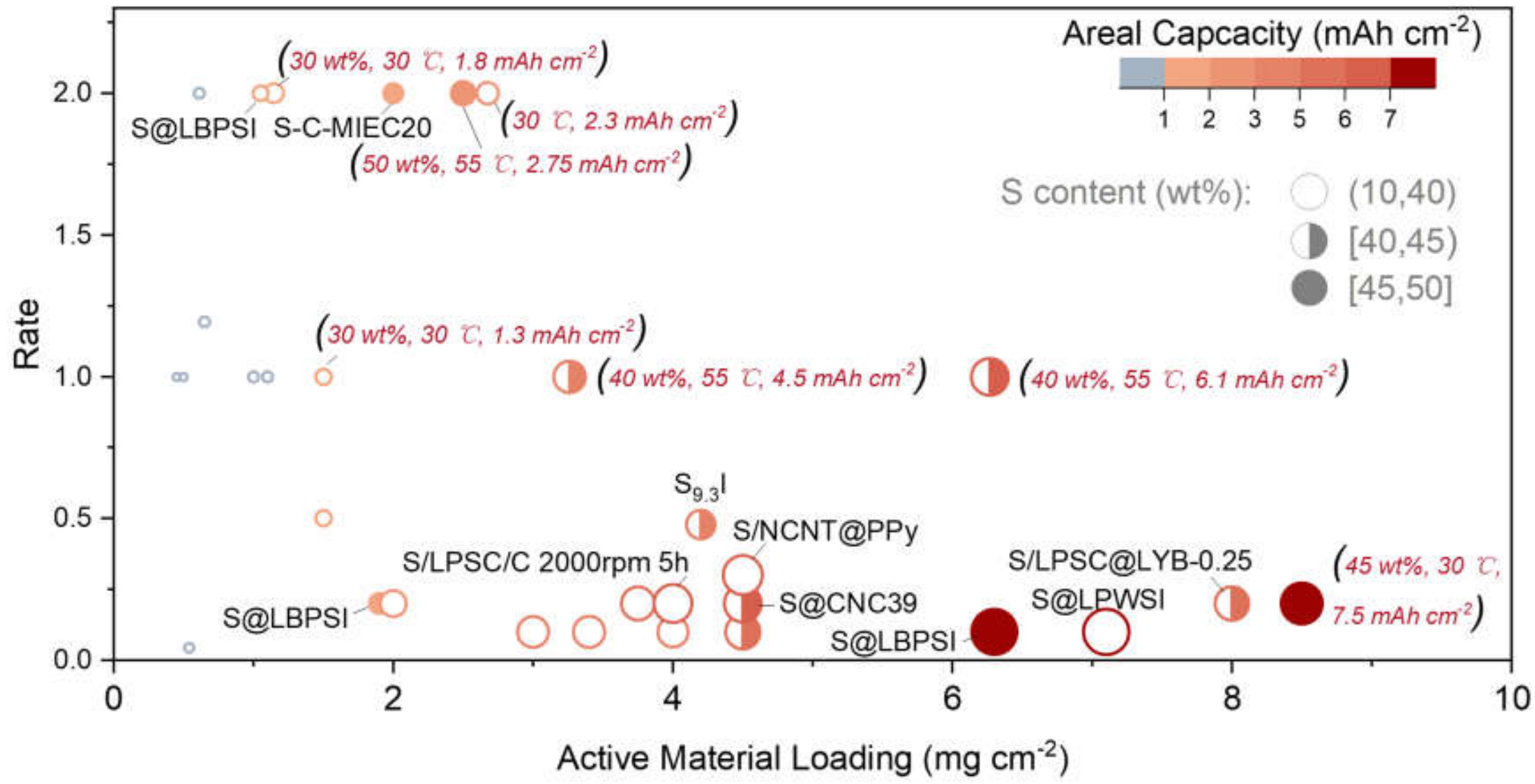


**Fig. S30** Bubble chart comparing active material loading, rate capability, and areal capacity between this work and previously reported studies.

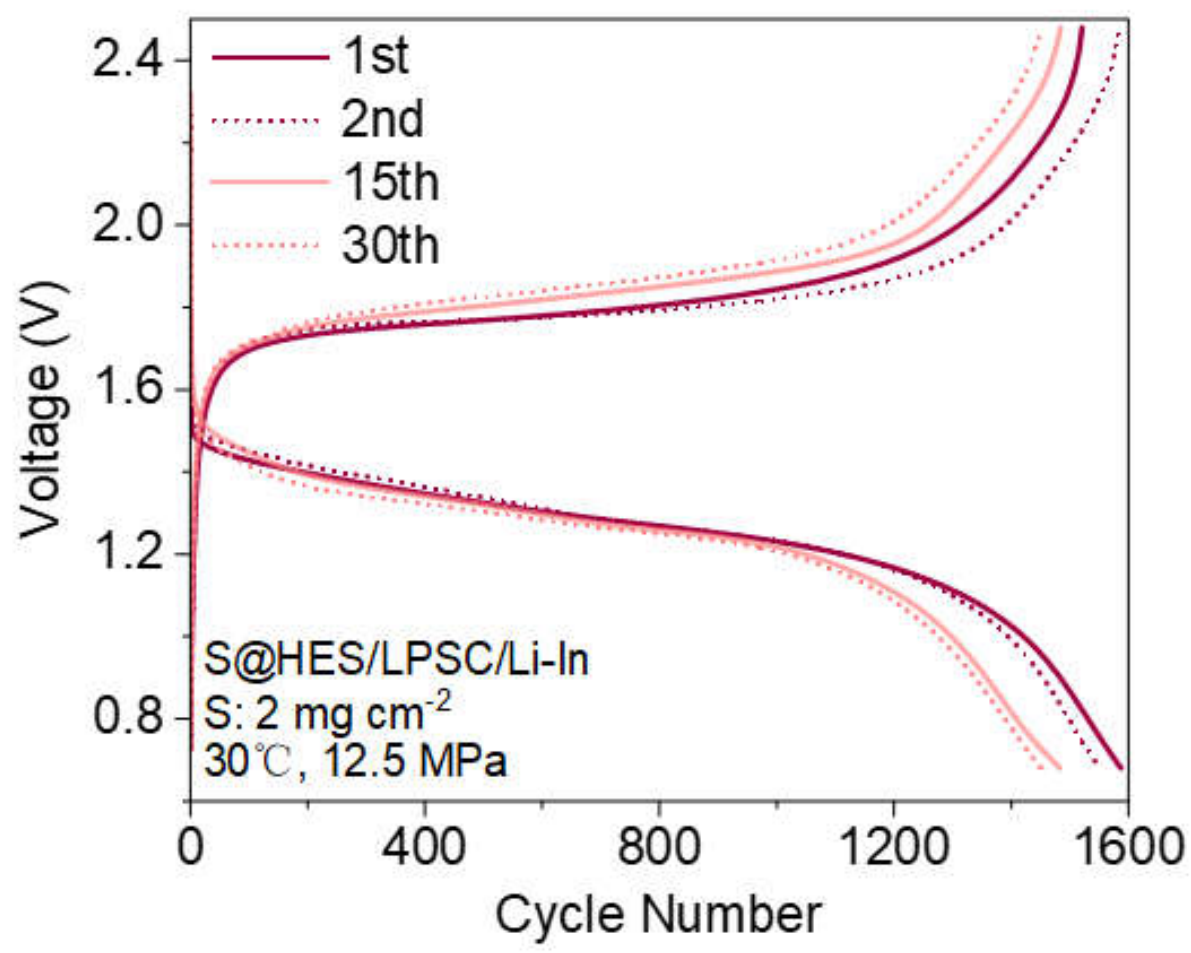


**Fig. S31** Discharge–charge voltage profiles of ASSLSBs employing S@HES cathodes cycled at 0.1 C under a stack pressure of 12.5 MPa.

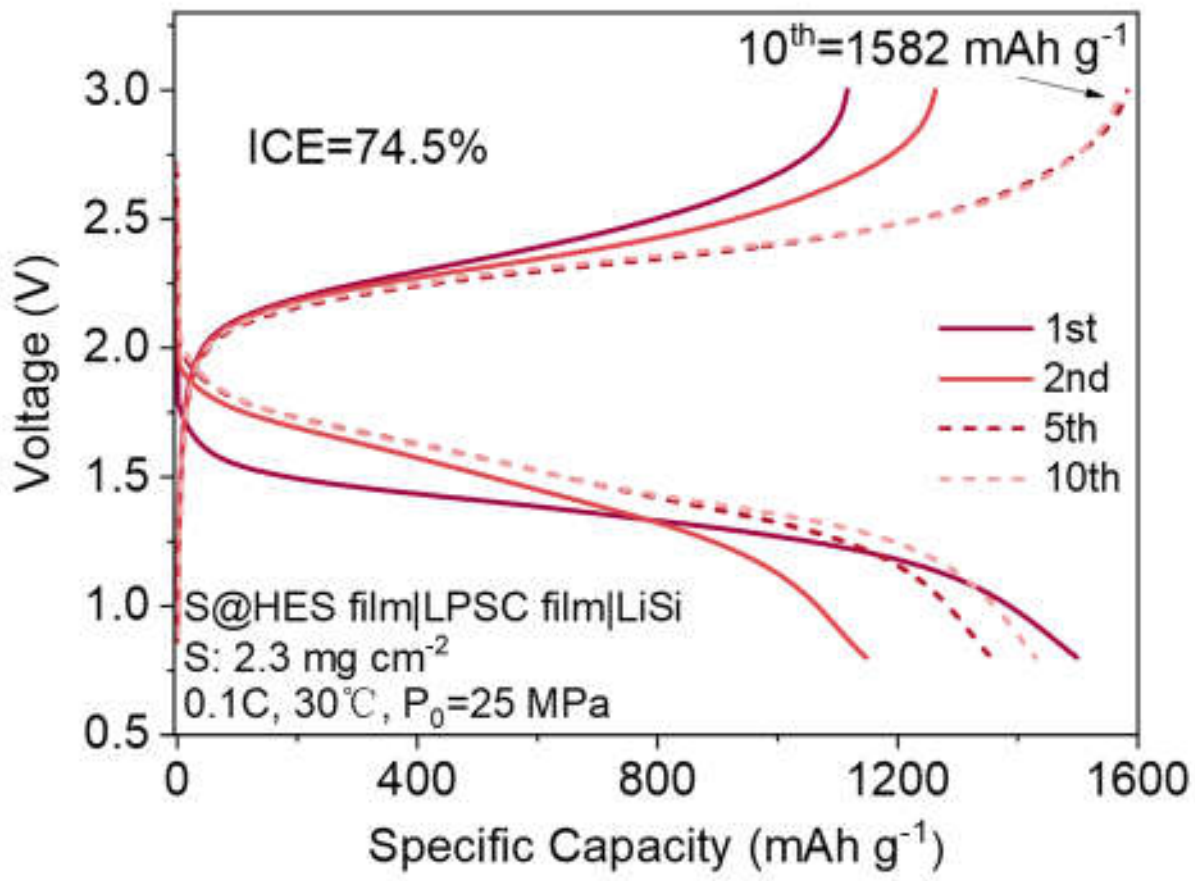


**Fig. S32** Voltage profiles of S@HES cells assembled using a bilayer LPSC electrolyte-film and a $Li_{3.75}Si$ alloy anode.

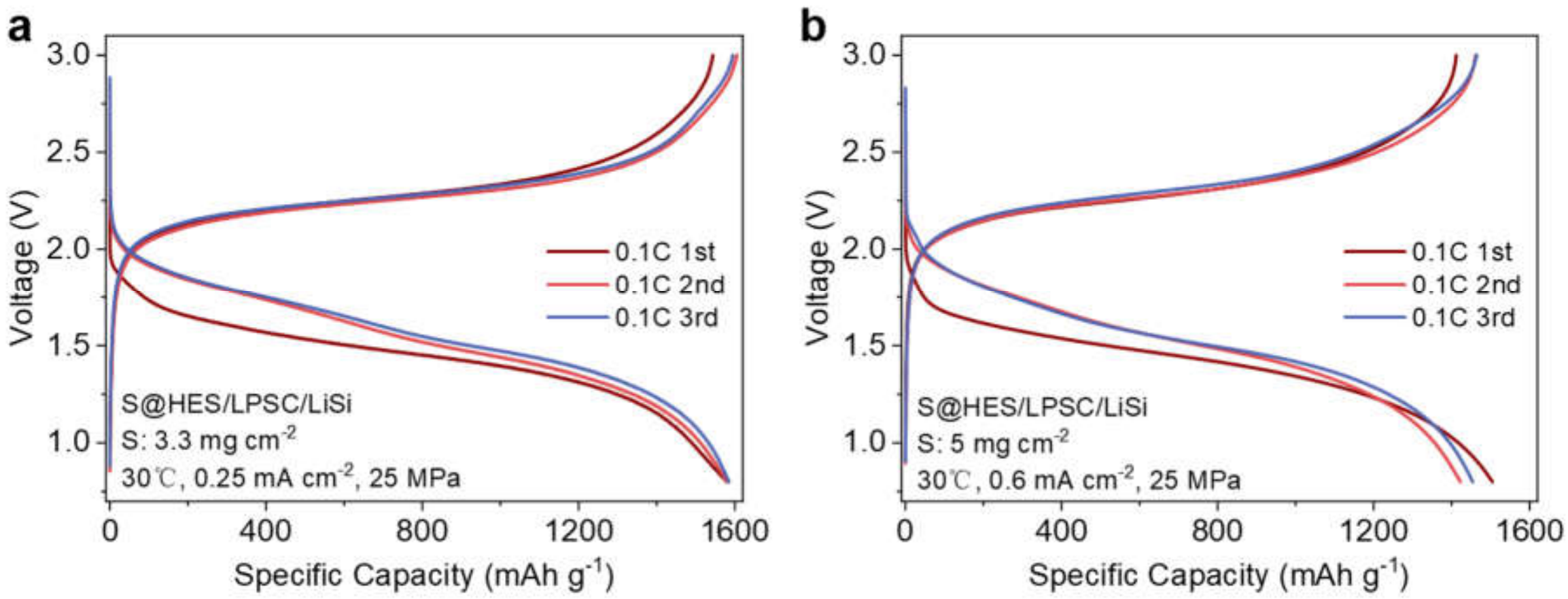


**Fig. S33** Discharge-charge voltage profiles of S@HES|$Li_{3.75}Si$ cells at an N/P ratio of 1.6 with different S loadings. (a) 3.3 mg $cm^{-2}$; (b) 5.0 mg $cm^{-2}$.

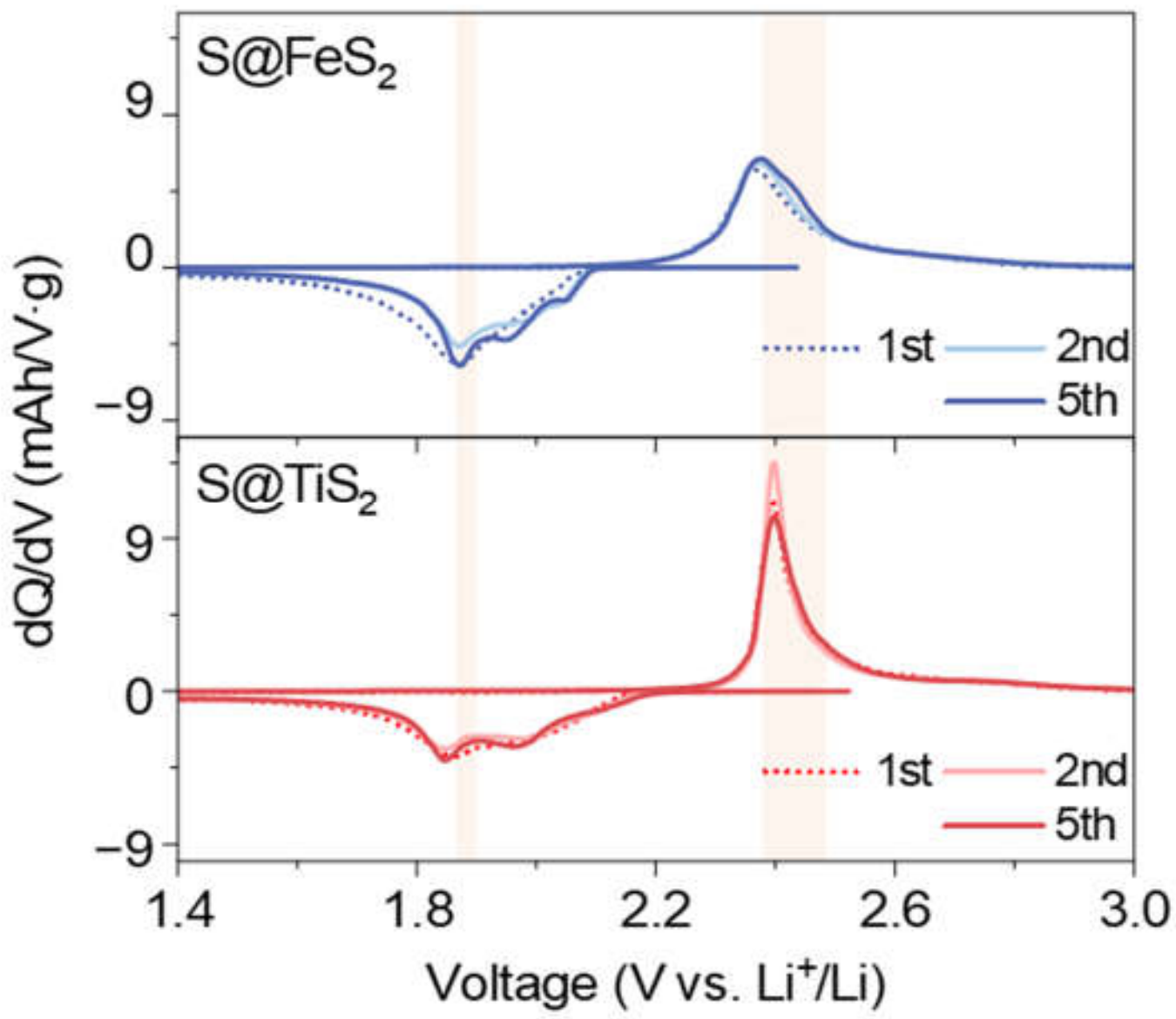


**Fig. S34** dQ/dV curves of S@FeS$_2$ and S@TiS$_2$ cathodes at selected cycles.

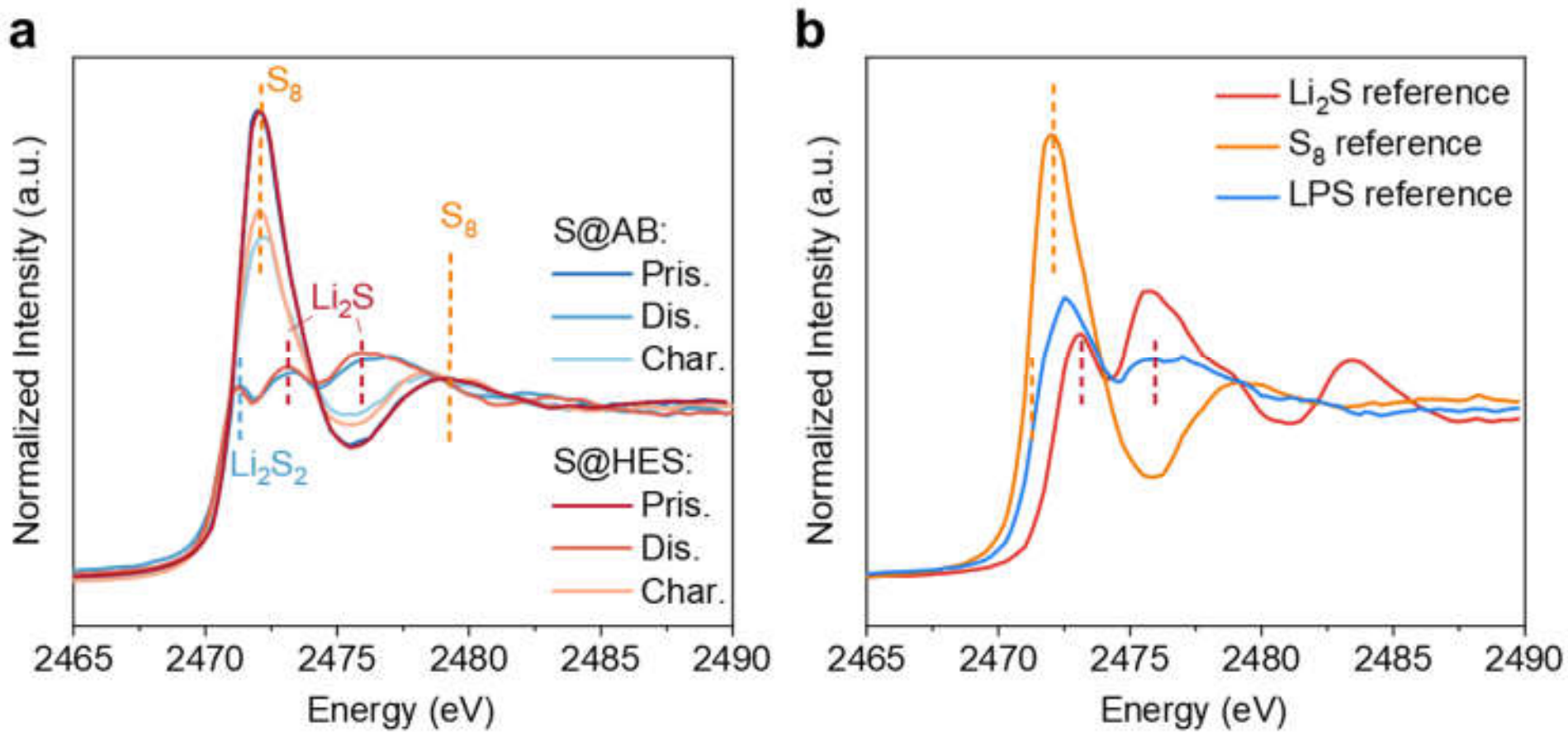


**Fig. S35** Ex-situ S K-edge XANES analysis of sulfur speciation during cycling. (a) S K-edge XANES spectra of S@HES and S@AB cathode collected at pristine, discharged (1.3V vs. $Li^+$/Li) and charged (3.1V vs. $Li^+$/Li) states. (b) Reference spectra of $Li_2S$, S and LPS.

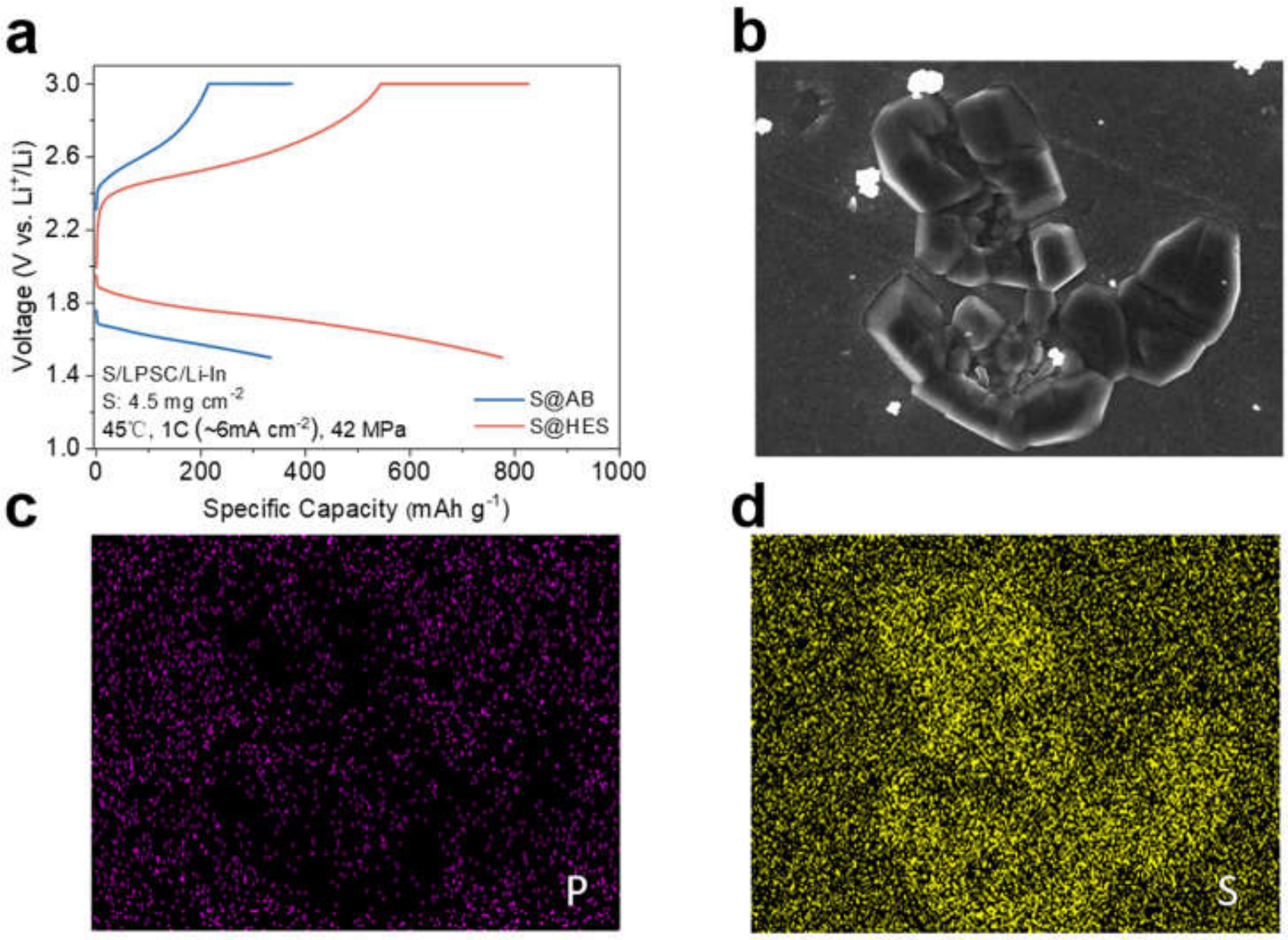


**Fig. S36** Electrochemical behavior and post-cycling morphology after the constant-current–constant-voltage (CC–CV) test. (a) Electrochemical voltage profiles of ASSLSBs employing S@HES and S@AB cathodes under the CC–CV protocol. (b) Morphology of crystalline sulfur observed in the composite cathode after the CC–CV test. (c-d) Corresponding elemental maps acquired from the region shown in (b).

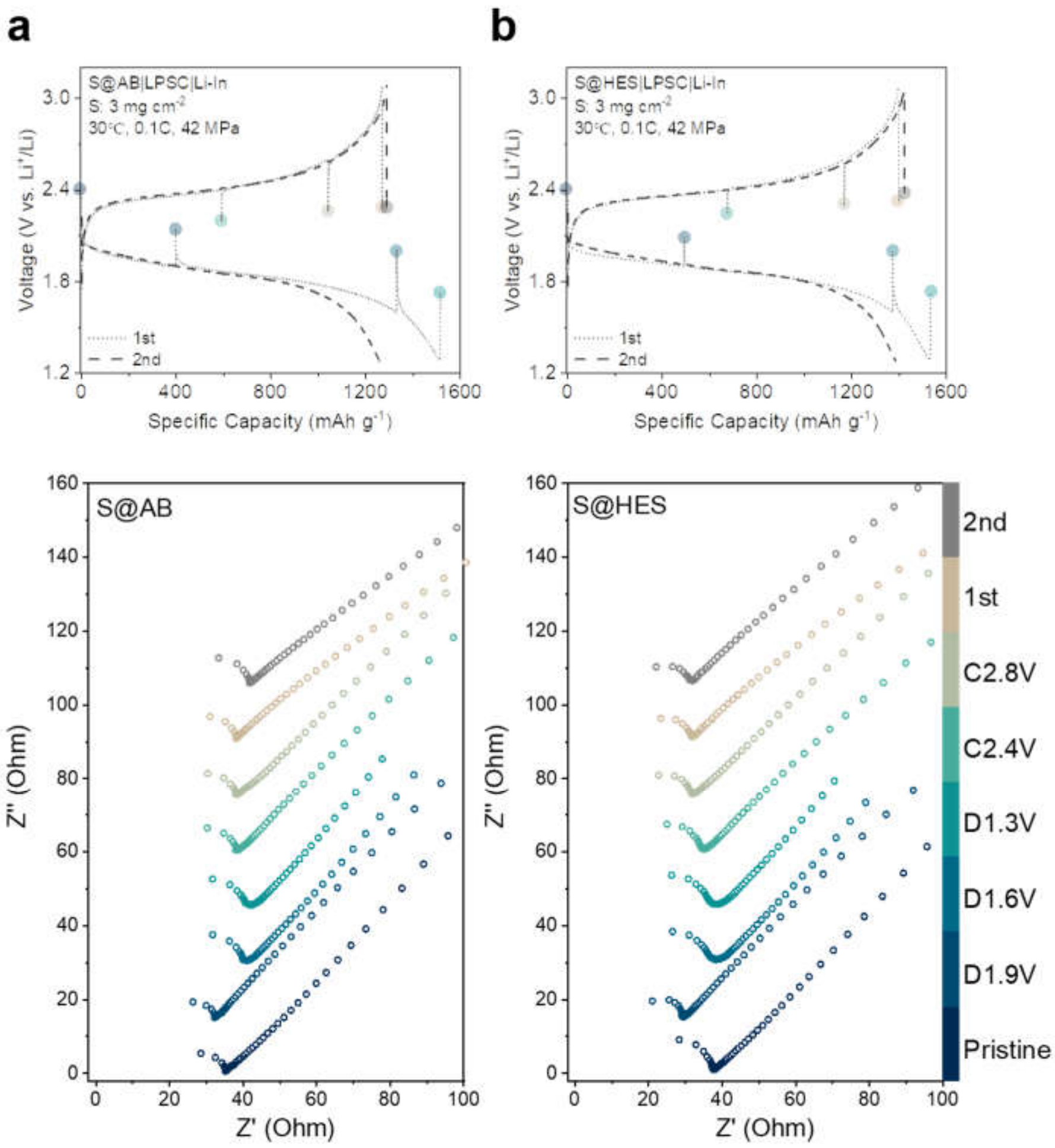


**Fig. S37** In-situ EIS spectra and corresponding voltage curves of ASSLSBs with (a) S@AB and (b) S@HES at a rate of 0.1C.

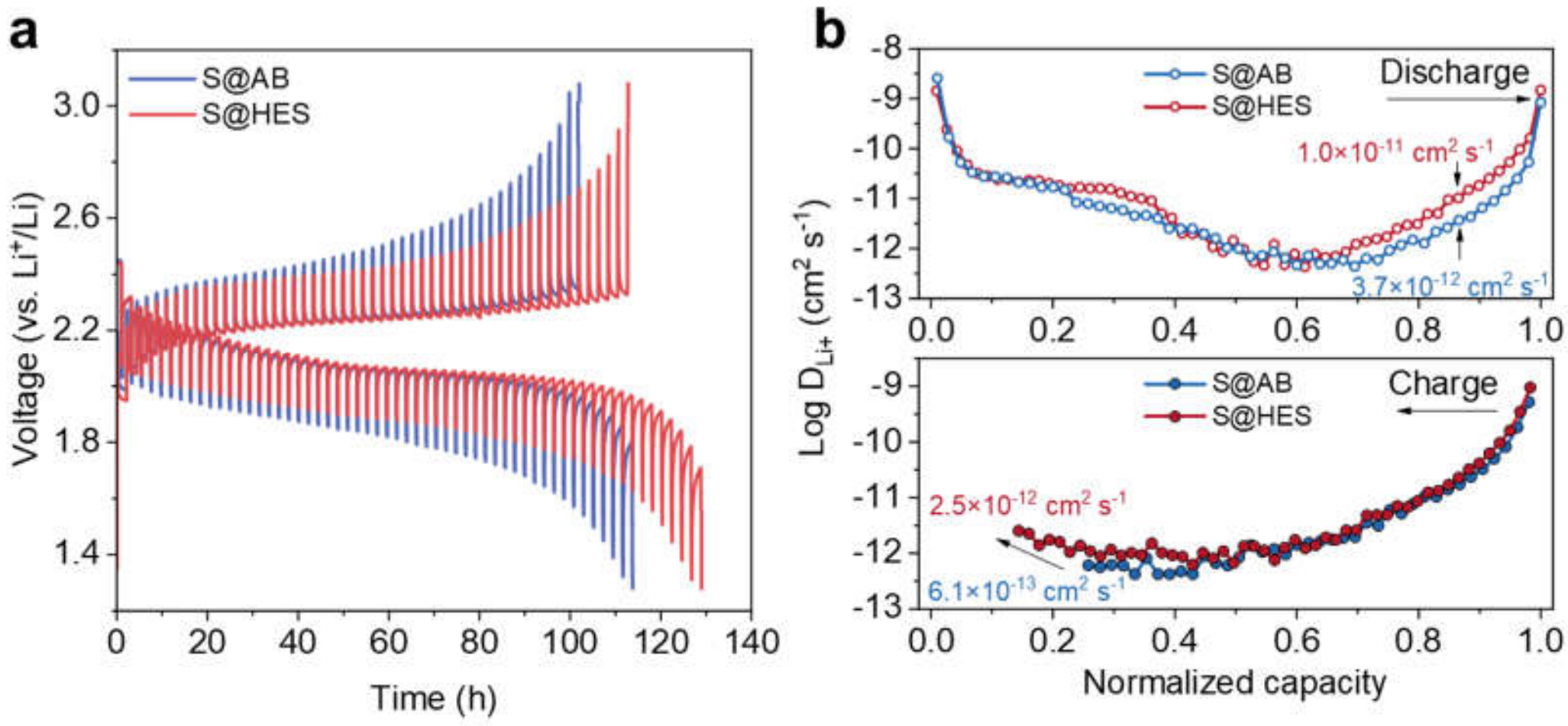


**Fig. S38** Comparison of (a) GITT profiles and corresponding (b) Li-ion diffusion coefficients of S@AB and S@HES composite cathodes calculated from GITT curves.

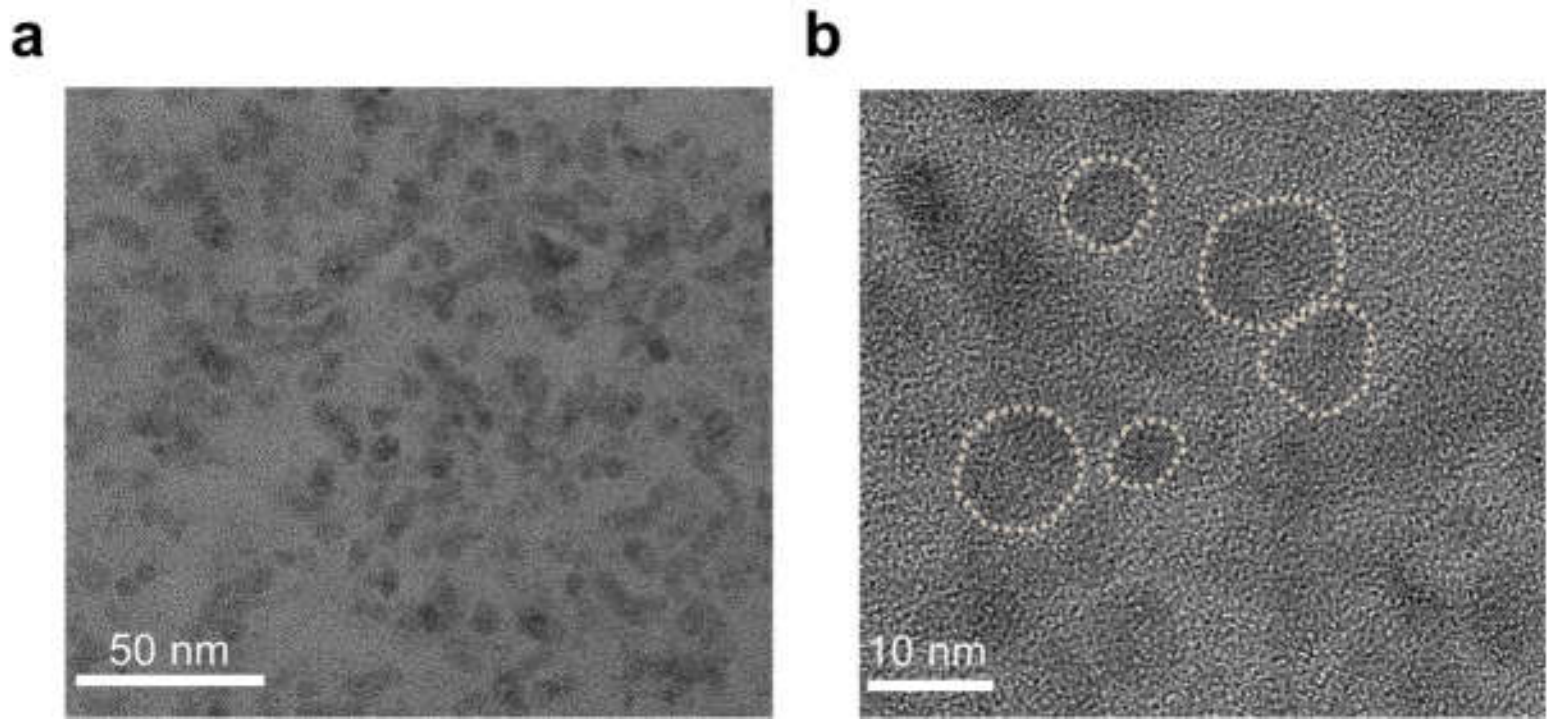


**Fig. S39** Structural characterization of HES after discharge to 1.3 V vs. $Li^+$/Li. (a) Low-magnification TEM image showing uniformly distributed nanosized HES particles over a large field of view. (b) HR-TEM image revealing nanoscale domains.

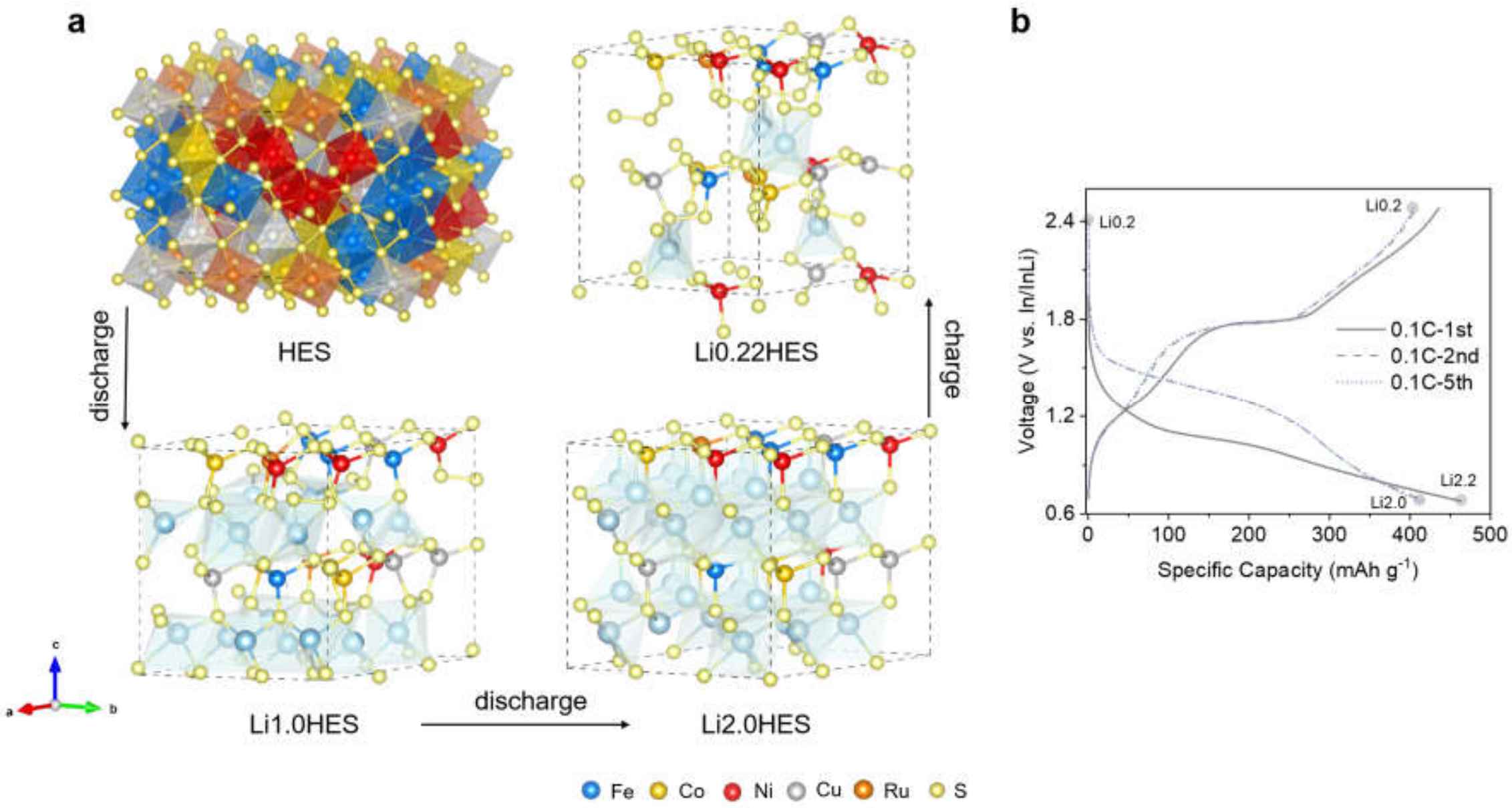


**Fig. S40** (a) Relaxed crystal structures of HES, $Li_{0.22}$HES, $Li_{1.0}$HES, and $Li_{2.0}$HES, and a schematic representation of their structural evolution upon lithiation. (b) Discharge–charge profiles of the HES cathode and the corresponding Li content in $Li_x$HES. Please note that $Li_{0.22}$HES is a coulometry-derived average composition and a representative DFT model, rather than a phase directly determined by structural refinement. Based on the experimental composition of $Fe_{0.23}Co_{0.21}Ni_{0.24}Cu_{0.2}Ru_{0.12}S_2$, the molecular weight of HES is 128.27 g $mol^{-1}$, corresponding to a theoretical capacity of ~209 mAh $g^{-1}$ for one Li per HES formula unit. The first-discharge capacity indicates the insertion of approximately 2.2 Li per HES formula unit, whereas the stabilized reversible capacity in the following cycles corresponds to the extraction/insertion of approximately 2 Li

per formula unit. Thus, a small amount of residual Li remains in the highly charged state, giving an average composition close to $Li_{0.2}HES$. For DFT calculations, this experimentally estimated composition was approximated as $Li_{0.22}HES$ to allow an integer number of Li atoms in the periodic supercell.

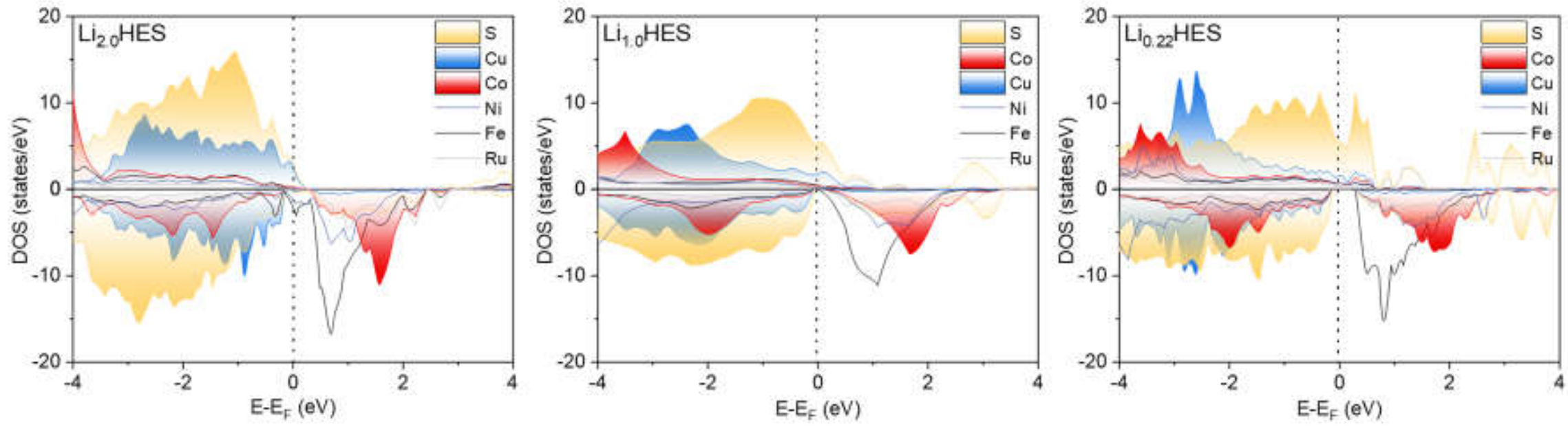


**Fig. S41** Structural and pDOS comparison between $Li_{2.0}HES$, $Li_{1.0}HES$ and $Li_{0.22}HES$.

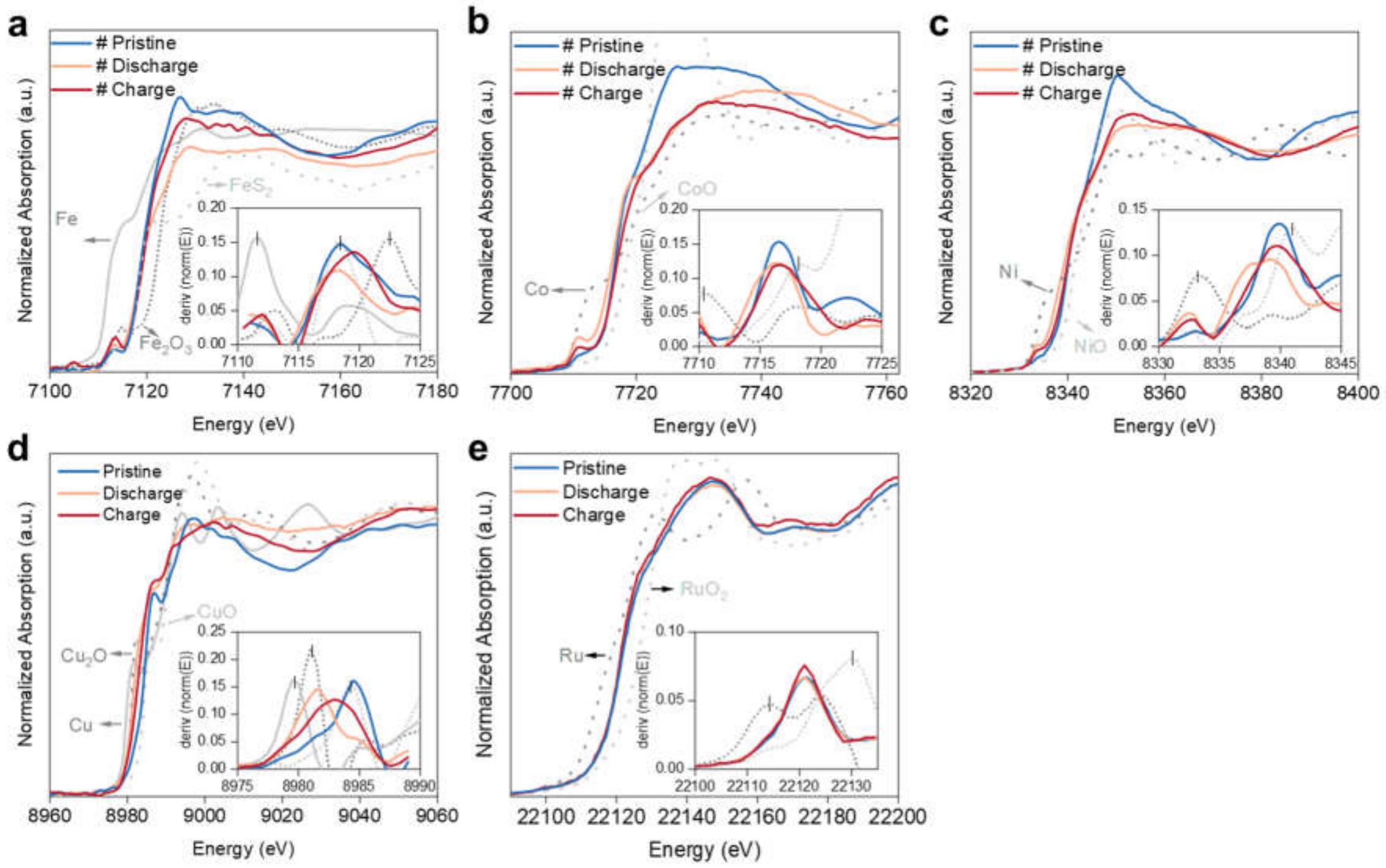


**Fig. S42** Ex situ XANES spectra of Fe (a), Co (b), Ni (c), Cu (d), and Ru (e) in the HES sample and their corresponding reference standards. The insets in each panel show the first-derivative curves of the main-edge region, from which the edge energy $E_0$ is determined.

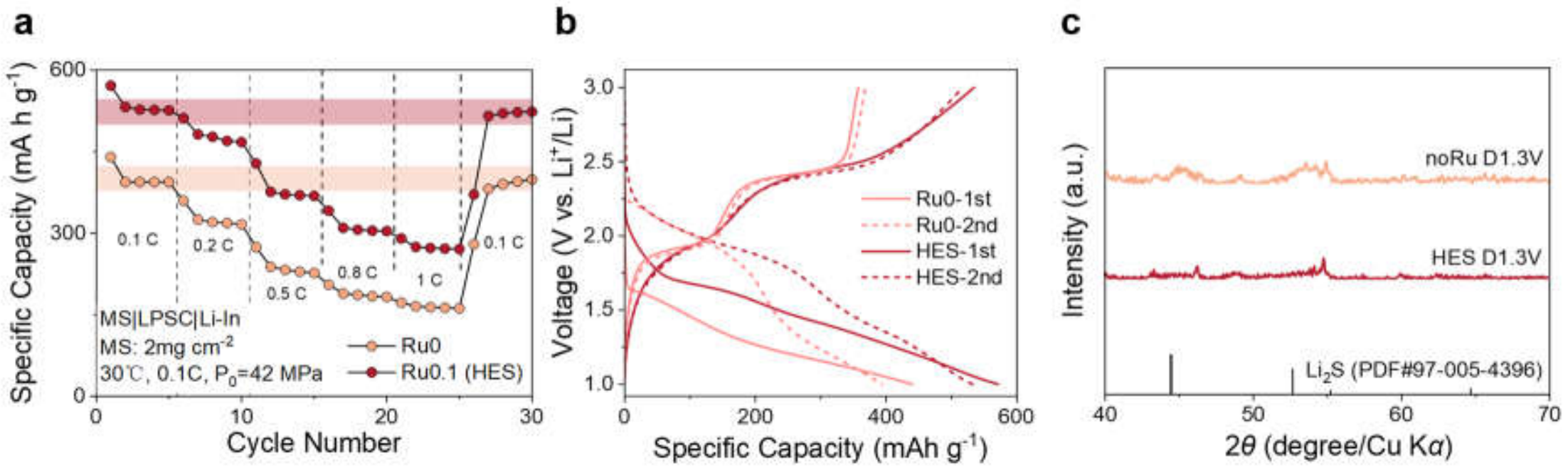


**Fig. S43** Electrochemical performance comparison between ASSLSBs employing HES and Ru-free cathodes. (a) Rate performance. (b) Representative discharge–charge voltage profiles during the initial two cycles. (c) Ex situ XRD patterns of HES and Ru-free cathodes at the discharged state of 1.3 V vs. $Li^+$/Li.

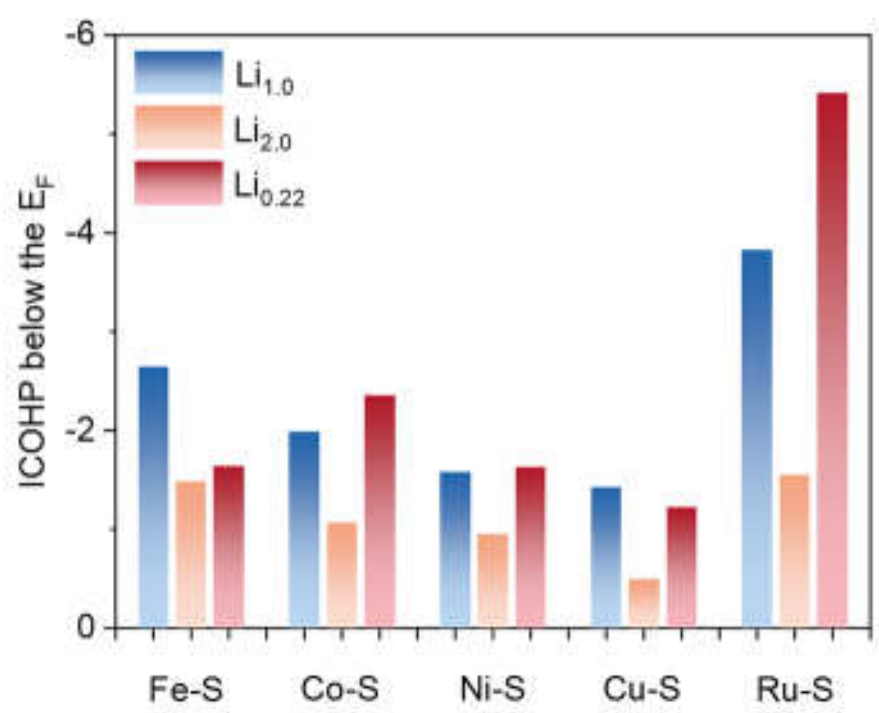


**Fig. S44.** ICOHP value below the Fermi level of HES at different lithiation states.

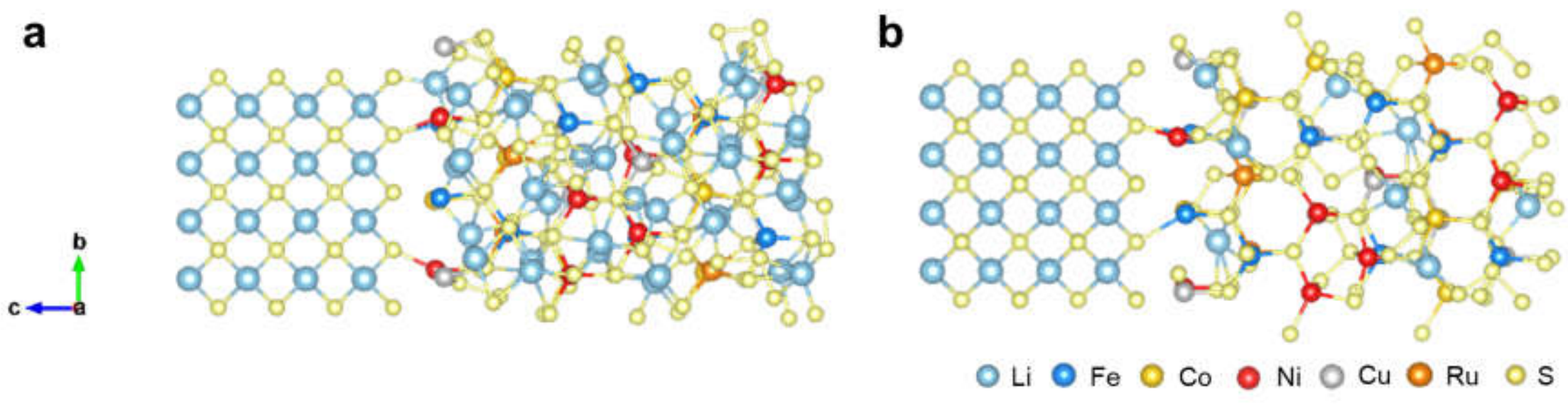


**Fig. S45** Initial heterojunction configurations of $Li_2S$ with $Li_{0.22}$HES (a) and $Li_{1.0}$HES (b). The initial interfacial gap was set to 2 Å and relaxed to 0.76 Å and 0.90 Å for $Li_2S$|$Li_{0.22}$HES and $Li_2S$|$Li_{1.0}$HES, respectively. The results suggest a strong interfacial affinity between $Li_2S$ and lithiated HES, where central metal atoms in HES interact with sulfur in $Li_2S$, thereby lowering the atomic rearrangement barrier.

**Table S1** Comparison of half-cell performance with previous studies.

| Strategy | Sulfur content, wt% | Areal S loading, mg cm$^{-2}$ | Areal current, mA cm$^{-2}$ | C-rate | Initial capacity[a], mAh g$^{-1}$ | Initial areal capacity, mAh cm$^{-2}$ | Cycle number | Capacity retention | T, °C | Year | Ref. |
|---|---|---|---|---|---|---|---|---|---|---|---|
| rGO@S | 12 | 0.45 | 0.75 | 1 | 1077 | 0.42 | 750 | 77% | 60 | 2017 | [22] |
| S@BP2000 | 30 | 0.45 | 2.26 | 3 | 1221 | 0.55 | 500 | 89% | 80 | 2019 | [23] |
| CNTs@S | 13 | 0.5 | 0.84 | 1 | 813 | 0.41 | 400 | 81% | 60 | 2020 | [24] |
| $Mo_6S_8$@S8 | 32.5 | 0.54 | 0.04 | 0.044 | 1050 | 0.57 | 120 | 76% | 70 | 2021 | [25] |
| ESW optimization | 33 | 3.4 | 0.57 | 0.1 | 1325 | 4.5 | 100 | 82% | RT | 2021 | [26] |
| S-$MoS_2$@PPCF | 36 | 3 | 0.54 | 0.1 | 1456 | 4.37 | 220 | 78% | 30 | 2022 | [27] |
| S-PPCF-SE | 40 | 4.5 | 0.8 | 0.1 | 1166 | 5.25 | 200 | 61% | 30 | 2022 | [28] |
| Co@AB/S | 40 | 4.5 | 2.2 | 0.3 | 1315 | 5.92 | 300 | 74% | 60 | 2023 | [29] |
| S/LiI | 36 | 0.65 | 1.3 | 1.2 | 1069 | 0.7 | 1500 | 92% | 25 | 2023 | [30] |
| $S_{9.3}I$ | 40 | 4.2 | 3.36 | 0.48 | 914 | 3.84 | 50 | 90% | 60 | 2024 | [31] |
| S@hCNC39 | 40 | 4.5 | 1.51 | 0.2 | 1344 | 6.05 | 180 | 70% | 60 | 2024 | [32] |
| LPO@AB/S | 40 | 4.5 | 2.2 | 0.3 | 1177 | 5.3 | 100 | 85% | 60 | 2024 | [33] |
| S/NCNT@PPy | 38 | 4.5 | 2.25 | 0.3 | 1553 | 6.8 | 100 | 88% | 60 | 2024 | [34] |
| S@LBPSI | 30 | 1.05 | 3.7 | 2 | 1105 | 1.2 | 5000 | 81% | 25 | 2025 | [35] |
| | 45 | 1.9 | 0.64 | 0.2 | 1049 | 2 | 200 | 100% | 25 | | |
| | 45 | 6.3 | 1.1 | 0.1 | 1413 | 8.9 | 100 | 85% | 25 | | |
| S-C-MIEC20 | 50 | 2 | 6.7 | 2 | 953 | 1.91 | 1000 | 98% | 60 | 2025 | [19] |
| S@LPWSI | 35 | 7.1 | 1.19 | 0.1 | 1253 | 8.9 | 50 | 46% | 25 | 2025 | [36] |
| | 35 | 1.1 | 1.76 | 1 | 618 | 0.68 | 1000 | 122% | 25 | | |
| S/LPSC@LYB-0.25 | 42 | 8 | 2.7 | 0.2 | 630 | 5.04 | 300 | 98% | 30 | 2025 | [37] |
| | 28 | 1.5 | 1.3 | 0.5 | 989 | 1.3 | 500 | 90% | 30 | | |
| MCS-S-3-Co | 29 | 1 | 1.68 | 1 | 708 | 0.7 | 1400 | 85% | 25 | 2025 | [38] |
| | | 4 | 0.67 | 0.1 | 1050 | 4.2 | 100 | 93% | 25 | | |
| a-$TiS_2$ | 17.6 | 1 | 1.675 | 1 | 1385 | 1.38 | 2000 | 52% | 30 | 2025 | [20] |
| | | 4 | 0.67 | 0.1 | 1762 | 7.05 | 150 | 97% | 30 | | |
| S/LPSC/C 2000rpm 5h | 23 | 2 | 0.67 | 0.2 | 1620 | 3.24 | 450 | 93% | 25 | 2025 | [39] |
| | | 4 | 1.4 | 0.2 | 1587 | 6.35 | 450 | 80% | 25 | | |
| S@HES | 30 | 0.6 | 2.05 | 2 | 1130 | 0.69 | 1000 | 84% | 30 | this work | |
| | 30 | 1.15 | 3.84 | 2 | 1576 | 1.81 | 300 | 90% | 30 | | |
| | 30 | 2.68 | 4.4 | 2 | 847 | 2.27 | 300 | 76% | 30 | | |
| | 40 | 1.5 | 2.5 | 1 | 890 | 1.34 | 1000 | 73% | 30 | | |
| | 40 | 3.3 | 5.5 | 1 | 1386 | 4.52 | 400 | 70% | 55 | | |
| | 40 | 6.3 | 10.5 | 1 | 972 | 6.1 | 30 | 94% | 55 | | |
| | 45 | 8.5 | 2.86 | 0.2 | 876.92 | 7.49 | 50 | 107% | 30 | | |
| | 50 | 1.5 | 5.03 | 2 | 673 | 1.03 | 300 | 95% | 30 | | |
| | 50 | 2.5 | 8.37 | 2 | 1101 | 2.75 | 250 | 81.7% | 55 | | |

a. It should be noted that, where specified in the literature, the capacity reported here corresponds to the maximum value achieved after the activation process.

**Table S2** Relative element ratio measured by ICP-OES.

| Sample | Element | Molar ratio |
|---|---|---|
| | Fe | 23 |
| | Co | 21 |
| HES | Ni | 24 |
| | Cu | 20 |
| | Ru | 12 |

For a random solid solution, the ideal configurational entropy per mole can be expressed as:

$$S_{config} = -\mathrm{R}\left[(\sum_{i=1}^{N} x_i ln x_i)_{cation} + \left(\sum_{j=1}^{M} x_j ln x_j\right)_{anion}\right] \qquad \textbf{(Equation S1)}$$

where R is the ideal gas constant and $x_i$ and $x_j$ represents the molar fraction. According to the results obtained from ICP-OES, the HES converted to the following molar ratio: $Fe_{0.23}Co_{0.21}Ni_{0.24}Cu_{0.2}Ru_{0.12}S_2$,

$\Delta S_{conf}$=-R(0.23ln0.23+0.21ln0.21+0.24ln0.24+0.2ln0.2+0.12 ln0.12) =1.58 R

**Table S3** Structural parameters for the $Fe_{0.23}Co_{0.21}Ni_{0.24}Cu_{0.2}Ru_{0.12}S_2$ obtained from Rietveld refinement of the XRD pattern.

| Composition | HES |
|---|---|
| Space group | $Pa\bar{3}$ |
| Phase structure | Cubic |
| a=b=c (Å) | 5.609 (1) |
| α=β=γ (°) | 90 |
| V ($Å^3$) | 176.439 (3) |
| *wRp* | 2.72% |
| GOF | 1.74 |
| Atomic site (Fe) | (0.000, 0.000, 0.000) |
| Atomic site (Co) | (0.000, 0.000, 0.000) |
| Atomic site (Ni) | (0.000, 0.000, 0.000) |
| Atomic site (Cu) | (0.000, 0.000, 0.000) |
| Atomic site (Ru) | (0.000, 0.000, 0.000) |
| Atomic site (S) | (0.112, 0.612, 0.888) |
| Occ. (Fe) | 0.23 |
| Occ. (Co) | 0.21 |
| Occ. (Ni) | 0.24 |
| Occ. (Cu) | 0.2 |
| Occ. (Ru) | 0.12 |
| Occ. (S) | 1 |

**Table S4** Structural parameters for the $FeS_2$ obtained from Rietveld refinement of the XRD pattern.

| Composition | $FeS_2$ |
|---|---|
| Space group | $Pa\bar{3}$ |
| Phase structure | Cubic |
| a=b=c (Å) | 5.41 (1) |
| α=β=γ (°) | 90 |
| V ($Å^3$) | 159.09 (3) |
| *wRp* | 3.01% |
| GOF | 1.14 |
| Atomic site (Fe) | (0.000, 0.000, 0.000) |
| Atomic site (S) | (0.115, 0.615, 0.885) |

**Table S5** Comparison of full-cell performance with previous studies.

| Anode | Sulfur content, wt% | N/P ratio | Areal Capacity, mAh $cm^{-2}$ | Current density, mA $cm^{-2}$ | Energy Density, W h $kg^{-1}$ | S loading, mg $cm^{-2}$ | Ref. |
|---|---|---|---|---|---|---|---|
| Li | 40 | 5.1 | 0.974 | 0.192 | 335.322 | 1.2 | [31] |
| LiAl | 26.25 | 1.125 | 1.8 | 0.108 | 541 | 1.08 | [40] |
| LiB@AgC | 40 | 8.2 | 2.295 | 0.25 | 393.787 | 1.5 | [41] |
| LiIn | 33.3 | 2 | 4.5 | 0.568 | 117.2695 | 3.4 | [26] |
| Li3.75Si | 40 | 1.61 | 4.8 | 0.25 | 570.5 | 3.3 | This work |
| Li3.75Si | 40 | 1.58 | 7.632 | 0.6 | 544.87 | 5.04 | This work |

**Table S6** The Helmholtz free energy of $Li_2HES$ at room temperature.

| | $Li_2HES$ | $Li_2S$ | FeS | CoS | NiS | CuS | Ru | $RuS_2$ |
|---|---|---|---|---|---|---|---|---|
| $E_0$ (kJ/mol) | -1979 | -1157 | -870.4 | -878.1 | -648.6 | -673.1 | -328.6 | -1366 |
| $E_{ZPE}$ (kJ/mol) | 20.05 | 16.15 | 7.364 | 8.211 | 8.365 | 6.942 | 3.433 | 15.87 |
| S (J/(mol*K)) | 0.120 | 0.057 | 0.035 | 0.050 | 0.048 | 0.063 | 0.031 | 0.059 |

Stability assessment: Based on the reaction

[$Li_2(Fe_2Co_2Ni_2Cu_2Ru)_{1/9}S_2 \rightarrow Li_2S+2/9(FeS+CoS+NiS+CuS)+1/18(Ru+RuS_2)$].

The decomposition reaction $\Delta G=E_0$(DFT calculation) $+E_{ZPE}$(Zero-point energy)-$T\cdot\Delta S$(vibrational partition function=53.39 kJ/mol> 0.

## REFERENCES


1. Lv Z, Liu J, Li C, et al. High-areal-capacity all-solid-state Li-S battery enabled by dry process technology. eTransportation 2024; **19**: 100298.

2. Ikezawa A, Fukunishi G, Okajima T *et al.* Performance of $Li_4Ti_5O_{12}$-based reference electrode for the electrochemical analysis of all-solid-state lithium-ion batteries. *Electrochem Commun* 2020; **116**: 106743.

3. Gu J, Chen X, Ma R *et al.* The insight of micro-short circuits caused by electrochemo-mechanical stress crosstalk in all-solid-state Li metal batteries. *Energy Storage Mater* 2023; **63**: 103052.

4. Perdew JP, Burke K and Ernzerhof M. Generalized gradient approximation made simple. *Phys Rev Lett* 1996; **77**: 3865–8.

5. Kresse G and Joubert D. From ultrasoft pseudopotentials to the projector augmented-wave method. *Phys Rev B* 1999; **59**: 1758–75.

6. Blöchl PE. Projector augmented-wave method. *Phys Rev B* 1994; **50**: 17953–17979.

7. Abdullahi YZ. Electronic and magnetic properties of $RuO_2$ monolayer: DFT+U investigation. *Comput Condens Matter* 2021; **29**: e00614.

8. Yang X, Lin M, Zheng G *et al.* Enabling stable high-voltage $LiCoO_2$ operation by using synergetic interfacial modification strategy. *Adv Funct Mater* 2020; **30**: 2004664.

9. Wang L, Maxisch T and Ceder G. Oxidation energies of transition metal oxides within the GGA + U framework. *Phys Rev B* 2006; **73**: 195107.

10. Zunger A, Wei S-H, Ferreira LG *et al*. Special quasirandom structures. *Phys Rev Lett* 1990; **65**: 353–6.

11. Van De Walle A, Tiwary P, De Jong M *et al.* Efficient stochastic generation of special quasirandom structures. *Calphad* 2013; **42**: 13–8.

12. Togo A. First-principles phonon calculations with phonopy and phono3py. *J Phys Soc Jpn* 2023; **92**: 012001.

13. Deringer VL, Tchougr é eff AL, Dronskowski R. Crystal Orbital Hamilton Population (COHP) Analysis As Projected from Plane-Wave Basis Sets. *J Phys Chem A* 2011; **115**: 5461–6.

14. Yu T, Li H, Liu Y, *et al.* A prototype of dual-ion conductor for all-solid-state lithium batteries. *Sci Adv* 2023; **9**: eadj8171.

15. Chen M, Prasada Rao R, Adams S, S. The unusual role of $Li_6PS_5Br$ in all-solid-state $CuS/Li_6PS_5Br/In–Li$ batteries. *Solid State Ionics* 2014; **268**: 300–4.

16. Shin BR, Nam YJ, Kim JW *et al*. Interfacial architecture for extra $Li^+$ storage in all-solid-state lithium batteries. *Sci Rep* 2014; **4**: 5572.

17. Zhang R, Strauss F, Jiang L *et al.* Transition-metal interdiffusion and solid electrolyte poisoning in all-solid-state batteries revealed by cryo-TEM. *Chem Commun* 2023; **59**: 4600-3.

18. Haruyama J, Sodeyama K and Tateyama Y. Cation mixing properties toward Co diffusion at the $LiCoO_2$ cathode/sulfide electrolyte interface in a solid-state battery. *ACS Appl Mater Interfaces* 2017; **9**: 286–92.

19. Wang D, Gwalani B, Wierzbicki D *et al.* Overcoming the conversion reaction

limitation at three-phase interfaces using mixed conductors towards energy-dense solid-state Li–S batteries. *Nat Mater* 2025; **24**: 243–51.
20. Li Q, Xie C, Jiang X *et al.* Catalytic solder fuses solid-solid interfaces for all-solid-state lithium-sulfur batteries. *Adv Mater* 2025; **37**: 2507308.
21. Kim SS, Kitchaev DA, Patheria ES *et al.* Cation vacancies enable anion redox in Li cathodes. *J Am Chem Soc* 2024; **146**: 20951–62.
22. Yao X, Huang N, Han F *et al.* High-performance all-solid-state lithium–sulfur batteries enabled by amorphous sulfur-coated reduced graphene oxide cathodes. *Adv Energy Mater* 2017; **7**: 1602923.
23. Han Q, Li X, Shi X *et al.* Outstanding cycle stability and rate capabilities of the all-solid-state Li–S battery with a $Li_7P_3S_{11}$ glass-ceramic electrolyte and a core–shell S@BP2000 nanocomposite. *J Mater Chem A* 2019; **7**: 3895–902.
24. Zhang Q, Huang N, Huang Z *et al.* CNTs@S composite as cathode for all-solid-state lithium-sulfur batteries with ultralong cycle life. *J Energy Chem* 2020; **40**: 151–5.
25. Li M, Liu T, Shi Z *et al.* Dense All-electrochem-active electrodes for all-solid-state lithium batteries. *Adv Mater* 2021; **33**: 2008723.
26. Ohno S, Rosenbach C, Dewald GF *et al*. Linking solid electrolyte degradation to charge carrier transport in the thiophosphate-based composite cathode toward solid-state lithium-sulfur batteries. *Adv Funct Mater* 2021; **31**: 2010620.
27. Sun X, Cao D, Wang Y *et al.* All-solid-state Li–S batteries enhanced by interface stabilization and reaction kinetics promotion through 2D transition metal sulfides. *Adv Mater Inter* 2022; **9**: 2200539.
28. Sun X, Li Q, Cao D *et al.* High surface area N-doped carbon fibers with accessible reaction sites for all-solid-state lithium-sulfur batteries. *Small* 2022; **18**: 2105678.
29. Zhong H, Su Y, Wu Y *et al.* Long-life and high-loading all-solid-state Li–S batteries enabled by acetylene black with dispersed Co-$N_4$ as single atom catalyst. *Adv Energy Mater* 2023; **13**: 2300767.
30. Kim JT, Rao A, Nie H-Y *et al.* Manipulating $Li_2S_2$/$Li_2S$ mixed discharge products of all-solid-state lithium sulfur batteries for improved cycle life. *Nat Commun* 2023; **14**: 6404.
31. Zhou J, Holekevi Chandrappa ML, Tan S *et al.* Healable and conductive sulfur iodide for solid-state Li–S batteries. *Nature* 2024; **627**: 301-5.
32. Luo Y, Pan S, Tian J *et al.* Engineering triple-phase interfaces with hierarchical carbon nanocages for high-areal-capacity all-solid-state Li-S batteries. *Adv Mater* 2024; **36**: 2413325.
33. Zhong H, Su Y, Ma R *et al.* Nano-scale interface engineering of sulfur cathode to enable high-performance all-solid-state Li–S batteries. *Adv Funct Mater* 2024; **34**: 2315925.
34. Ma R, Fan Y, Jin Y *et al*. Construction of elastic and conductive channels for high-rate and high-areal-capacity sulfur cathodes in all-solid-state lithium–sulfur batteries. *Adv Energy Mater* 2024; **14**: 2304412.
35. Song H, Münch K, Liu X *et al.* All-solid-state Li–S batteries with fast solid–solid sulfur reaction. *Nature* 2025; **637**: 846–53.
36. Shen K, Shi W, Song H *et al.* Solid catholyte with regulated interphase redox for

all-solid-state lithium-sulfur batteries. *Adv Mater* 2025; **37**: 2417171.
37. Wang M, Su H, Zhong Y *et al.* A unitized encapsulation architecture with durable epitaxial ion-conductive scaffolds for ultrastable solid-state sulfur cathode. *Energy Environ Sci* 2025; **18**: 4162-75.
38. Cao Y, Geng C, Bai C *et al.* Integrating solid interfaces for catalysis in all-solid-state lithium–sulfur batteries. *Energy Environ Sci* 2025; **18**: 3795-806.
39. Lee J, Zhou S, Ferrari VC *et al.* Halide segregation to boost all-solid-state lithium-chalcogen batteries. *Science* 2025; **388**: 724–9.
40. Pan H, Zhang M, Cheng Z *et al.* Carbon-free and binder-free Li-Al alloy anode enabling an all-solid-state Li-S battery with high energy and stability. *Sci Adv* 2022; **8**: eabn4372.
41. Chen Z, Liang Z, Zhong H *et al.* Bulk/Interfacial synergetic approaches enable the stable anode for high energy density all-solid-state lithium–sulfur batteries. *ACS Energy Lett* 2022; **7**: 2761–70.
